# Supplementary material for: An Integrated Transcriptome and Proteome Analysis Reveals Putative Regulators of Adventitious Root Formation in Taxodium ‘Zhongshanshan’
Source: Int J Mol Sci. 2019 Mar 11;20(5):1225. doi: 10.3390/ijms20051225 (PMC6429173; doi:10.3390/ijms20051225)
Supplement: Supplementary file 1 [file ijms-20-01225-s001.zip › Supplementary material20190227/Table S10.docx]

**Table S10** The result of KEGG pathway classification and functional enrichment of S1-VS-S2_3 and S1-VS-S2_7

|  |  |  | S1-VS-S2_3 Pathway Enrichment |  |  |  |
| --- | --- | --- | --- | --- | --- | --- |
|  | **Pathway** | **DEGs genes with pathway annotation (252)** | **All genes with pathway annotation (20073)** | **Pvalue** | **Qvalue** | **Pathway ID** |
| 1 | Phenylpropanoid biosynthesis | 30 (11.9%) | 819 (4.08%) | 0 | 0.000014 | ko00940 |
| 2 | DNA replication | 9 (3.57%) | 136 (0.68%) | 0.000056 | 0.002616 | ko03030 |
| 3 | Other glycan degradation | 10 (3.97%) | 213 (1.06%) | 0.000376 | 0.011649 | ko00511 |
| 4 | Glutathione metabolism | 10 (3.97%) | 271 (1.35%) | 0.002332 | 0.054216 | ko00480 |
| 5 | Limonene and pinene degradation | 6 (2.38%) | 114 (0.57%) | 0.003183 | 0.059196 | ko00903 |
| 6 | Cyanoamino acid metabolism | 9 (3.57%) | 252 (1.26%) | 0.004704 | 0.072919 | ko00460 |
| 7 | Stilbenoid, diarylheptanoid and gingerol biosynthesis | 7 (2.78%) | 192 (0.96%) | 0.010894 | 0.125079 | ko00945 |
| 8 | Ascorbate and aldarate metabolism | 8 (3.17%) | 247 (1.23%) | 0.013063 | 0.125079 | ko00053 |
| 9 | Flavonoid biosynthesis | 10 (3.97%) | 352 (1.75%) | 0.013853 | 0.125079 | ko00941 |
| 10 | Phenylalanine metabolism | 7 (2.78%) | 208 (1.04%) | 0.016293 | 0.125079 | ko00360 |
| 11 | Cutin, suberine and wax biosynthesis | 5 (1.98%) | 118 (0.59%) | 0.016664 | 0.125079 | ko00073 |
| 12 | Glycosphingolipid biosynthesis - ganglio series | 3 (1.19%) | 44 (0.22%) | 0.017735 | 0.125079 | ko00604 |
| 13 | Glycosaminoglycan degradation | 4 (1.59%) | 80 (0.4%) | 0.018239 | 0.125079 | ko00531 |
| 14 | Indole alkaloid biosynthesis | 3 (1.19%) | 45 (0.22%) | 0.018829 | 0.125079 | ko00901 |
| 15 | Sulfur metabolism | 4 (1.59%) | 92 (0.46%) | 0.028717 | 0.172058 | ko00920 |
| 16 | Carotenoid biosynthesis | 6 (2.38%) | 185 (0.92%) | 0.029601 | 0.172058 | ko00906 |
|  |  |  | **S1-VS-S2_7 Pathway Enrichment** |  |  |  |
|  | **Pathway** | **DEGs genes with pathway annotation (199)** | **All genes with pathway annotation (20073)** | **Pvalue** | **Qvalue** | **Pathway ID** |
| 1 | Phenylpropanoid biosynthesis | 22 (11.06%) | 819 (4.08%) | 0.000022 | 0.000981 | ko00940 |
| 2 | Cutin, suberine and wax biosynthesis | 8 (4.02%) | 118 (0.59%) | 0.000023 | 0.000981 | ko00073 |
| 3 | Betalain biosynthesis | 4 (2.01%) | 27 (0.13%) | 0.000138 | 0.003852 | ko00965 |
| 4 | Vitamin B6 metabolism | 5 (2.51%) | 57 (0.28%) | 0.000251 | 0.00527 | ko00750 |
| 5 | Flavonoid biosynthesis | 11 (5.53%) | 352 (1.75%) | 0.000809 | 0.013584 | ko00941 |
| 6 | Photosynthesis | 5 (2.51%) | 98 (0.49%) | 0.002936 | 0.041102 | ko00195 |
| 7 | Glycosphingolipid biosynthesis - globo series | 4 (2.01%) | 65 (0.32%) | 0.00396 | 0.047516 | ko00603 |
| 8 | Tyrosine metabolism | 7 (3.52%) | 225 (1.12%) | 0.007295 | 0.076598 | ko00350 |
| 9 | Carbon fixation in photosynthetic organisms | 7 (3.52%) | 267 (1.33%) | 0.017413 | 0.16252 | ko00710 |
| 10 | Monoterpenoid biosynthesis | 3 (1.51%) | 60 (0.3%) | 0.021722 | 0.182469 | ko00902 |
| 11 | Limonene and pinene degradation | 4 (2.01%) | 114 (0.57%) | 0.026898 | 0.205401 | ko00903 |
| 12 | Nitrogen metabolism | 5 (2.51%) | 184 (0.92%) | 0.036635 | 0.256441 | ko00910 |
| 13 | Photosynthesis - antenna proteins | 2 (1.01%) | 33 (0.16%) | 0.04223 | 0.272874 | ko00196 |
