# Supplementary material for: An Integrated Transcriptome and Proteome Analysis Reveals Putative Regulators of Adventitious Root Formation in Taxodium ‘Zhongshanshan’
Source: Int J Mol Sci. 2019 Mar 11;20(5):1225. doi: 10.3390/ijms20051225 (PMC6429173; doi:10.3390/ijms20051225)
Supplement: Supplementary file 1 [file ijms-20-01225-s001.zip › Supplementary material20190227/Table S11.docx]

**Table S11** The result of KEGG pathway classification and functional enrichment of S2-VS-S3_3 and S2-VS-S3_7

|  |  |  | S2-VS-S3_3 Pathway Enrichment |  |  |  |
| --- | --- | --- | --- | --- | --- | --- |
|  | **Pathway** | **DEGs genes with pathway annotation (19)** | **All genes with pathway annotation (20073)** | **Pvalue** | **Qvalue** | **Pathway ID** |
| 1 | Photosynthesis - antenna proteins | 3 (15.79%) | 33 (0.16%) | 0.000004 | 0.000069 | ko00196 |
| 2 | Photosynthesis | 3 (15.79%) | 98 (0.49%) | 0.000103 | 0.00093 | ko00195 |
| 3 | Cyanoamino acid metabolism | 2 (10.53%) | 252 (1.26%) | 0.023324 | 0.139946 | ko00460 |
|  |  |  | **S2-VS-S3_7 Pathway Enrichment** |  |  |  |
|  | **Pathway** | **DEGs genes with pathway annotation (13)** | **All genes with pathway annotation (20073)** | **Pvalue** | **Qvalue** | **Pathway ID** |
| 1 | 2-Oxocarboxylic acid metabolism | 2 (15.38%) | 222 (1.11%) | 0.008765 | 0.156158 | ko01210 |
| 2 | Glucosinolate biosynthesis | 1 (7.69%) | 27 (0.13%) | 0.017351 | 0.156158 | ko00966 |
