# Supplementary material for: An Integrated Transcriptome and Proteome Analysis Reveals Putative Regulators of Adventitious Root Formation in Taxodium ‘Zhongshanshan’
Source: Int J Mol Sci. 2019 Mar 11;20(5):1225. doi: 10.3390/ijms20051225 (PMC6429173; doi:10.3390/ijms20051225)
Supplement: Supplementary file 1 [file ijms-20-01225-s001.zip › Supplementary material20190227/Table S12.docx]

**Table S12.** Primer sequences of the genes for qRT-PCR.

| Gene | Forward PCR Primer (5ˊ-3ˊ) | Reverse PCR Primer (5ˊ-3ˊ) |
| --- | --- | --- |
| *APRT* | TCCACAGGTTCTTGAATCGCT | TGACTTGAGCCTCATTCGCTC |
| CL6552.Contig3_All | TTCTAGCGATGGCGGCAGTG | TGATCAAGGTGAGGCCCGGA |
| CL1009.Contig3_All | GACGGGCCAGAGGATTCAGC | GCCCTGGGCAGGATCACAAA |
| CL6552.Contig5_All | TGAGCTGGAACCTGTTGGGC | TGCCACCGCTTTCCCCTTTT |
| CL1009.Contig1_All | GGGCCAGAGGATTTGGCTGT | TGGACACAGTGAGGCCGAGA |
| CL633.Contig10_All | ACGTCGTCGGAGAAGGTGGA | CGCGCCCTGCAATCACAAAA |
| CL2792.Contig2_All | ACCCCAGACGTCTCAGTCCC | CGCGCTTCCAATGCAGAACC |
| Unigene19016_All | GCGCCTTTACAGGGAGTGGG | AGCCCGGCAAATCTGTGTCC |
| Unigene12712_All | AGCTTGGCTGTGCTCGTGTT | GGCGAGAATACTGCAGGCGT |
| Unigene12710_All | GCGAGGTCTCAAGTTGGCGT | GCCAGAATGCTGCATGGCAC |
| Unigene32249_All | TGGCGCCTTCACTCCTACCT | TGGCAGAGCCATCTGCAAGG |
| CL1763.Contig3_All | ACCAAAAAGCGCGGGATTGC | TTCCTGTAGCTGTTGCCGCC |
| CL3739.Contig9_All | ACAGGCAAACGTGGGGAACC | GCTGCAGGTGCATGGTCTCA |
