# Supplementary material for: An Integrated Transcriptome and Proteome Analysis Reveals Putative Regulators of Adventitious Root Formation in Taxodium ‘Zhongshanshan’
Source: Int J Mol Sci. 2019 Mar 11;20(5):1225. doi: 10.3390/ijms20051225 (PMC6429173; doi:10.3390/ijms20051225)
Supplement: Supplementary file 1 [file ijms-20-01225-s001.zip › Supplementary material20190227/Table S2.docx]

**Table S2** The result of Gene Ontology (GO) classification and functional enrichment of DEGs

|  |  | Terms for S0-VS-S1._Cellular Component |  |  |
| --- | --- | --- | --- | --- |
|  | **Gene Ontology term** | **Cluster frequency** | **Genome frequency of use** | **Corrected P-value** |
| 1 | extracellular region (view genes) | 24 out of 307 genes, 7.8% | 477 out of 13659 genes, 3.5% | 0.02601 |
| 2 | extracellular space (view genes) | 4 out of 307 genes, 1.3% | 21 out of 13659 genes, 0.2% | 0.1439 |
| 3 | photosystem I (view genes) | 6 out of 307 genes, 2.0% | 62 out of 13659 genes, 0.5% | 0.34105 |
| 4 | central vacuole (view genes) | 2 out of 307 genes, 0.7% | 4 out of 13659 genes, 0.0% | 0.38118 |
| 5 | chloroplast thylakoid (view genes) | 9 out of 307 genes, 2.9% | 135 out of 13659 genes, 1.0% | 0.44631 |
| 6 | plastid thylakoid (view genes) | 9 out of 307 genes, 2.9% | 136 out of 13659 genes, 1.0% | 0.46876 |
|  |  | **Terms for S0-VS-S1._Molecular Function** |  |  |
|  | **Gene Ontology term** | **Cluster frequency** | **Genome frequency of use** | **Corrected P-value** |
| 1 | carboxy-lyase activity (view genes) | 34 out of 481 genes, 7.1% | 140 out of 18328 genes, 0.8% | 4.12E-21 |
| 2 | carbon-carbon lyase activity (view genes) | 37 out of 481 genes, 7.7% | 187 out of 18328 genes, 1.0% | 9.62E-20 |
| 3 | thiamine pyrophosphate binding (view genes) | 18 out of 481 genes, 3.7% | 45 out of 18328 genes, 0.2% | 5.47E-15 |
| 4 | metal ion binding (view genes) | 146 out of 481 genes, 30.4% | 2842 out of 18328 genes, 15.5% | 1.83E-14 |
| 5 | cation binding (view genes) | 147 out of 481 genes, 30.6% | 2912 out of 18328 genes, 15.9% | 6.40E-14 |
| 6 | vitamin binding (view genes) | 21 out of 481 genes, 4.4% | 78 out of 18328 genes, 0.4% | 1.35E-13 |
| 7 | lyase activity (view genes) | 51 out of 481 genes, 10.6% | 535 out of 18328 genes, 2.9% | 3.27E-13 |
| 8 | sulfur compound binding (view genes) | 18 out of 481 genes, 3.7% | 71 out of 18328 genes, 0.4% | 5.28E-11 |
| 9 | oxidoreductase activity, acting on paired donors, with incorporation or reduction of molecular oxygen (view genes) | 34 out of 481 genes, 7.1% | 301 out of 18328 genes, 1.6% | 1.93E-10 |
| 10 | tetrapyrrole binding (view genes) | 38 out of 481 genes, 7.9% | 377 out of 18328 genes, 2.1% | 2.99E-10 |
| 11 | monooxygenase activity (view genes) | 29 out of 481 genes, 6.0% | 250 out of 18328 genes, 1.4% | 5.06E-09 |
| 12 | iron ion binding (view genes) | 31 out of 481 genes, 6.4% | 285 out of 18328 genes, 1.6% | 5.43E-09 |
| 13 | heme binding (view genes) | 33 out of 481 genes, 6.9% | 329 out of 18328 genes, 1.8% | 1.08E-08 |
| 14 | cofactor binding (view genes) | 46 out of 481 genes, 9.6% | 628 out of 18328 genes, 3.4% | 8.35E-08 |
| 15 | ion binding (view genes) | 203 out of 481 genes, 42.2% | 5443 out of 18328 genes, 29.7% | 5.84E-07 |
| 16 | coenzyme binding (view genes) | 37 out of 481 genes, 7.7% | 475 out of 18328 genes, 2.6% | 9.45E-07 |
| 17 | oxidoreductase activity (view genes) | 116 out of 481 genes, 24.1% | 2679 out of 18328 genes, 14.6% | 4.00E-06 |
| 18 | phosphoenolpyruvate carboxykinase (ATP) activity (view genes) | 7 out of 481 genes, 1.5% | 15 out of 18328 genes, 0.1% | 1.06E-05 |
| 19 | magnesium ion binding (view genes) | 18 out of 481 genes, 3.7% | 171 out of 18328 genes, 0.9% | 0.00014 |
| 20 | transition metal ion binding (view genes) | 56 out of 481 genes, 11.6% | 1100 out of 18328 genes, 6.0% | 0.00036 |
| 21 | glutamate decarboxylase activity (view genes) | 7 out of 481 genes, 1.5% | 24 out of 18328 genes, 0.1% | 0.00046 |
| 22 | argininosuccinate synthase activity (view genes) | 4 out of 481 genes, 0.8% | 6 out of 18328 genes, 0.0% | 0.00162 |
| 23 | pheophorbide a oxygenase activity (view genes) | 3 out of 481 genes, 0.6% | 3 out of 18328 genes, 0.0% | 0.00432 |
| 24 | methionine adenosyltransferase activity (view genes) | 7 out of 481 genes, 1.5% | 35 out of 18328 genes, 0.2% | 0.00703 |
| 25 | phosphoenolpyruvate carboxykinase activity (view genes) | 7 out of 481 genes, 1.5% | 38 out of 18328 genes, 0.2% | 0.01233 |
| 26 | xyloglucan:xyloglucosyl transferase activity (view genes) | 7 out of 481 genes, 1.5% | 40 out of 18328 genes, 0.2% | 0.01741 |
| 27 | 6-phosphofructokinase activity (view genes) | 6 out of 481 genes, 1.2% | 28 out of 18328 genes, 0.2% | 0.01759 |
| 28 | phosphofructokinase activity (view genes) | 6 out of 481 genes, 1.2% | 32 out of 18328 genes, 0.2% | 0.03871 |
| 29 | chlorophyllide a oxygenase [overall] activity (view genes) | 3 out of 481 genes, 0.6% | 5 out of 18328 genes, 0.0% | 0.04162 |
| 30 | oxidoreductase activity, acting on single donors with incorporation of molecular oxygen, incorporation of one atom of oxygen (internal monooxygenases or internal mixed function oxidases) (view genes) | 3 out of 481 genes, 0.6% | 6 out of 18328 genes, 0.0% | 0.08161 |
| 31 | pyruvate decarboxylase activity (view genes) | 2 out of 481 genes, 0.4% | 2 out of 18328 genes, 0.0% | 0.16565 |
| 32 | alpha-galactosidase activity (view genes) | 6 out of 481 genes, 1.2% | 45 out of 18328 genes, 0.2% | 0.26094 |
| 33 | galactosidase activity (view genes) | 7 out of 481 genes, 1.5% | 65 out of 18328 genes, 0.4% | 0.3702 |
| 34 | binding (view genes) | 291 out of 481 genes, 60.5% | 9870 out of 18328 genes, 53.9% | 0.40935 |
| 35 | NAD binding (view genes) | 10 out of 481 genes, 2.1% | 125 out of 18328 genes, 0.7% | 0.40954 |
| 36 | UDP-glucose 6-dehydrogenase activity (view genes) | 3 out of 481 genes, 0.6% | 10 out of 18328 genes, 0.1% | 0.4527 |
| 37 | glyceraldehyde-3-phosphate dehydrogenase (NAD+) (phosphorylating) activity (view genes) | 3 out of 481 genes, 0.6% | 10 out of 18328 genes, 0.1% | 0.4527 |
| 38 | oxidoreductase activity, acting on iron-sulfur proteins as donors (view genes) | 3 out of 481 genes, 0.6% | 10 out of 18328 genes, 0.1% | 0.4527 |
| 39 | alcohol dehydrogenase (NAD) activity (view genes) | 2 out of 481 genes, 0.4% | 3 out of 18328 genes, 0.0% | 0.48829 |
|  |  | **Terms for S0-VS-S1._Biological Process** |  |  |
|  | **Gene Ontology term** | **Cluster frequency** | **Genome frequency of use** | **Corrected P-value** |
| 1 | oxidation-reduction process (view genes) | 90 out of 388 genes, 23.2% | 1886 out of 15514 genes, 12.2% | 3.31E-07 |
| 2 | single-organism metabolic process (view genes) | 168 out of 388 genes, 43.3% | 4551 out of 15514 genes, 29.3% | 1.16E-06 |
| 3 | glucose metabolic process (view genes) | 14 out of 388 genes, 3.6% | 86 out of 15514 genes, 0.6% | 1.31E-05 |
| 4 | glutamine family amino acid metabolic process (view genes) | 14 out of 388 genes, 3.6% | 118 out of 15514 genes, 0.8% | 0.00073 |
| 5 | single-organism carbohydrate catabolic process (view genes) | 17 out of 388 genes, 4.4% | 195 out of 15514 genes, 1.3% | 0.00418 |
| 6 | glutamate metabolic process (view genes) | 8 out of 388 genes, 2.1% | 44 out of 15514 genes, 0.3% | 0.00577 |
| 7 | pyruvate metabolic process (view genes) | 15 out of 388 genes, 3.9% | 161 out of 15514 genes, 1.0% | 0.00643 |
| 8 | glycolytic process (view genes) | 14 out of 388 genes, 3.6% | 142 out of 15514 genes, 0.9% | 0.00656 |
| 9 | ATP generation from ADP (view genes) | 14 out of 388 genes, 3.6% | 142 out of 15514 genes, 0.9% | 0.00656 |
| 10 | ADP metabolic process (view genes) | 14 out of 388 genes, 3.6% | 143 out of 15514 genes, 0.9% | 0.00711 |
| 11 | purine nucleoside diphosphate metabolic process (view genes) | 15 out of 388 genes, 3.9% | 163 out of 15514 genes, 1.1% | 0.00746 |
| 12 | purine ribonucleoside diphosphate metabolic process (view genes) | 15 out of 388 genes, 3.9% | 163 out of 15514 genes, 1.1% | 0.00746 |
| 13 | ribonucleoside diphosphate metabolic process (view genes) | 15 out of 388 genes, 3.9% | 163 out of 15514 genes, 1.1% | 0.00746 |
| 14 | defense response to bacterium, incompatible interaction (view genes) | 3 out of 388 genes, 0.8% | 3 out of 15514 genes, 0.0% | 0.0078 |
| 15 | hexose metabolic process (view genes) | 14 out of 388 genes, 3.6% | 145 out of 15514 genes, 0.9% | 0.00833 |
| 16 | gluconeogenesis (view genes) | 7 out of 388 genes, 1.8% | 35 out of 15514 genes, 0.2% | 0.0107 |
| 17 | S-adenosylmethionine biosynthetic process (view genes) | 7 out of 388 genes, 1.8% | 35 out of 15514 genes, 0.2% | 0.0107 |
| 18 | nucleoside diphosphate phosphorylation (view genes) | 14 out of 388 genes, 3.6% | 149 out of 15514 genes, 1.0% | 0.01134 |
| 19 | hexose biosynthetic process (view genes) | 7 out of 388 genes, 1.8% | 36 out of 15514 genes, 0.2% | 0.01301 |
| 20 | monosaccharide biosynthetic process (view genes) | 7 out of 388 genes, 1.8% | 37 out of 15514 genes, 0.2% | 0.0157 |
| 21 | S-adenosylmethionine metabolic process (view genes) | 7 out of 388 genes, 1.8% | 39 out of 15514 genes, 0.3% | 0.02247 |
| 22 | nucleoside diphosphate metabolic process (view genes) | 15 out of 388 genes, 3.9% | 179 out of 15514 genes, 1.2% | 0.02249 |
| 23 | nucleotide phosphorylation (view genes) | 14 out of 388 genes, 3.6% | 160 out of 15514 genes, 1.0% | 0.02508 |
| 24 | glycolytic process through fructose-6-phosphate (view genes) | 6 out of 388 genes, 1.5% | 28 out of 15514 genes, 0.2% | 0.02797 |
| 25 | cofactor metabolic process (view genes) | 25 out of 388 genes, 6.4% | 425 out of 15514 genes, 2.7% | 0.03569 |
| 26 | xyloglucan metabolic process (view genes) | 7 out of 388 genes, 1.8% | 43 out of 15514 genes, 0.3% | 0.0432 |
| 27 | one-carbon metabolic process (view genes) | 9 out of 388 genes, 2.3% | 74 out of 15514 genes, 0.5% | 0.04615 |
|  |  | **Terms for S1-VS-S2._Cellular Component** |  |  |
|  | **Gene Ontology term** | **Cluster frequency** | **Genome frequency of use** | **Corrected P-value** |
| 1 | extracellular region (view genes) | 46 out of 323 genes, 14.2% | 477 out of 13659 genes, 3.5% | 4.07E-14 |
| 2 | ribosome (view genes) | 67 out of 323 genes, 20.7% | 1106 out of 13659 genes, 8.1% | 6.26E-11 |
| 3 | ribosomal subunit (view genes) | 37 out of 323 genes, 11.5% | 530 out of 13659 genes, 3.9% | 5.04E-07 |
| 4 | intracellular ribonucleoprotein complex (view genes) | 71 out of 323 genes, 22.0% | 1492 out of 13659 genes, 10.9% | 7.28E-07 |
| 5 | ribonucleoprotein complex (view genes) | 71 out of 323 genes, 22.0% | 1492 out of 13659 genes, 10.9% | 7.28E-07 |
| 6 | non-membrane-bounded organelle (view genes) | 75 out of 323 genes, 23.2% | 1719 out of 13659 genes, 12.6% | 1.00E-05 |
| 7 | intracellular non-membrane-bounded organelle (view genes) | 75 out of 323 genes, 23.2% | 1719 out of 13659 genes, 12.6% | 1.00E-05 |
| 8 | cytosolic ribosome (view genes) | 26 out of 323 genes, 8.0% | 387 out of 13659 genes, 2.8% | 0.00025 |
| 9 | cytosolic part (view genes) | 27 out of 323 genes, 8.4% | 425 out of 13659 genes, 3.1% | 0.00045 |
| 10 | small ribosomal subunit (view genes) | 19 out of 323 genes, 5.9% | 252 out of 13659 genes, 1.8% | 0.00128 |
| 11 | cytosolic large ribosomal subunit (view genes) | 15 out of 323 genes, 4.6% | 198 out of 13659 genes, 1.4% | 0.01079 |
| 12 | large ribosomal subunit (view genes) | 18 out of 323 genes, 5.6% | 278 out of 13659 genes, 2.0% | 0.01636 |
| 13 | external side of plasma membrane (view genes) | 2 out of 323 genes, 0.6% | 2 out of 13659 genes, 0.0% | 0.08139 |
| 14 | cell surface (view genes) | 2 out of 323 genes, 0.6% | 3 out of 13659 genes, 0.0% | 0.24036 |
| 15 | cytosolic small ribosomal subunit (view genes) | 11 out of 323 genes, 3.4% | 168 out of 13659 genes, 1.2% | 0.31876 |
| 16 | macromolecular complex (view genes) | 87 out of 323 genes, 26.9% | 2779 out of 13659 genes, 20.3% | 0.34777 |
|  |  | **Terms for S1-VS-S2._Molecular Function** |  |  |
|  | **Gene Ontology term** | **Cluster frequency** | **Genome frequency of use** | **Corrected P-value** |
| 1 | oxidoreductase activity (view genes) | 131 out of 464 genes, 28.2% | 2679 out of 18328 genes, 14.6% | 3.28E-12 |
| 2 | peroxidase activity (view genes) | 26 out of 464 genes, 5.6% | 194 out of 18328 genes, 1.1% | 9.47E-10 |
| 3 | structural constituent of ribosome (view genes) | 66 out of 464 genes, 14.2% | 1053 out of 18328 genes, 5.7% | 1.80E-09 |
| 4 | oxidoreductase activity, acting on peroxide as acceptor (view genes) | 26 out of 464 genes, 5.6% | 204 out of 18328 genes, 1.1% | 3.03E-09 |
| 5 | heme binding (view genes) | 33 out of 464 genes, 7.1% | 329 out of 18328 genes, 1.8% | 4.14E-09 |
| 6 | tetrapyrrole binding (view genes) | 35 out of 464 genes, 7.5% | 377 out of 18328 genes, 2.1% | 8.83E-09 |
| 7 | structural molecule activity (view genes) | 69 out of 464 genes, 14.9% | 1198 out of 18328 genes, 6.5% | 2.52E-08 |
| 8 | antioxidant activity (view genes) | 26 out of 464 genes, 5.6% | 234 out of 18328 genes, 1.3% | 6.64E-08 |
| 9 | carbon-oxygen lyase activity, acting on polysaccharides (view genes) | 10 out of 464 genes, 2.2% | 29 out of 18328 genes, 0.2% | 3.05E-07 |
| 10 | pectate lyase activity (view genes) | 10 out of 464 genes, 2.2% | 29 out of 18328 genes, 0.2% | 3.05E-07 |
| 11 | taxane 10-beta-hydroxylase activity (view genes) | 5 out of 464 genes, 1.1% | 13 out of 18328 genes, 0.1% | 0.00264 |
| 12 | phosphate transmembrane transporter activity (view genes) | 9 out of 464 genes, 1.9% | 57 out of 18328 genes, 0.3% | 0.00289 |
| 13 | inorganic phosphate transmembrane transporter activity (view genes) | 6 out of 464 genes, 1.3% | 26 out of 18328 genes, 0.1% | 0.00911 |
| 14 | oxidoreductase activity, acting on single donors with incorporation of molecular oxygen (view genes) | 10 out of 464 genes, 2.2% | 83 out of 18328 genes, 0.5% | 0.01093 |
| 15 | naringenin-chalcone synthase activity (view genes) | 3 out of 464 genes, 0.6% | 4 out of 18328 genes, 0.0% | 0.01512 |
| 16 | monooxygenase activity (view genes) | 18 out of 464 genes, 3.9% | 250 out of 18328 genes, 1.4% | 0.01729 |
| 17 | metal ion binding (view genes) | 103 out of 464 genes, 22.2% | 2842 out of 18328 genes, 15.5% | 0.01774 |
| 18 | cation binding (view genes) | 104 out of 464 genes, 22.4% | 2912 out of 18328 genes, 15.9% | 0.02895 |
|  |  |  |  |  |
|  |  | **Terms for S1-VS-S2._Biological Process** |  |  |
|  | **Gene Ontology term** | **Cluster frequency** | **Genome frequency of use** | **Corrected P-value** |
| 1 | hydrogen peroxide catabolic process (view genes) | 19 out of 394 genes, 4.8% | 112 out of 15514 genes, 0.7% | 3.22E-08 |
| 2 | hydrogen peroxide metabolic process (view genes) | 19 out of 394 genes, 4.8% | 119 out of 15514 genes, 0.8% | 9.60E-08 |
| 3 | detoxification (view genes) | 24 out of 394 genes, 6.1% | 209 out of 15514 genes, 1.3% | 4.32E-07 |
| 4 | cellular oxidant detoxification (view genes) | 24 out of 394 genes, 6.1% | 209 out of 15514 genes, 1.3% | 4.32E-07 |
| 5 | cellular detoxification (view genes) | 24 out of 394 genes, 6.1% | 209 out of 15514 genes, 1.3% | 4.32E-07 |
| 6 | response to toxic substance (view genes) | 24 out of 394 genes, 6.1% | 210 out of 15514 genes, 1.4% | 4.77E-07 |
| 7 | reactive oxygen species metabolic process (view genes) | 19 out of 394 genes, 4.8% | 152 out of 15514 genes, 1.0% | 6.58E-06 |
| 8 | catabolic process (view genes) | 60 out of 394 genes, 15.2% | 1095 out of 15514 genes, 7.1% | 7.17E-06 |
| 9 | pectin catabolic process (view genes) | 11 out of 394 genes, 2.8% | 52 out of 15514 genes, 0.3% | 3.66E-05 |
| 10 | translation (view genes) | 68 out of 394 genes, 17.3% | 1410 out of 15514 genes, 9.1% | 0.0001 |
| 11 | peptide metabolic process (view genes) | 70 out of 394 genes, 17.8% | 1469 out of 15514 genes, 9.5% | 0.0001 |
| 12 | peptide biosynthetic process (view genes) | 68 out of 394 genes, 17.3% | 1413 out of 15514 genes, 9.1% | 0.00011 |
| 13 | response to biotic stimulus (view genes) | 18 out of 394 genes, 4.6% | 164 out of 15514 genes, 1.1% | 0.00012 |
| 14 | response to oxidative stress (view genes) | 21 out of 394 genes, 5.3% | 231 out of 15514 genes, 1.5% | 0.00029 |
| 15 | amide biosynthetic process (view genes) | 68 out of 394 genes, 17.3% | 1461 out of 15514 genes, 9.4% | 0.00037 |
| 16 | cellular amide metabolic process (view genes) | 70 out of 394 genes, 17.8% | 1547 out of 15514 genes, 10.0% | 0.0007 |
| 17 | organ morphogenesis (view genes) | 8 out of 394 genes, 2.0% | 37 out of 15514 genes, 0.2% | 0.00204 |
| 18 | response to external biotic stimulus (view genes) | 14 out of 394 genes, 3.6% | 125 out of 15514 genes, 0.8% | 0.00219 |
| 19 | response to other organism (view genes) | 14 out of 394 genes, 3.6% | 125 out of 15514 genes, 0.8% | 0.00219 |
| 20 | single-organism catabolic process (view genes) | 33 out of 394 genes, 8.4% | 545 out of 15514 genes, 3.5% | 0.00231 |
| 21 | response to fungus (view genes) | 8 out of 394 genes, 2.0% | 38 out of 15514 genes, 0.2% | 0.00253 |
| 22 | response to stimulus (view genes) | 78 out of 394 genes, 19.8% | 1875 out of 15514 genes, 12.1% | 0.00391 |
| 23 | response to stress (view genes) | 46 out of 394 genes, 11.7% | 930 out of 15514 genes, 6.0% | 0.00689 |
| 24 | animal organ development (view genes) | 10 out of 394 genes, 2.5% | 75 out of 15514 genes, 0.5% | 0.01188 |
| 25 | polysaccharide catabolic process (view genes) | 13 out of 394 genes, 3.3% | 131 out of 15514 genes, 0.8% | 0.01818 |
| 26 | defense response (view genes) | 17 out of 394 genes, 4.3% | 214 out of 15514 genes, 1.4% | 0.02133 |
| 27 | phosphate ion transport (view genes) | 6 out of 394 genes, 1.5% | 26 out of 15514 genes, 0.2% | 0.02422 |
| 28 | paclitaxel metabolic process (view genes) | 5 out of 394 genes, 1.3% | 17 out of 15514 genes, 0.1% | 0.03105 |
| 29 | paclitaxel biosynthetic process (view genes) | 5 out of 394 genes, 1.3% | 17 out of 15514 genes, 0.1% | 0.03105 |
| 30 | monovalent inorganic anion homeostasis (view genes) | 5 out of 394 genes, 1.3% | 18 out of 15514 genes, 0.1% | 0.0421 |
| 31 | galacturonan metabolic process (view genes) | 11 out of 394 genes, 2.8% | 106 out of 15514 genes, 0.7% | 0.04949 |
| 32 | pectin metabolic process (view genes) | 11 out of 394 genes, 2.8% | 106 out of 15514 genes, 0.7% | 0.04949 |
|  |  | **Terms for S2-VS-S3._Molecular Function** |  |  |
|  | **Gene Ontology term** | **Cluster frequency** | **Genome frequency of use** | **Corrected P-value** |
|  |  |  |  |  |
| 1 | peroxiredoxin activity (view genes) | 2 out of 39 genes, 5.1% | 16 out of 18328 genes, 0.1% | 0.03481 |
| 2 | transmembrane signaling receptor activity (view genes) | 2 out of 39 genes, 5.1% | 30 out of 18328 genes, 0.2% | 0.12383 |
| 3 | transmembrane receptor activity (view genes) | 2 out of 39 genes, 5.1% | 34 out of 18328 genes, 0.2% | 0.15885 |
| 4 | chitin binding (view genes) | 2 out of 39 genes, 5.1% | 39 out of 18328 genes, 0.2% | 0.20842 |
| 5 | chitinase activity (view genes) | 2 out of 39 genes, 5.1% | 55 out of 18328 genes, 0.3% | 0.40883 |
|  |  | **Terms for S2-VS-S3._Biological Process** |  |  |
|  | **Gene Ontology term** | **Cluster frequency** | **Genome frequency of use** | **Corrected P-value** |
| 1 | cellular homeostasis (view genes) | 5 out of 37 genes, 13.5% | 246 out of 15514 genes, 1.6% | 0.03464 |
| 2 | cellular chemical homeostasis (view genes) | 4 out of 37 genes, 10.8% | 143 out of 15514 genes, 0.9% | 0.04511 |
| 3 | cellular metal ion homeostasis (view genes) | 3 out of 37 genes, 8.1% | 71 out of 15514 genes, 0.5% | 0.07979 |
| 4 | homeostatic process (view genes) | 5 out of 37 genes, 13.5% | 317 out of 15514 genes, 2.0% | 0.10994 |
| 5 | metal ion homeostasis (view genes) | 3 out of 37 genes, 8.1% | 81 out of 15514 genes, 0.5% | 0.11717 |
| 6 | chemical homeostasis (view genes) | 4 out of 37 genes, 10.8% | 202 out of 15514 genes, 1.3% | 0.16453 |
| 7 | cellular cation homeostasis (view genes) | 3 out of 37 genes, 8.1% | 95 out of 15514 genes, 0.6% | 0.18578 |
| 8 | cell wall macromolecule catabolic process (view genes) | 2 out of 37 genes, 5.4% | 25 out of 15514 genes, 0.2% | 0.20049 |
| 9 | cation homeostasis (view genes) | 3 out of 37 genes, 8.1% | 109 out of 15514 genes, 0.7% | 0.2754 |
| 10 | cellular ion homeostasis (view genes) | 3 out of 37 genes, 8.1% | 111 out of 15514 genes, 0.7% | 0.29004 |
| 11 | inorganic ion homeostasis (view genes) | 3 out of 37 genes, 8.1% | 127 out of 15514 genes, 0.8% | 0.42467 |
| 12 | ion homeostasis (view genes) | 3 out of 37 genes, 8.1% | 133 out of 15514 genes, 0.9% | 0.48351 |
