# Supplementary material for: An Integrated Transcriptome and Proteome Analysis Reveals Putative Regulators of Adventitious Root Formation in Taxodium ‘Zhongshanshan’
Source: Int J Mol Sci. 2019 Mar 11;20(5):1225. doi: 10.3390/ijms20051225 (PMC6429173; doi:10.3390/ijms20051225)
Supplement: Supplementary file 1 [file ijms-20-01225-s001.zip › Supplementary material20190227/Table S3.docx]

**Table S3** The result of KEGG pathway classification and functional enrichment of DEGs

|  |  |  |  | S0-VS-S1 |  |  |  |  |
| --- | --- | --- | --- | --- | --- | --- | --- | --- |
|  | **Pathway** | **DEGs genes with pathway annotation (1462)** | **All genes with pathway annotation (53682)** | **Pvalue** | **Qvalue** | **Pathway ID** | **Level 1** | **Level 2** |
| 1 | [Glycolysis / Gluconeogenesis](file:///F:\转录组蛋白组\图表\整理图\新建%20Microsoft%20Office%20Excel%20工作表%20(2).xlsx#RANGE!gene1) | 78 (5.34%) | 730 (1.36%) | 1.39E-24 | 1.65E-22 | ko00010 | Metabolism | Carbohydrate metabolism |
| 2 | [Biosynthesis of secondary metabolites](file:///F:\转录组蛋白组\图表\整理图\新建%20Microsoft%20Office%20Excel%20工作表%20(2).xlsx#RANGE!gene2) | 312 (21.34%) | 7080 (13.19%) | 1.90E-18 | 1.13E-16 | ko01110 | Metabolism | Global and overview maps |
| 3 | [Taurine and hypotaurine metabolism](file:///F:\转录组蛋白组\图表\整理图\新建%20Microsoft%20Office%20Excel%20工作表%20(2).xlsx#RANGE!gene3) | 22 (1.5%) | 79 (0.15%) | 1.43E-16 | 5.68E-15 | ko00430 | Metabolism | Metabolism of other amino acids |
| 4 | [Metabolic pathways](file:///F:\转录组蛋白组\图表\整理图\新建%20Microsoft%20Office%20Excel%20工作表%20(2).xlsx#RANGE!gene4) | 435 (29.75%) | 11808 (22%) | 1.46E-12 | 4.33E-11 | ko01100 | Metabolism | Global and overview maps |
| 5 | [Phenylpropanoid biosynthesis](file:///F:\转录组蛋白组\图表\整理图\新建%20Microsoft%20Office%20Excel%20工作表%20(2).xlsx#RANGE!gene5) | 81 (5.54%) | 1456 (2.71%) | 1.68E-09 | 4.00E-08 | ko00940 | Metabolism | Biosynthesis of other secondary metabolites |
| 6 | [Carbon fixation in photosynthetic organisms](file:///F:\转录组蛋白组\图表\整理图\新建%20Microsoft%20Office%20Excel%20工作表%20(2).xlsx#RANGE!gene6) | 35 (2.39%) | 508 (0.95%) | 7.16E-07 | 1.42E-05 | ko00710 | Metabolism | Energy metabolism |
| 7 | [Alanine, aspartate and glutamate metabolism](file:///F:\转录组蛋白组\图表\整理图\新建%20Microsoft%20Office%20Excel%20工作表%20(2).xlsx#RANGE!gene7) | 26 (1.78%) | 328 (0.61%) | 1.49E-06 | 2.50E-05 | ko00250 | Metabolism | Amino acid metabolism |
| 8 | [alpha-Linolenic acid metabolism](file:///F:\转录组蛋白组\图表\整理图\新建%20Microsoft%20Office%20Excel%20工作表%20(2).xlsx#RANGE!gene8) | 27 (1.85%) | 351 (0.65%) | 1.68E-06 | 2.50E-05 | ko00592 | Metabolism | Lipid metabolism |
| 9 | [Degradation of aromatic compounds](file:///F:\转录组蛋白组\图表\整理图\新建%20Microsoft%20Office%20Excel%20工作表%20(2).xlsx#RANGE!gene9) | 16 (1.09%) | 148 (0.28%) | 3.17E-06 | 4.19E-05 | ko01220 | Metabolism | Global and overview maps |
| 10 | [Terpenoid backbone biosynthesis](file:///F:\转录组蛋白组\图表\整理图\新建%20Microsoft%20Office%20Excel%20工作表%20(2).xlsx#RANGE!gene10) | 28 (1.92%) | 400 (0.75%) | 6.62E-06 | 7.87E-05 | ko00900 | Metabolism | Metabolism of terpenoids and polyketides |
| 11 | [Photosynthesis - antenna proteins](file:///F:\转录组蛋白组\图表\整理图\新建%20Microsoft%20Office%20Excel%20工作表%20(2).xlsx#RANGE!gene11) | 8 (0.55%) | 45 (0.08%) | 2.61E-05 | 2.83E-04 | ko00196 | Metabolism | Energy metabolism |
| 12 | [Tyrosine metabolism](file:///F:\转录组蛋白组\图表\整理图\新建%20Microsoft%20Office%20Excel%20工作表%20(2).xlsx#RANGE!gene12) | 28 (1.92%) | 482 (0.9%) | 0.000174484 | 1.62E-03 | ko00350 | Metabolism | Amino acid metabolism |
| 13 | [Carotenoid biosynthesis](file:///F:\转录组蛋白组\图表\整理图\新建%20Microsoft%20Office%20Excel%20工作表%20(2).xlsx#RANGE!gene13) | 18 (1.23%) | 248 (0.46%) | 0.000177451 | 1.62E-03 | ko00906 | Metabolism | Metabolism of terpenoids and polyketides |
| 14 | [beta-Alanine metabolism](file:///F:\转录组蛋白组\图表\整理图\新建%20Microsoft%20Office%20Excel%20工作表%20(2).xlsx#RANGE!gene14) | 28 (1.92%) | 493 (0.92%) | 0.000251799 | 2.14E-03 | ko00410 | Metabolism | Metabolism of other amino acids |
| 15 | [Flavonoid biosynthesis](file:///F:\转录组蛋白组\图表\整理图\新建%20Microsoft%20Office%20Excel%20工作表%20(2).xlsx#RANGE!gene15) | 30 (2.05%) | 557 (1.04%) | 0.000377921 | 3.00E-03 | ko00941 | Metabolism | Biosynthesis of other secondary metabolites |
| 16 | [Biosynthesis of amino acids](file:///F:\转录组蛋白组\图表\整理图\新建%20Microsoft%20Office%20Excel%20工作表%20(2).xlsx#RANGE!gene16) | 56 (3.83%) | 1278 (2.38%) | 0.00040465 | 3.01E-03 | ko01230 | Metabolism | Global and overview maps |
| 17 | [Ubiquinone and other terpenoid-quinone biosynthesis](file:///F:\转录组蛋白组\图表\整理图\新建%20Microsoft%20Office%20Excel%20工作表%20(2).xlsx#RANGE!gene17) | 15 (1.03%) | 210 (0.39%) | 0.000700135 | 4.90E-03 | ko00130 | Metabolism | Metabolism of cofactors and vitamins |
| 18 | [Butanoate metabolism](file:///F:\转录组蛋白组\图表\整理图\新建%20Microsoft%20Office%20Excel%20工作表%20(2).xlsx#RANGE!gene18) | 12 (0.82%) | 150 (0.28%) | 0.000875266 | 5.79E-03 | ko00650 | Metabolism | Carbohydrate metabolism |
| 19 | [Fatty acid degradation](file:///F:\转录组蛋白组\图表\整理图\新建%20Microsoft%20Office%20Excel%20工作表%20(2).xlsx#RANGE!gene19) | 16 (1.09%) | 239 (0.45%) | 0.000947652 | 5.94E-03 | ko00071 | Metabolism | Lipid metabolism |
| 20 | [Phenylalanine metabolism](file:///F:\转录组蛋白组\图表\整理图\新建%20Microsoft%20Office%20Excel%20工作表%20(2).xlsx#RANGE!gene20) | 26 (1.78%) | 488 (0.91%) | 0.001044041 | 6.21E-03 | ko00360 | Metabolism | Amino acid metabolism |
| 21 | [Linoleic acid metabolism](file:///F:\转录组蛋白组\图表\整理图\新建%20Microsoft%20Office%20Excel%20工作表%20(2).xlsx#RANGE!gene21) | 17 (1.16%) | 267 (0.5%) | 0.001159465 | 6.57E-03 | ko00591 | Metabolism | Lipid metabolism |
| 22 | [Pentose phosphate pathway](file:///F:\转录组蛋白组\图表\整理图\新建%20Microsoft%20Office%20Excel%20工作表%20(2).xlsx#RANGE!gene22) | 20 (1.37%) | 355 (0.66%) | 0.001978607 | 1.07E-02 | ko00030 | Metabolism | Carbohydrate metabolism |
| 23 | [Stilbenoid, diarylheptanoid and gingerol biosynthesis](file:///F:\转录组蛋白组\图表\整理图\新建%20Microsoft%20Office%20Excel%20工作表%20(2).xlsx#RANGE!gene23) | 16 (1.09%) | 272 (0.51%) | 0.003505534 | 1.81E-02 | ko00945 | Metabolism | Biosynthesis of other secondary metabolites |
| 24 | [Carbon metabolism](file:///F:\转录组蛋白组\图表\整理图\新建%20Microsoft%20Office%20Excel%20工作表%20(2).xlsx#RANGE!gene24) | 53 (3.63%) | 1348 (2.51%) | 0.00547072 | 2.71E-02 | ko01200 | Metabolism | Global and overview maps |
| 25 | [Fructose and mannose metabolism](file:///F:\转录组蛋白组\图表\整理图\新建%20Microsoft%20Office%20Excel%20工作表%20(2).xlsx#RANGE!gene25) | 20 (1.37%) | 405 (0.75%) | 0.008389539 | 3.99E-02 | ko00051 | Metabolism | Carbohydrate metabolism |
| 26 | [Arginine and proline metabolism](file:///F:\转录组蛋白组\图表\整理图\新建%20Microsoft%20Office%20Excel%20工作表%20(2).xlsx#RANGE!gene26) | 15 (1.03%) | 293 (0.55%) | 0.01530204 | 7.00E-02 | ko00330 | Metabolism | Amino acid metabolism |
| 27 | [Cysteine and methionine metabolism](file:///F:\转录组蛋白组\图表\整理图\新建%20Microsoft%20Office%20Excel%20工作表%20(2).xlsx#RANGE!gene27) | 23 (1.57%) | 519 (0.97%) | 0.01654791 | 7.29E-02 | ko00270 | Metabolism | Amino acid metabolism |
| 28 | [Galactose metabolism](file:///F:\转录组蛋白组\图表\整理图\新建%20Microsoft%20Office%20Excel%20工作表%20(2).xlsx#RANGE!gene28) | 27 (1.85%) | 663 (1.24%) | 0.02656535 | 1.13E-01 | ko00052 | Metabolism | Carbohydrate metabolism |
| 29 | [Ascorbate and aldarate metabolism](file:///F:\转录组蛋白组\图表\整理图\新建%20Microsoft%20Office%20Excel%20工作表%20(2).xlsx#RANGE!gene29) | 19 (1.3%) | 449 (0.84%) | 0.04066359 | 1.67E-01 | ko00053 | Metabolism | Carbohydrate metabolism |
|  |  |  |  | **S1-VS-S2** |  |  |  |  |
|  |  |  |  |  |  |  |  |  |
|  | **Pathway** | **DEGs genes with pathway annotation (1515)** | **All genes with pathway annotation (53682)** | **Pvalue** | **Qvalue** | **Pathway ID** | **Level 1** | **Level 2** |
| 1 | [Biosynthesis of secondary metabolites](file:///F:\转录组蛋白组\图表\整理图\新建%20Microsoft%20Office%20Excel%20工作表%20(2).xlsx#RANGE!gene1) | 347 (22.9%) | 7080 (13.19%) | 8.46E-26 | 9.47E-24 | ko01110 | Metabolism | Global and overview maps |
| 2 | [Ribosome](file:///F:\转录组蛋白组\图表\整理图\新建%20Microsoft%20Office%20Excel%20工作表%20(2).xlsx#RANGE!gene2) | 127 (8.38%) | 1677 (3.12%) | 1.18E-23 | 6.62E-22 | ko03010 | Genetic Information Processing | Translation |
| 3 | [Metabolic pathways](file:///F:\转录组蛋白组\图表\整理图\新建%20Microsoft%20Office%20Excel%20工作表%20(2).xlsx#RANGE!gene3) | 480 (31.68%) | 11808 (22%) | 5.64E-19 | 2.11E-17 | ko01100 | Metabolism | Global and overview maps |
| 4 | [Phenylpropanoid biosynthesis](file:///F:\转录组蛋白组\图表\整理图\新建%20Microsoft%20Office%20Excel%20工作表%20(2).xlsx#RANGE!gene4) | 104 (6.86%) | 1456 (2.71%) | 9.29E-18 | 2.60E-16 | ko00940 | Metabolism | Biosynthesis of other secondary metabolites |
| 5 | [Proteasome](file:///F:\转录组蛋白组\图表\整理图\新建%20Microsoft%20Office%20Excel%20工作表%20(2).xlsx#RANGE!gene5) | 30 (1.98%) | 237 (0.44%) | 8.81E-12 | 1.97E-10 | ko03050 | Genetic Information Processing | Folding, sorting and degradation |
| 6 | [Linoleic acid metabolism](file:///F:\转录组蛋白组\图表\整理图\新建%20Microsoft%20Office%20Excel%20工作表%20(2).xlsx#RANGE!gene6) | 31 (2.05%) | 267 (0.5%) | 3.78E-11 | 7.06E-10 | ko00591 | Metabolism | Lipid metabolism |
| 7 | [Tryptophan metabolism](file:///F:\转录组蛋白组\图表\整理图\新建%20Microsoft%20Office%20Excel%20工作表%20(2).xlsx#RANGE!gene7) | 37 (2.44%) | 375 (0.7%) | 6.29E-11 | 1.01E-09 | ko00380 | Metabolism | Amino acid metabolism |
| 8 | [Caffeine metabolism](file:///F:\转录组蛋白组\图表\整理图\新建%20Microsoft%20Office%20Excel%20工作表%20(2).xlsx#RANGE!gene8) | 19 (1.25%) | 110 (0.2%) | 2.75E-10 | 3.86E-09 | ko00232 | Metabolism | Biosynthesis of other secondary metabolites |
| 9 | [Diterpenoid biosynthesis](file:///F:\转录组蛋白组\图表\整理图\新建%20Microsoft%20Office%20Excel%20工作表%20(2).xlsx#RANGE!gene9) | 28 (1.85%) | 285 (0.53%) | 1.40E-08 | 1.75E-07 | ko00904 | Metabolism | Metabolism of terpenoids and polyketides |
| 10 | [Pentose and glucuronate interconversions](file:///F:\转录组蛋白组\图表\整理图\新建%20Microsoft%20Office%20Excel%20工作表%20(2).xlsx#RANGE!gene10) | 45 (2.97%) | 658 (1.23%) | 6.83E-08 | 7.65E-07 | ko00040 | Metabolism | Carbohydrate metabolism |
| 11 | [Isoquinoline alkaloid biosynthesis](file:///F:\转录组蛋白组\图表\整理图\新建%20Microsoft%20Office%20Excel%20工作表%20(2).xlsx#RANGE!gene11) | 28 (1.85%) | 355 (0.66%) | 1.32E-06 | 1.34E-05 | ko00950 | Metabolism | Biosynthesis of other secondary metabolites |
| 12 | [Flavonoid biosynthesis](file:///F:\转录组蛋白组\图表\整理图\新建%20Microsoft%20Office%20Excel%20工作表%20(2).xlsx#RANGE!gene12) | 37 (2.44%) | 557 (1.04%) | 1.91E-06 | 1.79E-05 | ko00941 | Metabolism | Biosynthesis of other secondary metabolites |
| 13 | [Amino sugar and nucleotide sugar metabolism](file:///F:\转录组蛋白组\图表\整理图\新建%20Microsoft%20Office%20Excel%20工作表%20(2).xlsx#RANGE!gene13) | 56 (3.7%) | 1027 (1.91%) | 2.97E-06 | 2.56E-05 | ko00520 | Metabolism | Carbohydrate metabolism |
| 14 | [Flavone and flavonol biosynthesis](file:///F:\转录组蛋白组\图表\整理图\新建%20Microsoft%20Office%20Excel%20工作表%20(2).xlsx#RANGE!gene14) | 20 (1.32%) | 235 (0.44%) | 1.37E-05 | 1.10E-04 | ko00944 | Metabolism | Biosynthesis of other secondary metabolites |
| 15 | [Tyrosine metabolism](file:///F:\转录组蛋白组\图表\整理图\新建%20Microsoft%20Office%20Excel%20工作表%20(2).xlsx#RANGE!gene15) | 31 (2.05%) | 482 (0.9%) | 2.35E-05 | 1.75E-04 | ko00350 | Metabolism | Amino acid metabolism |
| 16 | [Ascorbate and aldarate metabolism](file:///F:\转录组蛋白组\图表\整理图\新建%20Microsoft%20Office%20Excel%20工作表%20(2).xlsx#RANGE!gene16) | 29 (1.91%) | 449 (0.84%) | 3.93E-05 | 2.75E-04 | ko00053 | Metabolism | Carbohydrate metabolism |
| 17 | [Terpenoid backbone biosynthesis](file:///F:\转录组蛋白组\图表\整理图\新建%20Microsoft%20Office%20Excel%20工作表%20(2).xlsx#RANGE!gene17) | 26 (1.72%) | 400 (0.75%) | 8.67E-05 | 5.71E-04 | ko00900 | Metabolism | Metabolism of terpenoids and polyketides |
| 18 | [Tropane, piperidine and pyridine alkaloid biosynthesis](file:///F:\转录组蛋白组\图表\整理图\新建%20Microsoft%20Office%20Excel%20工作表%20(2).xlsx#RANGE!gene18) | 23 (1.52%) | 342 (0.64%) | 0.000131386 | 8.18E-04 | ko00960 | Metabolism | Biosynthesis of other secondary metabolites |
| 19 | [Limonene and pinene degradation](file:///F:\转录组蛋白组\图表\整理图\新建%20Microsoft%20Office%20Excel%20工作表%20(2).xlsx#RANGE!gene19) | 16 (1.06%) | 198 (0.37%) | 0.000175385 | 1.03E-03 | ko00903 | Metabolism | Metabolism of terpenoids and polyketides |
| 20 | [Carotenoid biosynthesis](file:///F:\转录组蛋白组\图表\整理图\新建%20Microsoft%20Office%20Excel%20工作表%20(2).xlsx#RANGE!gene20) | 18 (1.19%) | 248 (0.46%) | 0.000272388 | 1.53E-03 | ko00906 | Metabolism | Metabolism of terpenoids and polyketides |
| 21 | [Phenylalanine metabolism](file:///F:\转录组蛋白组\图表\整理图\新建%20Microsoft%20Office%20Excel%20工作表%20(2).xlsx#RANGE!gene21) | 28 (1.85%) | 488 (0.91%) | 0.000375601 | 2.00E-03 | ko00360 | Metabolism | Amino acid metabolism |
| 22 | [Zeatin biosynthesis](file:///F:\转录组蛋白组\图表\整理图\新建%20Microsoft%20Office%20Excel%20工作表%20(2).xlsx#RANGE!gene22) | 18 (1.19%) | 258 (0.48%) | 0.000437722 | 2.23E-03 | ko00908 | Metabolism | Metabolism of terpenoids and polyketides |
| 23 | [Endocytosis](file:///F:\转录组蛋白组\图表\整理图\新建%20Microsoft%20Office%20Excel%20工作表%20(2).xlsx#RANGE!gene23) | 97 (6.4%) | 2434 (4.53%) | 0.000460661 | 2.24E-03 | ko04144 | Cellular Processes | Transport and catabolism |
| 24 | [beta-Alanine metabolism](file:///F:\转录组蛋白组\图表\整理图\新建%20Microsoft%20Office%20Excel%20工作表%20(2).xlsx#RANGE!gene24) | 27 (1.78%) | 493 (0.92%) | 0.00094827 | 4.43E-03 | ko00410 | Metabolism | Metabolism of other amino acids |
| 25 | [Photosynthesis - antenna proteins](file:///F:\转录组蛋白组\图表\整理图\新建%20Microsoft%20Office%20Excel%20工作表%20(2).xlsx#RANGE!gene25) | 6 (0.4%) | 45 (0.08%) | 0.001589554 | 7.12E-03 | ko00196 | Metabolism | Energy metabolism |
| 26 | [Stilbenoid, diarylheptanoid and gingerol biosynthesis](file:///F:\转录组蛋白组\图表\整理图\新建%20Microsoft%20Office%20Excel%20工作表%20(2).xlsx#RANGE!gene26) | 17 (1.12%) | 272 (0.51%) | 0.002052455 | 8.84E-03 | ko00945 | Metabolism | Biosynthesis of other secondary metabolites |
| 27 | [Cyanoamino acid metabolism](file:///F:\转录组蛋白组\图表\整理图\新建%20Microsoft%20Office%20Excel%20工作表%20(2).xlsx#RANGE!gene27) | 26 (1.72%) | 499 (0.93%) | 0.002317012 | 9.61E-03 | ko00460 | Metabolism | Metabolism of other amino acids |
| 28 | [Vitamin B6 metabolism](file:///F:\转录组蛋白组\图表\整理图\新建%20Microsoft%20Office%20Excel%20工作表%20(2).xlsx#RANGE!gene28) | 8 (0.53%) | 97 (0.18%) | 0.006236464 | 2.49E-02 | ko00750 | Metabolism | Metabolism of cofactors and vitamins |
| 29 | [Brassinosteroid biosynthesis](file:///F:\转录组蛋白组\图表\整理图\新建%20Microsoft%20Office%20Excel%20工作表%20(2).xlsx#RANGE!gene29) | 8 (0.53%) | 98 (0.18%) | 0.006626917 | 2.56E-02 | ko00905 | Metabolism | Metabolism of terpenoids and polyketides |
| 30 | [Fructose and mannose metabolism](file:///F:\转录组蛋白组\图表\整理图\新建%20Microsoft%20Office%20Excel%20工作表%20(2).xlsx#RANGE!gene30) | 20 (1.32%) | 405 (0.75%) | 0.01200869 | 4.48E-02 | ko00051 | Metabolism | Carbohydrate metabolism |
| 31 | [Glycine, serine and threonine metabolism](file:///F:\转录组蛋白组\图表\整理图\新建%20Microsoft%20Office%20Excel%20工作表%20(2).xlsx#RANGE!gene31) | 27 (1.78%) | 608 (1.13%) | 0.01497434 | 5.41E-02 | ko00260 | Metabolism | Amino acid metabolism |
| 32 | [Porphyrin and chlorophyll metabolism](file:///F:\转录组蛋白组\图表\整理图\新建%20Microsoft%20Office%20Excel%20工作表%20(2).xlsx#RANGE!gene32) | 14 (0.92%) | 279 (0.52%) | 0.02826289 | 9.89E-02 | ko00860 | Metabolism | Metabolism of cofactors and vitamins |
| 33 | [Photosynthesis](file:///F:\转录组蛋白组\图表\整理图\新建%20Microsoft%20Office%20Excel%20工作表%20(2).xlsx#RANGE!gene33) | 8 (0.53%) | 136 (0.25%) | 0.03951594 | 1.34E-01 | ko00195 | Metabolism | Energy metabolism |
|  |  |  |  | **S2-VS-S3** |  |  |  |  |
|  | **Pathway** | **DEGs genes with pathway annotation (124)** | **All genes with pathway annotation (53682)** | **Pvalue** | **Qvalue** | **Pathway ID** | **Level 1** | **Level 2** |
| 1 | [Metabolic pathways](file:///F:\转录组蛋白组\图表\整理图\新建%20Microsoft%20Office%20Excel%20工作表%20(2).xlsx#RANGE!gene1) | 44 (35.48%) | 11808 (22%) | 0.000411075 | 0.02088593 | ko01100 | Metabolism | Global and overview maps |
| 2 | [Amino sugar and nucleotide sugar metabolism](file:///F:\转录组蛋白组\图表\整理图\新建%20Microsoft%20Office%20Excel%20工作表%20(2).xlsx#RANGE!gene2) | 9 (7.26%) | 1027 (1.91%) | 0.000663045 | 0.02088593 | ko00520 | Metabolism | Carbohydrate metabolism |
| 3 | [Selenocompound metabolism](file:///F:\转录组蛋白组\图表\整理图\新建%20Microsoft%20Office%20Excel%20工作表%20(2).xlsx#RANGE!gene3) | 3 (2.42%) | 133 (0.25%) | 0.003705601 | 0.07733606 | ko00450 | Metabolism | Metabolism of other amino acids |
| 4 | [Photosynthesis - antenna proteins](file:///F:\转录组蛋白组\图表\整理图\新建%20Microsoft%20Office%20Excel%20工作表%20(2).xlsx#RANGE!gene4) | 2 (1.61%) | 45 (0.08%) | 0.004910226 | 0.07733606 | ko00196 | Metabolism | Energy metabolism |
| 5 | [Carbon fixation in photosynthetic organisms](file:///F:\转录组蛋白组\图表\整理图\新建%20Microsoft%20Office%20Excel%20工作表%20(2).xlsx#RANGE!gene5) | 5 (4.03%) | 508 (0.95%) | 0.006673671 | 0.08408825 | ko00710 | Metabolism | Energy metabolism |
| 6 | [Thiamine metabolism](file:///F:\转录组蛋白组\图表\整理图\新建%20Microsoft%20Office%20Excel%20工作表%20(2).xlsx#RANGE!gene6) | 2 (1.61%) | 71 (0.13%) | 0.01185332 | 0.12445986 | ko00730 | Metabolism | Metabolism of cofactors and vitamins |
| 7 | [Nicotinate and nicotinamide metabolism](file:///F:\转录组蛋白组\图表\整理图\新建%20Microsoft%20Office%20Excel%20工作表%20(2).xlsx#RANGE!gene7) | 2 (1.61%) | 111 (0.21%) | 0.02743314 | 0.24689826 | ko00760 | Metabolism | Metabolism of cofactors and vitamins |
| 8 | [Inositol phosphate metabolism](file:///F:\转录组蛋白组\图表\整理图\新建%20Microsoft%20Office%20Excel%20工作表%20(2).xlsx#RANGE!gene8) | 3 (2.42%) | 347 (0.65%) | 0.04688312 | 0.33094587 | ko00562 | Metabolism | Carbohydrate metabolism |
| 9 | Isoquinoline alkaloid biosynthesis | 3 (2.42%) | 355 (0.66%) | 0.04956075 | 0.33094587 | ko00950 | Metabolism | Biosynthesis of other secondary metabolites |
