# Supplementary material for: An Integrated Transcriptome and Proteome Analysis Reveals Putative Regulators of Adventitious Root Formation in Taxodium ‘Zhongshanshan’
Source: Int J Mol Sci. 2019 Mar 11;20(5):1225. doi: 10.3390/ijms20051225 (PMC6429173; doi:10.3390/ijms20051225)
Supplement: Supplementary file 1 [file ijms-20-01225-s001.zip › Supplementary material20190227/Table S4.docx]

**Table S4** Overview of protein identification

| Sample name | Total spectra | Spectra | Unique Spetra | Peptide | Unique Peptide | Protein |
| --- | --- | --- | --- | --- | --- | --- |
| Taxodiaceae | 330831 | 64377 | 54983 | 25660 | 23032 | 7356 |
