# Supplementary material for: An Integrated Transcriptome and Proteome Analysis Reveals Putative Regulators of Adventitious Root Formation in Taxodium ‘Zhongshanshan’
Source: Int J Mol Sci. 2019 Mar 11;20(5):1225. doi: 10.3390/ijms20051225 (PMC6429173; doi:10.3390/ijms20051225)
Supplement: Supplementary file 1 [file ijms-20-01225-s001.zip › Supplementary material20190227/Table S6.docx]

**Table S6** The result of pathway annotation of DEPs

|  | Pathway | DEPs with pathway annotation (6017) | Pathway ID |
| --- | --- | --- | --- |
| 1 | Metabolic pathways | 1780 (29.58%) | ko01100 |
| 2 | [Biosynthesis of secondary metabolites](file:///F:\转录组蛋白组\图表\整理图\新建%20Microsoft%20Office%20Excel%20工作表%20(2).xlsx#RANGE!gene2) | 1131 (18.8%) | ko01110 |
| 3 | [Carbon metabolism](file:///F:\转录组蛋白组\图表\整理图\新建%20Microsoft%20Office%20Excel%20工作表%20(2).xlsx#RANGE!gene3) | 237 (3.94%) | ko01200 |
| 4 | [Ribosome](file:///F:\转录组蛋白组\图表\整理图\新建%20Microsoft%20Office%20Excel%20工作表%20(2).xlsx#RANGE!gene4) | 227 (3.77%) | ko03010 |
| 5 | [Phenylpropanoid biosynthesis](file:///F:\转录组蛋白组\图表\整理图\新建%20Microsoft%20Office%20Excel%20工作表%20(2).xlsx#RANGE!gene5) | 211 (3.51%) | ko00940 |
| 6 | [Biosynthesis of amino acids](file:///F:\转录组蛋白组\图表\整理图\新建%20Microsoft%20Office%20Excel%20工作表%20(2).xlsx#RANGE!gene6) | 206 (3.42%) | ko01230 |
| 7 | [Starch and sucrose metabolism](file:///F:\转录组蛋白组\图表\整理图\新建%20Microsoft%20Office%20Excel%20工作表%20(2).xlsx#RANGE!gene7) | 189 (3.14%) | ko00500 |
| 8 | [Spliceosome](file:///F:\转录组蛋白组\图表\整理图\新建%20Microsoft%20Office%20Excel%20工作表%20(2).xlsx#RANGE!gene8) | 187 (3.11%) | ko03040 |
| 9 | [Endocytosis](file:///F:\转录组蛋白组\图表\整理图\新建%20Microsoft%20Office%20Excel%20工作表%20(2).xlsx#RANGE!gene9) | 186 (3.09%) | ko04144 |
| 10 | [Plant-pathogen interaction](file:///F:\转录组蛋白组\图表\整理图\新建%20Microsoft%20Office%20Excel%20工作表%20(2).xlsx#RANGE!gene10) | 186 (3.09%) | ko04626 |
| 11 | [RNA transport](file:///F:\转录组蛋白组\图表\整理图\新建%20Microsoft%20Office%20Excel%20工作表%20(2).xlsx#RANGE!gene11) | 177 (2.94%) | ko03013 |
| 12 | [Protein processing in endoplasmic reticulum](file:///F:\转录组蛋白组\图表\整理图\新建%20Microsoft%20Office%20Excel%20工作表%20(2).xlsx#RANGE!gene12) | 172 (2.86%) | ko04141 |
| 13 | [Amino sugar and nucleotide sugar metabolism](file:///F:\转录组蛋白组\图表\整理图\新建%20Microsoft%20Office%20Excel%20工作表%20(2).xlsx#RANGE!gene13) | 151 (2.51%) | ko00520 |
| 14 | [Glycolysis / Gluconeogenesis](file:///F:\转录组蛋白组\图表\整理图\新建%20Microsoft%20Office%20Excel%20工作表%20(2).xlsx#RANGE!gene14) | 120 (1.99%) | ko00010 |
| 15 | [mRNA surveillance pathway](file:///F:\转录组蛋白组\图表\整理图\新建%20Microsoft%20Office%20Excel%20工作表%20(2).xlsx#RANGE!gene15) | 114 (1.89%) | ko03015 |
| 16 | [Peroxisome](file:///F:\转录组蛋白组\图表\整理图\新建%20Microsoft%20Office%20Excel%20工作表%20(2).xlsx#RANGE!gene16) | 110 (1.83%) | ko04146 |
| 17 | [Purine metabolism](file:///F:\转录组蛋白组\图表\整理图\新建%20Microsoft%20Office%20Excel%20工作表%20(2).xlsx#RANGE!gene17) | 104 (1.73%) | ko00230 |
| 18 | [Glycerolipid metabolism](file:///F:\转录组蛋白组\图表\整理图\新建%20Microsoft%20Office%20Excel%20工作表%20(2).xlsx#RANGE!gene18) | 103 (1.71%) | ko00561 |
| 19 | [Pyruvate metabolism](file:///F:\转录组蛋白组\图表\整理图\新建%20Microsoft%20Office%20Excel%20工作表%20(2).xlsx#RANGE!gene19) | 95 (1.58%) | ko00620 |
| 20 | [Glycine, serine and threonine metabolism](file:///F:\转录组蛋白组\图表\整理图\新建%20Microsoft%20Office%20Excel%20工作表%20(2).xlsx#RANGE!gene20) | 90 (1.5%) | ko00260 |
| 21 | [RNA degradation](file:///F:\转录组蛋白组\图表\整理图\新建%20Microsoft%20Office%20Excel%20工作表%20(2).xlsx#RANGE!gene21) | 90 (1.5%) | ko03018 |
| 22 | [Pentose and glucuronate interconversions](file:///F:\转录组蛋白组\图表\整理图\新建%20Microsoft%20Office%20Excel%20工作表%20(2).xlsx#RANGE!gene22) | 88 (1.46%) | ko00040 |
| 23 | [Plant hormone signal transduction](file:///F:\转录组蛋白组\图表\整理图\新建%20Microsoft%20Office%20Excel%20工作表%20(2).xlsx#RANGE!gene23) | 83 (1.38%) | ko04075 |
| 24 | [Flavonoid biosynthesis](file:///F:\转录组蛋白组\图表\整理图\新建%20Microsoft%20Office%20Excel%20工作表%20(2).xlsx#RANGE!gene24) | 82 (1.36%) | ko00941 |
| 25 | [Glycerophospholipid metabolism](file:///F:\转录组蛋白组\图表\整理图\新建%20Microsoft%20Office%20Excel%20工作表%20(2).xlsx#RANGE!gene25) | 78 (1.3%) | ko00564 |
| 26 | [Oxidative phosphorylation](file:///F:\转录组蛋白组\图表\整理图\新建%20Microsoft%20Office%20Excel%20工作表%20(2).xlsx#RANGE!gene26) | 78 (1.3%) | ko00190 |
| 27 | [Aminoacyl-tRNA biosynthesis](file:///F:\转录组蛋白组\图表\整理图\新建%20Microsoft%20Office%20Excel%20工作表%20(2).xlsx#RANGE!gene27) | 76 (1.26%) | ko00970 |
| 28 | [Pyrimidine metabolism](file:///F:\转录组蛋白组\图表\整理图\新建%20Microsoft%20Office%20Excel%20工作表%20(2).xlsx#RANGE!gene28) | 74 (1.23%) | ko00240 |
| 29 | [Galactose metabolism](file:///F:\转录组蛋白组\图表\整理图\新建%20Microsoft%20Office%20Excel%20工作表%20(2).xlsx#RANGE!gene29) | 73 (1.21%) | ko00052 |
| 30 | [Carbon fixation in photosynthetic organisms](file:///F:\转录组蛋白组\图表\整理图\新建%20Microsoft%20Office%20Excel%20工作表%20(2).xlsx#RANGE!gene30) | 72 (1.2%) | ko00710 |
| 31 | [Cysteine and methionine metabolism](file:///F:\转录组蛋白组\图表\整理图\新建%20Microsoft%20Office%20Excel%20工作表%20(2).xlsx#RANGE!gene31) | 71 (1.18%) | ko00270 |
| 32 | [Fructose and mannose metabolism](file:///F:\转录组蛋白组\图表\整理图\新建%20Microsoft%20Office%20Excel%20工作表%20(2).xlsx#RANGE!gene32) | 71 (1.18%) | ko00051 |
| 33 | [Glutathione metabolism](file:///F:\转录组蛋白组\图表\整理图\新建%20Microsoft%20Office%20Excel%20工作表%20(2).xlsx#RANGE!gene33) | 70 (1.16%) | ko00480 |
| 34 | [Cyanoamino acid metabolism](file:///F:\转录组蛋白组\图表\整理图\新建%20Microsoft%20Office%20Excel%20工作表%20(2).xlsx#RANGE!gene34) | 69 (1.15%) | ko00460 |
| 35 | [Ribosome biogenesis in eukaryotes](file:///F:\转录组蛋白组\图表\整理图\新建%20Microsoft%20Office%20Excel%20工作表%20(2).xlsx#RANGE!gene35) | 68 (1.13%) | ko03008 |
| 36 | [Ascorbate and aldarate metabolism](file:///F:\转录组蛋白组\图表\整理图\新建%20Microsoft%20Office%20Excel%20工作表%20(2).xlsx#RANGE!gene36) | 67 (1.11%) | ko00053 |
| 37 | [Terpenoid backbone biosynthesis](file:///F:\转录组蛋白组\图表\整理图\新建%20Microsoft%20Office%20Excel%20工作表%20(2).xlsx#RANGE!gene37) | 65 (1.08%) | ko00900 |
| 38 | [Fatty acid metabolism](file:///F:\转录组蛋白组\图表\整理图\新建%20Microsoft%20Office%20Excel%20工作表%20(2).xlsx#RANGE!gene38) | 64 (1.06%) | ko01212 |
| 39 | [Glyoxylate and dicarboxylate metabolism](file:///F:\转录组蛋白组\图表\整理图\新建%20Microsoft%20Office%20Excel%20工作表%20(2).xlsx#RANGE!gene39) | 63 (1.05%) | ko00630 |
| 40 | [Porphyrin and chlorophyll metabolism](file:///F:\转录组蛋白组\图表\整理图\新建%20Microsoft%20Office%20Excel%20工作表%20(2).xlsx#RANGE!gene40) | 58 (0.96%) | ko00860 |
| 41 | [Phagosome](file:///F:\转录组蛋白组\图表\整理图\新建%20Microsoft%20Office%20Excel%20工作表%20(2).xlsx#RANGE!gene41) | 58 (0.96%) | ko04145 |
| 42 | [Tyrosine metabolism](file:///F:\转录组蛋白组\图表\整理图\新建%20Microsoft%20Office%20Excel%20工作表%20(2).xlsx#RANGE!gene42) | 58 (0.96%) | ko00350 |
| 43 | [Ubiquitin mediated proteolysis](file:///F:\转录组蛋白组\图表\整理图\新建%20Microsoft%20Office%20Excel%20工作表%20(2).xlsx#RANGE!gene43) | 58 (0.96%) | ko04120 |
| 44 | [2-Oxocarboxylic acid metabolism](file:///F:\转录组蛋白组\图表\整理图\新建%20Microsoft%20Office%20Excel%20工作表%20(2).xlsx#RANGE!gene44) | 57 (0.95%) | ko01210 |
| 45 | [alpha-Linolenic acid metabolism](file:///F:\转录组蛋白组\图表\整理图\新建%20Microsoft%20Office%20Excel%20工作表%20(2).xlsx#RANGE!gene45) | 55 (0.91%) | ko00592 |
| 46 | [Other glycan degradation](file:///F:\转录组蛋白组\图表\整理图\新建%20Microsoft%20Office%20Excel%20工作表%20(2).xlsx#RANGE!gene46) | 54 (0.9%) | ko00511 |
| 47 | [Citrate cycle (TCA cycle)](file:///F:\转录组蛋白组\图表\整理图\新建%20Microsoft%20Office%20Excel%20工作表%20(2).xlsx#RANGE!gene47) | 54 (0.9%) | ko00020 |
| 48 | [beta-Alanine metabolism](file:///F:\转录组蛋白组\图表\整理图\新建%20Microsoft%20Office%20Excel%20工作表%20(2).xlsx#RANGE!gene48) | 53 (0.88%) | ko00410 |
| 49 | [Tryptophan metabolism](file:///F:\转录组蛋白组\图表\整理图\新建%20Microsoft%20Office%20Excel%20工作表%20(2).xlsx#RANGE!gene49) | 52 (0.86%) | ko00380 |
| 50 | [Phenylalanine metabolism](file:///F:\转录组蛋白组\图表\整理图\新建%20Microsoft%20Office%20Excel%20工作表%20(2).xlsx#RANGE!gene50) | 51 (0.85%) | ko00360 |
| 51 | [Proteasome](file:///F:\转录组蛋白组\图表\整理图\新建%20Microsoft%20Office%20Excel%20工作表%20(2).xlsx#RANGE!gene51) | 48 (0.8%) | ko03050 |
| 52 | [Valine, leucine and isoleucine degradation](file:///F:\转录组蛋白组\图表\整理图\新建%20Microsoft%20Office%20Excel%20工作表%20(2).xlsx#RANGE!gene52) | 46 (0.76%) | ko00280 |
| 53 | [Pentose phosphate pathway](file:///F:\转录组蛋白组\图表\整理图\新建%20Microsoft%20Office%20Excel%20工作表%20(2).xlsx#RANGE!gene53) | 45 (0.75%) | ko00030 |
| 54 | [Fatty acid degradation](file:///F:\转录组蛋白组\图表\整理图\新建%20Microsoft%20Office%20Excel%20工作表%20(2).xlsx#RANGE!gene54) | 45 (0.75%) | ko00071 |
| 55 | [Alanine, aspartate and glutamate metabolism](file:///F:\转录组蛋白组\图表\整理图\新建%20Microsoft%20Office%20Excel%20工作表%20(2).xlsx#RANGE!gene55) | 43 (0.71%) | ko00250 |
| 56 | [Arginine and proline metabolism](file:///F:\转录组蛋白组\图表\整理图\新建%20Microsoft%20Office%20Excel%20工作表%20(2).xlsx#RANGE!gene56) | 43 (0.71%) | ko00330 |
| 57 | [ABC transporters](file:///F:\转录组蛋白组\图表\整理图\新建%20Microsoft%20Office%20Excel%20工作表%20(2).xlsx#RANGE!gene57) | 42 (0.7%) | ko02010 |
| 58 | [Inositol phosphate metabolism](file:///F:\转录组蛋白组\图表\整理图\新建%20Microsoft%20Office%20Excel%20工作表%20(2).xlsx#RANGE!gene58) | 41 (0.68%) | ko00562 |
| 59 | [Fatty acid biosynthesis](file:///F:\转录组蛋白组\图表\整理图\新建%20Microsoft%20Office%20Excel%20工作表%20(2).xlsx#RANGE!gene59) | 40 (0.66%) | ko00061 |
| 60 | [Sphingolipid metabolism](file:///F:\转录组蛋白组\图表\整理图\新建%20Microsoft%20Office%20Excel%20工作表%20(2).xlsx#RANGE!gene60) | 40 (0.66%) | ko00600 |
| 61 | [Cutin, suberine and wax biosynthesis](file:///F:\转录组蛋白组\图表\整理图\新建%20Microsoft%20Office%20Excel%20工作表%20(2).xlsx#RANGE!gene61) | 40 (0.66%) | ko00073 |
| 62 | [Carotenoid biosynthesis](file:///F:\转录组蛋白组\图表\整理图\新建%20Microsoft%20Office%20Excel%20工作表%20(2).xlsx#RANGE!gene62) | 40 (0.66%) | ko00906 |
| 63 | [Propanoate metabolism](file:///F:\转录组蛋白组\图表\整理图\新建%20Microsoft%20Office%20Excel%20工作表%20(2).xlsx#RANGE!gene63) | 39 (0.65%) | ko00640 |
| 64 | [Phenylalanine, tyrosine and tryptophan biosynthesis](file:///F:\转录组蛋白组\图表\整理图\新建%20Microsoft%20Office%20Excel%20工作表%20(2).xlsx#RANGE!gene64) | 39 (0.65%) | ko00400 |
| 65 | [Phosphatidylinositol signaling system](file:///F:\转录组蛋白组\图表\整理图\新建%20Microsoft%20Office%20Excel%20工作表%20(2).xlsx#RANGE!gene65) | 36 (0.6%) | ko04070 |
| 66 | [Vitamin B6 metabolism](file:///F:\转录组蛋白组\图表\整理图\新建%20Microsoft%20Office%20Excel%20工作表%20(2).xlsx#RANGE!gene66) | 36 (0.6%) | ko00750 |
| 67 | [Stilbenoid, diarylheptanoid and gingerol biosynthesis](file:///F:\转录组蛋白组\图表\整理图\新建%20Microsoft%20Office%20Excel%20工作表%20(2).xlsx#RANGE!gene67) | 35 (0.58%) | ko00945 |
| 68 | [Linoleic acid metabolism](file:///F:\转录组蛋白组\图表\整理图\新建%20Microsoft%20Office%20Excel%20工作表%20(2).xlsx#RANGE!gene68) | 34 (0.57%) | ko00591 |
| 69 | [Circadian rhythm - plant](file:///F:\转录组蛋白组\图表\整理图\新建%20Microsoft%20Office%20Excel%20工作表%20(2).xlsx#RANGE!gene69) | 34 (0.57%) | ko04712 |
| 70 | [Nucleotide excision repair](file:///F:\转录组蛋白组\图表\整理图\新建%20Microsoft%20Office%20Excel%20工作表%20(2).xlsx#RANGE!gene70) | 33 (0.55%) | ko03420 |
| 71 | [Limonene and pinene degradation](file:///F:\转录组蛋白组\图表\整理图\新建%20Microsoft%20Office%20Excel%20工作表%20(2).xlsx#RANGE!gene71) | 32 (0.53%) | ko00903 |
| 72 | [Arginine biosynthesis](file:///F:\转录组蛋白组\图表\整理图\新建%20Microsoft%20Office%20Excel%20工作表%20(2).xlsx#RANGE!gene72) | 32 (0.53%) | ko00220 |
| 73 | [Photosynthesis](file:///F:\转录组蛋白组\图表\整理图\新建%20Microsoft%20Office%20Excel%20工作表%20(2).xlsx#RANGE!gene73) | 32 (0.53%) | ko00195 |
| 74 | [DNA replication](file:///F:\转录组蛋白组\图表\整理图\新建%20Microsoft%20Office%20Excel%20工作表%20(2).xlsx#RANGE!gene74) | 32 (0.53%) | ko03030 |
| 75 | [Arachidonic acid metabolism](file:///F:\转录组蛋白组\图表\整理图\新建%20Microsoft%20Office%20Excel%20工作表%20(2).xlsx#RANGE!gene75) | 30 (0.5%) | ko00590 |
| 76 | [Flavone and flavonol biosynthesis](file:///F:\转录组蛋白组\图表\整理图\新建%20Microsoft%20Office%20Excel%20工作表%20(2).xlsx#RANGE!gene76) | 30 (0.5%) | ko00944 |
| 77 | [Protein export](file:///F:\转录组蛋白组\图表\整理图\新建%20Microsoft%20Office%20Excel%20工作表%20(2).xlsx#RANGE!gene77) | 29 (0.48%) | ko03060 |
| 78 | [Isoquinoline alkaloid biosynthesis](file:///F:\转录组蛋白组\图表\整理图\新建%20Microsoft%20Office%20Excel%20工作表%20(2).xlsx#RANGE!gene78) | 29 (0.48%) | ko00950 |
| 79 | [Insulin resistance](file:///F:\转录组蛋白组\图表\整理图\新建%20Microsoft%20Office%20Excel%20工作表%20(2).xlsx#RANGE!gene79) | 29 (0.48%) | ko04931 |
| 80 | [Diterpenoid biosynthesis](file:///F:\转录组蛋白组\图表\整理图\新建%20Microsoft%20Office%20Excel%20工作表%20(2).xlsx#RANGE!gene80) | 29 (0.48%) | ko00904 |
| 81 | [Biosynthesis of unsaturated fatty acids](file:///F:\转录组蛋白组\图表\整理图\新建%20Microsoft%20Office%20Excel%20工作表%20(2).xlsx#RANGE!gene81) | 28 (0.47%) | ko01040 |
| 82 | [N-Glycan biosynthesis](file:///F:\转录组蛋白组\图表\整理图\新建%20Microsoft%20Office%20Excel%20工作表%20(2).xlsx#RANGE!gene82) | 27 (0.45%) | ko00510 |
| 83 | [Nitrogen metabolism](file:///F:\转录组蛋白组\图表\整理图\新建%20Microsoft%20Office%20Excel%20工作表%20(2).xlsx#RANGE!gene83) | 27 (0.45%) | ko00910 |
| 84 | [Ubiquinone and other terpenoid-quinone biosynthesis](file:///F:\转录组蛋白组\图表\整理图\新建%20Microsoft%20Office%20Excel%20工作表%20(2).xlsx#RANGE!gene84) | 27 (0.45%) | ko00130 |
| 85 | [Sulfur metabolism](file:///F:\转录组蛋白组\图表\整理图\新建%20Microsoft%20Office%20Excel%20工作表%20(2).xlsx#RANGE!gene85) | 26 (0.43%) | ko00920 |
| 86 | [Regulation of autophagy](file:///F:\转录组蛋白组\图表\整理图\新建%20Microsoft%20Office%20Excel%20工作表%20(2).xlsx#RANGE!gene86) | 26 (0.43%) | ko04140 |
| 87 | [Tropane, piperidine and pyridine alkaloid biosynthesis](file:///F:\转录组蛋白组\图表\整理图\新建%20Microsoft%20Office%20Excel%20工作表%20(2).xlsx#RANGE!gene87) | 26 (0.43%) | ko00960 |
| 88 | [SNARE interactions in vesicular transport](file:///F:\转录组蛋白组\图表\整理图\新建%20Microsoft%20Office%20Excel%20工作表%20(2).xlsx#RANGE!gene88) | 26 (0.43%) | ko04130 |
| 89 | [Steroid biosynthesis](file:///F:\转录组蛋白组\图表\整理图\新建%20Microsoft%20Office%20Excel%20工作表%20(2).xlsx#RANGE!gene89) | 24 (0.4%) | ko00100 |
| 90 | [Mismatch repair](file:///F:\转录组蛋白组\图表\整理图\新建%20Microsoft%20Office%20Excel%20工作表%20(2).xlsx#RANGE!gene90) | 23 (0.38%) | ko03430 |
| 91 | [Lysine degradation](file:///F:\转录组蛋白组\图表\整理图\新建%20Microsoft%20Office%20Excel%20工作表%20(2).xlsx#RANGE!gene91) | 23 (0.38%) | ko00310 |
| 92 | [Biotin metabolism](file:///F:\转录组蛋白组\图表\整理图\新建%20Microsoft%20Office%20Excel%20工作表%20(2).xlsx#RANGE!gene92) | 22 (0.37%) | ko00780 |
| 93 | [Zeatin biosynthesis](file:///F:\转录组蛋白组\图表\整理图\新建%20Microsoft%20Office%20Excel%20工作表%20(2).xlsx#RANGE!gene93) | 21 (0.35%) | ko00908 |
| 94 | [Basal transcription factors](file:///F:\转录组蛋白组\图表\整理图\新建%20Microsoft%20Office%20Excel%20工作表%20(2).xlsx#RANGE!gene94) | 21 (0.35%) | ko03022 |
| 95 | [Ether lipid metabolism](file:///F:\转录组蛋白组\图表\整理图\新建%20Microsoft%20Office%20Excel%20工作表%20(2).xlsx#RANGE!gene95) | 21 (0.35%) | ko00565 |
| 96 | [RNA polymerase](file:///F:\转录组蛋白组\图表\整理图\新建%20Microsoft%20Office%20Excel%20工作表%20(2).xlsx#RANGE!gene96) | 21 (0.35%) | ko03020 |
| 97 | [Glycosphingolipid biosynthesis - globo series](file:///F:\转录组蛋白组\图表\整理图\新建%20Microsoft%20Office%20Excel%20工作表%20(2).xlsx#RANGE!gene97) | 20 (0.33%) | ko00603 |
| 98 | [Base excision repair](file:///F:\转录组蛋白组\图表\整理图\新建%20Microsoft%20Office%20Excel%20工作表%20(2).xlsx#RANGE!gene98) | 20 (0.33%) | ko03410 |
| 99 | [Homologous recombination](file:///F:\转录组蛋白组\图表\整理图\新建%20Microsoft%20Office%20Excel%20工作表%20(2).xlsx#RANGE!gene99) | 20 (0.33%) | ko03440 |
| 100 | [Pantothenate and CoA biosynthesis](file:///F:\转录组蛋白组\图表\整理图\新建%20Microsoft%20Office%20Excel%20工作表%20(2).xlsx#RANGE!gene100) | 20 (0.33%) | ko00770 |
| 101 | [Butanoate metabolism](file:///F:\转录组蛋白组\图表\整理图\新建%20Microsoft%20Office%20Excel%20工作表%20(2).xlsx#RANGE!gene101) | 20 (0.33%) | ko00650 |
| 102 | [Glycosaminoglycan degradation](file:///F:\转录组蛋白组\图表\整理图\新建%20Microsoft%20Office%20Excel%20工作表%20(2).xlsx#RANGE!gene102) | 19 (0.32%) | ko00531 |
| 103 | [Histidine metabolism](file:///F:\转录组蛋白组\图表\整理图\新建%20Microsoft%20Office%20Excel%20工作表%20(2).xlsx#RANGE!gene103) | 19 (0.32%) | ko00340 |
| 104 | [Nicotinate and nicotinamide metabolism](file:///F:\转录组蛋白组\图表\整理图\新建%20Microsoft%20Office%20Excel%20工作表%20(2).xlsx#RANGE!gene104) | 19 (0.32%) | ko00760 |
| 105 | [One carbon pool by folate](file:///F:\转录组蛋白组\图表\整理图\新建%20Microsoft%20Office%20Excel%20工作表%20(2).xlsx#RANGE!gene105) | 18 (0.3%) | ko00670 |
| 106 | [Selenocompound metabolism](file:///F:\转录组蛋白组\图表\整理图\新建%20Microsoft%20Office%20Excel%20工作表%20(2).xlsx#RANGE!gene106) | 17 (0.28%) | ko00450 |
| 107 | [Brassinosteroid biosynthesis](file:///F:\转录组蛋白组\图表\整理图\新建%20Microsoft%20Office%20Excel%20工作表%20(2).xlsx#RANGE!gene107) | 17 (0.28%) | ko00905 |
| 108 | [Fatty acid elongation](file:///F:\转录组蛋白组\图表\整理图\新建%20Microsoft%20Office%20Excel%20工作表%20(2).xlsx#RANGE!gene108) | 16 (0.27%) | ko00062 |
| 109 | [Indole alkaloid biosynthesis](file:///F:\转录组蛋白组\图表\整理图\新建%20Microsoft%20Office%20Excel%20工作表%20(2).xlsx#RANGE!gene109) | 16 (0.27%) | ko00901 |
| 110 | [Valine, leucine and isoleucine biosynthesis](file:///F:\转录组蛋白组\图表\整理图\新建%20Microsoft%20Office%20Excel%20工作表%20(2).xlsx#RANGE!gene110) | 16 (0.27%) | ko00290 |
| 111 | [Riboflavin metabolism](file:///F:\转录组蛋白组\图表\整理图\新建%20Microsoft%20Office%20Excel%20工作表%20(2).xlsx#RANGE!gene111) | 14 (0.23%) | ko00740 |
| 112 | [Monoterpenoid biosynthesis](file:///F:\转录组蛋白组\图表\整理图\新建%20Microsoft%20Office%20Excel%20工作表%20(2).xlsx#RANGE!gene112) | 14 (0.23%) | ko00902 |
| 113 | [Glucosinolate biosynthesis](file:///F:\转录组蛋白组\图表\整理图\新建%20Microsoft%20Office%20Excel%20工作表%20(2).xlsx#RANGE!gene113) | 14 (0.23%) | ko00966 |
| 114 | [Degradation of aromatic compounds](file:///F:\转录组蛋白组\图表\整理图\新建%20Microsoft%20Office%20Excel%20工作表%20(2).xlsx#RANGE!gene114) | 13 (0.22%) | ko01220 |
| 115 | [Glycosphingolipid biosynthesis - ganglio series](file:///F:\转录组蛋白组\图表\整理图\新建%20Microsoft%20Office%20Excel%20工作表%20(2).xlsx#RANGE!gene115) | 13 (0.22%) | ko00604 |
| 116 | [Photosynthesis - antenna proteins](file:///F:\转录组蛋白组\图表\整理图\新建%20Microsoft%20Office%20Excel%20工作表%20(2).xlsx#RANGE!gene116) | 13 (0.22%) | ko00196 |
| 117 | [Caffeine metabolism](file:///F:\转录组蛋白组\图表\整理图\新建%20Microsoft%20Office%20Excel%20工作表%20(2).xlsx#RANGE!gene117) | 13 (0.22%) | ko00232 |
| 118 | [Folate biosynthesis](file:///F:\转录组蛋白组\图表\整理图\新建%20Microsoft%20Office%20Excel%20工作表%20(2).xlsx#RANGE!gene118) | 13 (0.22%) | ko00790 |
| 119 | [Lysine biosynthesis](file:///F:\转录组蛋白组\图表\整理图\新建%20Microsoft%20Office%20Excel%20工作表%20(2).xlsx#RANGE!gene119) | 10 (0.17%) | ko00300 |
| 120 | [Other types of O-glycan biosynthesis](file:///F:\转录组蛋白组\图表\整理图\新建%20Microsoft%20Office%20Excel%20工作表%20(2).xlsx#RANGE!gene120) | 9 (0.15%) | ko00514 |
| 121 | [Sulfur relay system](file:///F:\转录组蛋白组\图表\整理图\新建%20Microsoft%20Office%20Excel%20工作表%20(2).xlsx#RANGE!gene121) | 8 (0.13%) | ko04122 |
| 122 | [C5-Branched dibasic acid metabolism](file:///F:\转录组蛋白组\图表\整理图\新建%20Microsoft%20Office%20Excel%20工作表%20(2).xlsx#RANGE!gene122) | 8 (0.13%) | ko00660 |
| 123 | [Anthocyanin biosynthesis](file:///F:\转录组蛋白组\图表\整理图\新建%20Microsoft%20Office%20Excel%20工作表%20(2).xlsx#RANGE!gene123) | 8 (0.13%) | ko00942 |
| 124 | [Monobactam biosynthesis](file:///F:\转录组蛋白组\图表\整理图\新建%20Microsoft%20Office%20Excel%20工作表%20(2).xlsx#RANGE!gene124) | 7 (0.12%) | ko00261 |
| 125 | [Glycosylphosphatidylinositol(GPI)-anchor biosynthesis](file:///F:\转录组蛋白组\图表\整理图\新建%20Microsoft%20Office%20Excel%20工作表%20(2).xlsx#RANGE!gene125) | 6 (0.1%) | ko00563 |
| 126 | [Betalain biosynthesis](file:///F:\转录组蛋白组\图表\整理图\新建%20Microsoft%20Office%20Excel%20工作表%20(2).xlsx#RANGE!gene126) | 6 (0.1%) | ko00965 |
| 127 | [Non-homologous end-joining](file:///F:\转录组蛋白组\图表\整理图\新建%20Microsoft%20Office%20Excel%20工作表%20(2).xlsx#RANGE!gene127) | 6 (0.1%) | ko03450 |
| 128 | [Taurine and hypotaurine metabolism](file:///F:\转录组蛋白组\图表\整理图\新建%20Microsoft%20Office%20Excel%20工作表%20(2).xlsx#RANGE!gene128) | 5 (0.08%) | ko00430 |
| 129 | [Sesquiterpenoid and triterpenoid biosynthesis](file:///F:\转录组蛋白组\图表\整理图\新建%20Microsoft%20Office%20Excel%20工作表%20(2).xlsx#RANGE!gene129) | 5 (0.08%) | ko00909 |
| 130 | [Thiamine metabolism](file:///F:\转录组蛋白组\图表\整理图\新建%20Microsoft%20Office%20Excel%20工作表%20(2).xlsx#RANGE!gene130) | 5 (0.08%) | ko00730 |
| 131 | [Synthesis and degradation of ketone bodies](file:///F:\转录组蛋白组\图表\整理图\新建%20Microsoft%20Office%20Excel%20工作表%20(2).xlsx#RANGE!gene131) | 4 (0.07%) | ko00072 |
| 132 | [Isoflavonoid biosynthesis](file:///F:\转录组蛋白组\图表\整理图\新建%20Microsoft%20Office%20Excel%20工作表%20(2).xlsx#RANGE!gene132) | 2 (0.03%) | ko00943 |
| 133 | [Lipoic acid metabolism](file:///F:\转录组蛋白组\图表\整理图\新建%20Microsoft%20Office%20Excel%20工作表%20(2).xlsx#RANGE!gene133) | 2 (0.03%) | ko00785 |
| 134 | [Vancomycin resistance](file:///F:\转录组蛋白组\图表\整理图\新建%20Microsoft%20Office%20Excel%20工作表%20(2).xlsx#RANGE!gene134) | 2 (0.03%) | ko01502 |
