# Supplementary material for: An Integrated Transcriptome and Proteome Analysis Reveals Putative Regulators of Adventitious Root Formation in Taxodium ‘Zhongshanshan’
Source: Int J Mol Sci. 2019 Mar 11;20(5):1225. doi: 10.3390/ijms20051225 (PMC6429173; doi:10.3390/ijms20051225)
Supplement: Supplementary file 1 [file ijms-20-01225-s001.zip › Supplementary material20190227/Table S7.docx]

**Table S7** The result of Gene Ontology (GO) classification and functional enrichment of DEPs

|  |  | S1-VS-S0_ Cellular Component |  |  |
| --- | --- | --- | --- | --- |
|  | **Gene Ontology term** | **Cluster frequency** | **Protein frequency of use** | **P-value** |
| 1 | chloroplast | 132 out of 389 genes, 33.9% | 529 out of 2292 genes, 23.1% | 4.99E-08 |
| 2 | thylakoid | 46 out of 389 genes, 11.8% | 128 out of 2292 genes, 5.6% | 7.57E-08 |
| 3 | plastid | 144 out of 389 genes, 37.0% | 612 out of 2292 genes, 26.7% | 5.86E-07 |
| 4 | plastid part | 74 out of 389 genes, 19.0% | 262 out of 2292 genes, 11.4% | 8.09E-07 |
| 5 | chloroplast part | 73 out of 389 genes, 18.8% | 258 out of 2292 genes, 11.3% | 9.06E-07 |
| 6 | photosynthetic membrane | 33 out of 389 genes, 8.5% | 90 out of 2292 genes, 3.9% | 3.65E-06 |
| 7 | apoplast | 31 out of 389 genes, 8.0% | 83 out of 2292 genes, 3.6% | 4.67E-06 |
| 8 | thylakoid membrane | 30 out of 389 genes, 7.7% | 80 out of 2292 genes, 3.5% | 6.09E-06 |
| 9 | extracellular region | 48 out of 389 genes, 12.3% | 155 out of 2292 genes, 6.8% | 6.15E-06 |
| 10 | thylakoid part | 33 out of 389 genes, 8.5% | 95 out of 2292 genes, 4.1% | 1.41E-05 |
| 11 | chloroplast thylakoid membrane | 27 out of 389 genes, 6.9% | 74 out of 2292 genes, 3.2% | 3.24E-05 |
| 12 | plastid thylakoid membrane | 27 out of 389 genes, 6.9% | 74 out of 2292 genes, 3.2% | 3.24E-05 |
| 13 | photosystem II | 9 out of 389 genes, 2.3% | 13 out of 2292 genes, 0.6% | 4.05E-05 |
| 14 | photosystem | 11 out of 389 genes, 2.8% | 20 out of 2292 genes, 0.9% | 0.000115 |
| 15 | chloroplast thylakoid | 28 out of 389 genes, 7.2% | 84 out of 2292 genes, 3.7% | 0.000149 |
| 16 | plastid thylakoid | 28 out of 389 genes, 7.2% | 84 out of 2292 genes, 3.7% | 0.000149 |
| 17 | organelle subcompartment | 28 out of 389 genes, 7.2% | 85 out of 2292 genes, 3.7% | 0.000188 |
| 18 | plastid stroma | 41 out of 389 genes, 10.5% | 149 out of 2292 genes, 6.5% | 0.000574 |
| 19 | chloroplast stroma | 40 out of 389 genes, 10.3% | 146 out of 2292 genes, 6.4% | 0.000739 |
| 20 | cytoplasmic part | 257 out of 389 genes, 66.1% | 1358 out of 2292 genes, 59.2% | 0.001498 |
| 21 | plastid envelope | 37 out of 389 genes, 9.5% | 139 out of 2292 genes, 6.1% | 0.002082 |
| 22 | cytoplasm | 294 out of 389 genes, 75.6% | 1596 out of 2292 genes, 69.6% | 0.002745 |
| 23 | nucleosome | 9 out of 389 genes, 2.3% | 21 out of 2292 genes, 0.9% | 0.004617 |
| 24 | protein-DNA complex | 9 out of 389 genes, 2.3% | 21 out of 2292 genes, 0.9% | 0.004617 |
| 25 | organelle part | 124 out of 389 genes, 31.9% | 605 out of 2292 genes, 26.4% | 0.004747 |
| 26 | intracellular organelle part | 124 out of 389 genes, 31.9% | 605 out of 2292 genes, 26.4% | 0.004747 |
| 27 | glycine cleavage complex | 3 out of 389 genes, 0.8% | 3 out of 2292 genes, 0.1% | 0.004858 |
| 28 | chloroplast envelope | 34 out of 389 genes, 8.7% | 134 out of 2292 genes, 5.8% | 0.007178 |
| 29 | nucleolus | 28 out of 389 genes, 7.2% | 106 out of 2292 genes, 4.6% | 0.00804 |
| 30 | non-membrane-bounded organelle | 65 out of 389 genes, 16.7% | 293 out of 2292 genes, 12.8% | 0.00816 |
| 31 | intracellular non-membrane-bounded organelle | 65 out of 389 genes, 16.7% | 293 out of 2292 genes, 12.8% | 0.00816 |
| 32 | intracellular part | 324 out of 389 genes, 83.3% | 1804 out of 2292 genes, 78.7% | 0.008221 |
| 33 | chromatin | 9 out of 389 genes, 2.3% | 24 out of 2292 genes, 1.0% | 0.012771 |
| 34 | chromosomal part | 9 out of 389 genes, 2.3% | 24 out of 2292 genes, 1.0% | 0.012771 |
| 35 | chloroplast thylakoid lumen | 5 out of 389 genes, 1.3% | 10 out of 2292 genes, 0.4% | 0.016466 |
| 36 | plastid thylakoid lumen | 5 out of 389 genes, 1.3% | 10 out of 2292 genes, 0.4% | 0.016466 |
| 37 | organelle | 273 out of 389 genes, 70.2% | 1499 out of 2292 genes, 65.4% | 0.016531 |
| 38 | intracellular organelle | 273 out of 389 genes, 70.2% | 1499 out of 2292 genes, 65.4% | 0.016531 |
| 39 | peroxisome | 13 out of 389 genes, 3.3% | 43 out of 2292 genes, 1.9% | 0.021678 |
| 40 | microbody | 13 out of 389 genes, 3.3% | 43 out of 2292 genes, 1.9% | 0.021678 |
| 41 | thylakoid lumen | 7 out of 389 genes, 1.8% | 18 out of 2292 genes, 0.8% | 0.022203 |
| 42 | ribosome | 36 out of 389 genes, 9.3% | 155 out of 2292 genes, 6.8% | 0.023795 |
| 43 | organelle lumen | 31 out of 389 genes, 8.0% | 130 out of 2292 genes, 5.7% | 0.024508 |
| 44 | intracellular organelle lumen | 31 out of 389 genes, 8.0% | 130 out of 2292 genes, 5.7% | 0.024508 |
| 45 | membrane-bounded organelle | 254 out of 389 genes, 65.3% | 1393 out of 2292 genes, 60.8% | 0.025294 |
| 46 | intracellular membrane-bounded organelle | 254 out of 389 genes, 65.3% | 1393 out of 2292 genes, 60.8% | 0.025294 |
| 47 | intracellular | 332 out of 389 genes, 85.3% | 1877 out of 2292 genes, 81.9% | 0.028899 |
| 48 | plastoglobule | 6 out of 389 genes, 1.5% | 15 out of 2292 genes, 0.7% | 0.029294 |
| 49 | cell wall | 38 out of 389 genes, 9.8% | 168 out of 2292 genes, 7.3% | 0.030555 |
| 50 | external encapsulating structure | 38 out of 389 genes, 9.8% | 168 out of 2292 genes, 7.3% | 0.030555 |
| 51 | cytosolic ribosome | 21 out of 389 genes, 5.4% | 83 out of 2292 genes, 3.6% | 0.032491 |
| 52 | membrane-enclosed lumen | 31 out of 389 genes, 8.0% | 133 out of 2292 genes, 5.8% | 0.033118 |
| 53 | macromolecular complex | 94 out of 389 genes, 24.2% | 473 out of 2292 genes, 20.6% | 0.035987 |
| 54 | plant-type cell wall | 13 out of 389 genes, 3.3% | 46 out of 2292 genes, 2.0% | 0.037208 |
| 55 | chromosome | 10 out of 389 genes, 2.6% | 33 out of 2292 genes, 1.4% | 0.041128 |
| 56 | ribonucleoprotein complex | 40 out of 389 genes, 10.3% | 183 out of 2292 genes, 8.0% | 0.044576 |
| 57 | cytosol | 57 out of 389 genes, 14.7% | 274 out of 2292 genes, 12.0% | 0.045542 |
| 58 | nuclear lumen | 28 out of 389 genes, 7.2% | 122 out of 2292 genes, 5.3% | 0.049844 |
|  |  | **S1-VS-S0_ Molecular Function** |  |  |
|  | **Gene Ontology term** | **Cluster frequency** | **Protein frequency of use** | **P-value** |
| 1 | oxidoreductase activity | 138 out of 568 genes, 24.3% | 635 out of 3345 genes, 19.0% | 0.000329 |
| 2 | oxidoreductase activity, acting on the CH-CH group of donors, NAD or NADP as acceptor | 13 out of 568 genes, 2.3% | 30 out of 3345 genes, 0.9% | 0.00061 |
| 3 | magnesium ion binding | 19 out of 568 genes, 3.3% | 53 out of 3345 genes, 1.6% | 0.000675 |
| 4 | oxidoreductase activity, acting on the CH-CH group of donors | 16 out of 568 genes, 2.8% | 44 out of 3345 genes, 1.3% | 0.001492 |
| 5 | tetrapyrrole binding | 20 out of 568 genes, 3.5% | 62 out of 3345 genes, 1.9% | 0.00221 |
| 6 | cofactor binding | 50 out of 568 genes, 8.8% | 202 out of 3345 genes, 6.0% | 0.002396 |
| 7 | antioxidant activity | 19 out of 568 genes, 3.3% | 59 out of 3345 genes, 1.8% | 0.002891 |
| 8 | coenzyme binding | 36 out of 568 genes, 6.3% | 137 out of 3345 genes, 4.1% | 0.00336 |
| 9 | cation binding | 124 out of 568 genes, 21.8% | 593 out of 3345 genes, 17.7% | 0.00347 |
| 10 | chlorophyll binding | 3 out of 568 genes, 0.5% | 3 out of 3345 genes, 0.1% | 0.004875 |
| 11 | 2-alkenal reductase [NAD(P)] activity | 8 out of 568 genes, 1.4% | 18 out of 3345 genes, 0.5% | 0.005867 |
| 12 | oxidoreductase activity, acting on CH-OH group of donors | 29 out of 568 genes, 5.1% | 114 out of 3345 genes, 3.4% | 0.012939 |
| 13 | hydrolase activity, acting on glycosyl bonds | 38 out of 568 genes, 6.7% | 159 out of 3345 genes, 4.8% | 0.013988 |
| 14 | ion binding | 211 out of 568 genes, 37.1% | 1107 out of 3345 genes, 33.1% | 0.014204 |
| 15 | oxidoreductase activity, acting on the CH-OH group of donors, NAD or NADP as acceptor | 25 out of 568 genes, 4.4% | 96 out of 3345 genes, 2.9% | 0.015103 |
| 16 | isomerase activity | 26 out of 568 genes, 4.6% | 101 out of 3345 genes, 3.0% | 0.015523 |
| 17 | heme binding | 17 out of 568 genes, 3.0% | 59 out of 3345 genes, 1.8% | 0.015727 |
| 18 | intramolecular oxidoreductase activity, interconverting aldoses and ketoses | 5 out of 568 genes, 0.9% | 10 out of 3345 genes, 0.3% | 0.016571 |
| 19 | peroxidase activity | 14 out of 568 genes, 2.5% | 46 out of 3345 genes, 1.4% | 0.016849 |
| 20 | oxidoreductase activity, acting on peroxide as acceptor | 14 out of 568 genes, 2.5% | 46 out of 3345 genes, 1.4% | 0.016849 |
| 21 | superoxide dismutase activity | 3 out of 568 genes, 0.5% | 4 out of 3345 genes, 0.1% | 0.017026 |
| 22 | oxidoreductase activity, acting on superoxide radicals as acceptor | 3 out of 568 genes, 0.5% | 4 out of 3345 genes, 0.1% | 0.017026 |
| 23 | ribosome binding | 3 out of 568 genes, 0.5% | 4 out of 3345 genes, 0.1% | 0.017026 |
| 24 | metal ion binding | 110 out of 568 genes, 19.4% | 545 out of 3345 genes, 16.3% | 0.018528 |
| 25 | phosphotransferase activity, phosphate group as acceptor | 4 out of 568 genes, 0.7% | 7 out of 3345 genes, 0.2% | 0.018721 |
| 26 | carbon-oxygen lyase activity, acting on phosphates | 4 out of 568 genes, 0.7% | 7 out of 3345 genes, 0.2% | 0.018721 |
| 27 | malate dehydrogenase activity | 6 out of 568 genes, 1.1% | 14 out of 3345 genes, 0.4% | 0.020585 |
| 28 | binding | 334 out of 568 genes, 58.8% | 1837 out of 3345 genes, 54.9% | 0.022769 |
| 29 | hydrolase activity, hydrolyzing O-glycosyl compounds | 30 out of 568 genes, 5.3% | 125 out of 3345 genes, 3.7% | 0.025787 |
| 30 | copper ion binding | 16 out of 568 genes, 2.8% | 58 out of 3345 genes, 1.7% | 0.028193 |
| 31 | aminomethyltransferase activity | 2 out of 568 genes, 0.4% | 2 out of 3345 genes, 0.1% | 0.028792 |
| 32 | glycine dehydrogenase (decarboxylating) activity | 2 out of 568 genes, 0.4% | 2 out of 3345 genes, 0.1% | 0.028792 |
| 33 | thiosulfate sulfurtransferase activity | 2 out of 568 genes, 0.4% | 2 out of 3345 genes, 0.1% | 0.028792 |
| 34 | triose-phosphate isomerase activity | 2 out of 568 genes, 0.4% | 2 out of 3345 genes, 0.1% | 0.028792 |
| 35 | oxidoreductase activity, acting on the CH-NH2 group of donors, disulfide as acceptor | 2 out of 568 genes, 0.4% | 2 out of 3345 genes, 0.1% | 0.028792 |
| 36 | amidine-lyase activity | 2 out of 568 genes, 0.4% | 2 out of 3345 genes, 0.1% | 0.028792 |
| 37 | intramolecular transferase activity, transferring amino groups | 2 out of 568 genes, 0.4% | 2 out of 3345 genes, 0.1% | 0.028792 |
| 38 | glycopeptide alpha-N-acetylgalactosaminidase activity | 2 out of 568 genes, 0.4% | 2 out of 3345 genes, 0.1% | 0.028792 |
| 39 | glutamate-1-semialdehyde 2,1-aminomutase activity | 2 out of 568 genes, 0.4% | 2 out of 3345 genes, 0.1% | 0.028792 |
| 40 | electron transporter, transferring electrons within the cyclic electron transport pathway of photosynthesis activity | 2 out of 568 genes, 0.4% | 2 out of 3345 genes, 0.1% | 0.028792 |
| 41 | intramolecular oxidoreductase activity | 6 out of 568 genes, 1.1% | 15 out of 3345 genes, 0.4% | 0.029504 |
| 42 | chitinase activity | 3 out of 568 genes, 0.5% | 5 out of 3345 genes, 0.1% | 0.03722 |
| 43 | L-malate dehydrogenase activity | 3 out of 568 genes, 0.5% | 5 out of 3345 genes, 0.1% | 0.03722 |
| 44 | fructose 1,6-bisphosphate 1-phosphatase activity | 3 out of 568 genes, 0.5% | 5 out of 3345 genes, 0.1% | 0.03722 |
| 45 | ribonucleoprotein complex binding | 3 out of 568 genes, 0.5% | 5 out of 3345 genes, 0.1% | 0.03722 |
| 46 | NAD binding | 11 out of 568 genes, 1.9% | 37 out of 3345 genes, 1.1% | 0.038204 |
| 47 | NADP binding | 8 out of 568 genes, 1.4% | 24 out of 3345 genes, 0.7% | 0.038577 |
| 48 | lyase activity | 29 out of 568 genes, 5.1% | 125 out of 3345 genes, 3.7% | 0.042516 |
| 49 | carbon-oxygen lyase activity | 12 out of 568 genes, 2.1% | 43 out of 3345 genes, 1.3% | 0.049252 |
|  |  | **S1-VS-S0_ Biological Process** |  |  |
|  | **Gene Ontology term** | **Cluster frequency** | **Protein frequency of use** | **P-value** |
| 1 | photosynthesis | 34 out of 501 genes, 6.8% | 69 out of 2860 genes, 2.4% | 8.85E-10 |
| 2 | photosynthesis, light reaction | 23 out of 501 genes, 4.6% | 43 out of 2860 genes, 1.5% | 7.40E-08 |
| 3 | oxidation-reduction process | 116 out of 501 genes, 23.2% | 453 out of 2860 genes, 15.8% | 1.40E-06 |
| 4 | glycine catabolic process | 7 out of 501 genes, 1.4% | 7 out of 2860 genes, 0.2% | 4.89E-06 |
| 5 | serine family amino acid catabolic process | 7 out of 501 genes, 1.4% | 7 out of 2860 genes, 0.2% | 4.89E-06 |
| 6 | single-organism metabolic process | 212 out of 501 genes, 42.3% | 981 out of 2860 genes, 34.3% | 2.43E-05 |
| 7 | alpha-amino acid catabolic process | 15 out of 501 genes, 3.0% | 31 out of 2860 genes, 1.1% | 7.02E-05 |
| 8 | photosynthesis, light harvesting | 10 out of 501 genes, 2.0% | 16 out of 2860 genes, 0.6% | 7.30E-05 |
| 9 | cellular amino acid catabolic process | 15 out of 501 genes, 3.0% | 32 out of 2860 genes, 1.1% | 0.000111 |
| 10 | photosynthetic electron transport chain | 9 out of 501 genes, 1.8% | 14 out of 2860 genes, 0.5% | 0.000126 |
| 11 | organic acid catabolic process | 20 out of 501 genes, 4.0% | 51 out of 2860 genes, 1.8% | 0.000182 |
| 12 | small molecule catabolic process | 20 out of 501 genes, 4.0% | 51 out of 2860 genes, 1.8% | 0.000182 |
| 13 | single-organism catabolic process | 20 out of 501 genes, 4.0% | 51 out of 2860 genes, 1.8% | 0.000182 |
| 14 | carboxylic acid catabolic process | 20 out of 501 genes, 4.0% | 51 out of 2860 genes, 1.8% | 0.000182 |
| 15 | glycine metabolic process | 7 out of 501 genes, 1.4% | 10 out of 2860 genes, 0.3% | 0.000359 |
| 16 | chlorophyll metabolic process | 12 out of 501 genes, 2.4% | 25 out of 2860 genes, 0.9% | 0.000418 |
| 17 | metabolic process | 418 out of 501 genes, 83.4% | 2229 out of 2860 genes, 77.9% | 0.000511 |
| 18 | chlorophyll biosynthetic process | 9 out of 501 genes, 1.8% | 16 out of 2860 genes, 0.6% | 0.000517 |
| 19 | response to stress | 94 out of 501 genes, 18.8% | 399 out of 2860 genes, 14.0% | 0.000568 |
| 20 | response to bacterium | 23 out of 501 genes, 4.6% | 68 out of 2860 genes, 2.4% | 0.000769 |
| 21 | response to cold | 21 out of 501 genes, 4.2% | 60 out of 2860 genes, 2.1% | 0.000779 |
| 22 | phospholipid biosynthetic process | 13 out of 501 genes, 2.6% | 30 out of 2860 genes, 1.0% | 0.000824 |
| 23 | response to hypoxia | 5 out of 501 genes, 1.0% | 6 out of 2860 genes, 0.2% | 0.000833 |
| 24 | response to decreased oxygen levels | 5 out of 501 genes, 1.0% | 6 out of 2860 genes, 0.2% | 0.000833 |
| 25 | response to oxygen levels | 5 out of 501 genes, 1.0% | 6 out of 2860 genes, 0.2% | 0.000833 |
| 26 | glycine decarboxylation via glycine cleavage system | 4 out of 501 genes, 0.8% | 4 out of 2860 genes, 0.1% | 0.000932 |
| 27 | organonitrogen compound catabolic process | 40 out of 501 genes, 8.0% | 143 out of 2860 genes, 5.0% | 0.000984 |
| 28 | cellular macromolecular complex assembly | 21 out of 501 genes, 4.2% | 64 out of 2860 genes, 2.2% | 0.002 |
| 29 | defense response to bacterium | 19 out of 501 genes, 3.8% | 56 out of 2860 genes, 2.0% | 0.002094 |
| 30 | pyruvate metabolic process | 10 out of 501 genes, 2.0% | 22 out of 2860 genes, 0.8% | 0.002151 |
| 31 | macromolecular complex assembly | 22 out of 501 genes, 4.4% | 69 out of 2860 genes, 2.4% | 0.002384 |
| 32 | small molecule metabolic process | 126 out of 501 genes, 25.1% | 583 out of 2860 genes, 20.4% | 0.002521 |
| 33 | electron transport chain | 15 out of 501 genes, 3.0% | 41 out of 2860 genes, 1.4% | 0.002649 |
| 34 | pigment metabolic process | 16 out of 501 genes, 3.2% | 45 out of 2860 genes, 1.6% | 0.002705 |
| 35 | alpha-amino acid metabolic process | 36 out of 501 genes, 7.2% | 132 out of 2860 genes, 4.6% | 0.002838 |
| 36 | secondary metabolic process | 18 out of 501 genes, 3.6% | 54 out of 2860 genes, 1.9% | 0.003399 |
| 37 | generation of precursor metabolites and energy | 40 out of 501 genes, 8.0% | 153 out of 2860 genes, 5.3% | 0.003897 |
| 38 | organic acid metabolic process | 76 out of 501 genes, 15.2% | 330 out of 2860 genes, 11.5% | 0.003991 |
| 39 | jasmonic acid biosynthetic process | 4 out of 501 genes, 0.8% | 5 out of 2860 genes, 0.2% | 0.004013 |
| 40 | porphyrin-containing compound metabolic process | 12 out of 501 genes, 2.4% | 31 out of 2860 genes, 1.1% | 0.004105 |
| 41 | tetrapyrrole metabolic process | 12 out of 501 genes, 2.4% | 31 out of 2860 genes, 1.1% | 0.004105 |
| 42 | carboxylic acid metabolic process | 75 out of 501 genes, 15.0% | 327 out of 2860 genes, 11.4% | 0.004751 |
| 43 | organophosphate biosynthetic process | 32 out of 501 genes, 6.4% | 118 out of 2860 genes, 4.1% | 0.005268 |
| 44 | ornithine metabolic process | 3 out of 501 genes, 0.6% | 3 out of 2860 genes, 0.1% | 0.005349 |
| 45 | photosynthetic electron transport in photosystem II | 3 out of 501 genes, 0.6% | 3 out of 2860 genes, 0.1% | 0.005349 |
| 46 | anthocyanin-containing compound metabolic process | 3 out of 501 genes, 0.6% | 3 out of 2860 genes, 0.1% | 0.005349 |
| 47 | cellular aldehyde metabolic process | 12 out of 501 genes, 2.4% | 32 out of 2860 genes, 1.1% | 0.005549 |
| 48 | oxoacid metabolic process | 75 out of 501 genes, 15.0% | 329 out of 2860 genes, 11.5% | 0.005605 |
| 49 | pigment biosynthetic process | 13 out of 501 genes, 2.6% | 36 out of 2860 genes, 1.3% | 0.00573 |
| 50 | isopentenyl diphosphate biosynthetic process | 9 out of 501 genes, 1.8% | 21 out of 2860 genes, 0.7% | 0.005795 |
| 51 | isopentenyl diphosphate biosynthetic process, mevalonate-independent pathway | 9 out of 501 genes, 1.8% | 21 out of 2860 genes, 0.7% | 0.005795 |
| 52 | glyceraldehyde-3-phosphate metabolic process | 9 out of 501 genes, 1.8% | 21 out of 2860 genes, 0.7% | 0.005795 |
| 53 | isopentenyl diphosphate metabolic process | 9 out of 501 genes, 1.8% | 21 out of 2860 genes, 0.7% | 0.005795 |
| 54 | cellular component assembly | 25 out of 501 genes, 5.0% | 87 out of 2860 genes, 3.0% | 0.005923 |
| 55 | carbohydrate metabolic process | 76 out of 501 genes, 15.2% | 335 out of 2860 genes, 11.7% | 0.006032 |
| 56 | macromolecular complex subunit organization | 22 out of 501 genes, 4.4% | 74 out of 2860 genes, 2.6% | 0.006197 |
| 57 | organonitrogen compound metabolic process | 102 out of 501 genes, 20.4% | 470 out of 2860 genes, 16.4% | 0.006255 |
| 58 | response to other organism | 34 out of 501 genes, 6.8% | 129 out of 2860 genes, 4.5% | 0.006623 |
| 59 | phospholipid metabolic process | 13 out of 501 genes, 2.6% | 37 out of 2860 genes, 1.3% | 0.007459 |
| 60 | cellular amino acid biosynthetic process | 30 out of 501 genes, 6.0% | 112 out of 2860 genes, 3.9% | 0.008227 |
| 61 | cellular amino acid metabolic process | 51 out of 501 genes, 10.2% | 213 out of 2860 genes, 7.4% | 0.008285 |
| 62 | nucleosome assembly | 9 out of 501 genes, 1.8% | 22 out of 2860 genes, 0.8% | 0.008318 |
| 63 | porphyrin-containing compound biosynthetic process | 9 out of 501 genes, 1.8% | 22 out of 2860 genes, 0.8% | 0.008318 |
| 64 | response to jasmonic acid stimulus | 9 out of 501 genes, 1.8% | 22 out of 2860 genes, 0.8% | 0.008318 |
| 65 | tetrapyrrole biosynthetic process | 9 out of 501 genes, 1.8% | 22 out of 2860 genes, 0.8% | 0.008318 |
| 66 | nucleosome organization | 9 out of 501 genes, 1.8% | 22 out of 2860 genes, 0.8% | 0.008318 |
| 67 | protein-DNA complex assembly | 9 out of 501 genes, 1.8% | 22 out of 2860 genes, 0.8% | 0.008318 |
| 68 | protein-DNA complex subunit organization | 9 out of 501 genes, 1.8% | 22 out of 2860 genes, 0.8% | 0.008318 |
| 69 | response to biotic stimulus | 34 out of 501 genes, 6.8% | 131 out of 2860 genes, 4.6% | 0.008525 |
| 70 | phenylpropanoid metabolic process | 12 out of 501 genes, 2.4% | 34 out of 2860 genes, 1.2% | 0.009626 |
| 71 | cellular component biogenesis | 32 out of 501 genes, 6.4% | 123 out of 2860 genes, 4.3% | 0.010186 |
| 72 | jasmonic acid metabolic process | 4 out of 501 genes, 0.8% | 6 out of 2860 genes, 0.2% | 0.010373 |
| 73 | flavonoid metabolic process | 4 out of 501 genes, 0.8% | 6 out of 2860 genes, 0.2% | 0.010373 |
| 74 | regulation of hydrogen peroxide metabolic process | 4 out of 501 genes, 0.8% | 6 out of 2860 genes, 0.2% | 0.010373 |
| 75 | regulation of reactive oxygen species metabolic process | 4 out of 501 genes, 0.8% | 6 out of 2860 genes, 0.2% | 0.010373 |
| 76 | response to abiotic stimulus | 60 out of 501 genes, 12.0% | 265 out of 2860 genes, 9.3% | 0.015092 |
| 77 | oligosaccharide metabolic process | 9 out of 501 genes, 1.8% | 24 out of 2860 genes, 0.8% | 0.015748 |
| 78 | chromatin assembly | 9 out of 501 genes, 1.8% | 24 out of 2860 genes, 0.8% | 0.015748 |
| 79 | response to oxidative stress | 24 out of 501 genes, 4.8% | 89 out of 2860 genes, 3.1% | 0.015826 |
| 80 | response to temperature stimulus | 27 out of 501 genes, 5.4% | 103 out of 2860 genes, 3.6% | 0.015881 |
| 81 | malate metabolic process | 6 out of 501 genes, 1.2% | 13 out of 2860 genes, 0.5% | 0.015883 |
| 82 | NADP metabolic process | 10 out of 501 genes, 2.0% | 28 out of 2860 genes, 1.0% | 0.016107 |
| 83 | organonitrogen compound biosynthetic process | 53 out of 501 genes, 10.6% | 231 out of 2860 genes, 8.1% | 0.017009 |
| 84 | aminoglycan catabolic process | 3 out of 501 genes, 0.6% | 4 out of 2860 genes, 0.1% | 0.018599 |
| 85 | chitin metabolic process | 3 out of 501 genes, 0.6% | 4 out of 2860 genes, 0.1% | 0.018599 |
| 86 | chitin catabolic process | 3 out of 501 genes, 0.6% | 4 out of 2860 genes, 0.1% | 0.018599 |
| 87 | phosphatidylinositol biosynthetic process | 3 out of 501 genes, 0.6% | 4 out of 2860 genes, 0.1% | 0.018599 |
| 88 | trichome branching | 3 out of 501 genes, 0.6% | 4 out of 2860 genes, 0.1% | 0.018599 |
| 89 | protein-chromophore linkage | 3 out of 501 genes, 0.6% | 4 out of 2860 genes, 0.1% | 0.018599 |
| 90 | amino sugar catabolic process | 3 out of 501 genes, 0.6% | 4 out of 2860 genes, 0.1% | 0.018599 |
| 91 | glucosamine-containing compound metabolic process | 3 out of 501 genes, 0.6% | 4 out of 2860 genes, 0.1% | 0.018599 |
| 92 | glucosamine-containing compound catabolic process | 3 out of 501 genes, 0.6% | 4 out of 2860 genes, 0.1% | 0.018599 |
| 93 | disaccharide metabolic process | 8 out of 501 genes, 1.6% | 21 out of 2860 genes, 0.7% | 0.020323 |
| 94 | unsaturated fatty acid biosynthetic process | 4 out of 501 genes, 0.8% | 7 out of 2860 genes, 0.2% | 0.020879 |
| 95 | systemic acquired resistance, salicylic acid mediated signaling pathway | 4 out of 501 genes, 0.8% | 7 out of 2860 genes, 0.2% | 0.020879 |
| 96 | unsaturated fatty acid metabolic process | 4 out of 501 genes, 0.8% | 7 out of 2860 genes, 0.2% | 0.020879 |
| 97 | nucleotide phosphorylation | 4 out of 501 genes, 0.8% | 7 out of 2860 genes, 0.2% | 0.020879 |
| 98 | DNA packaging | 9 out of 501 genes, 1.8% | 25 out of 2860 genes, 0.9% | 0.020898 |
| 99 | dicarboxylic acid metabolic process | 13 out of 501 genes, 2.6% | 42 out of 2860 genes, 1.5% | 0.023019 |
| 100 | alpha-amino acid biosynthetic process | 23 out of 501 genes, 4.6% | 88 out of 2860 genes, 3.1% | 0.025798 |
| 101 | response to stimulus | 145 out of 501 genes, 28.9% | 727 out of 2860 genes, 25.4% | 0.027295 |
| 102 | defense response | 26 out of 501 genes, 5.2% | 103 out of 2860 genes, 3.6% | 0.028312 |
| 103 | cellular modified amino acid biosynthetic process | 5 out of 501 genes, 1.0% | 11 out of 2860 genes, 0.4% | 0.029686 |
| 104 | cellular catabolic process | 52 out of 501 genes, 10.4% | 233 out of 2860 genes, 8.1% | 0.029857 |
| 105 | glycosylceramide metabolic process | 2 out of 501 genes, 0.4% | 2 out of 2860 genes, 0.1% | 0.030636 |
| 106 | glycosphingolipid metabolic process | 2 out of 501 genes, 0.4% | 2 out of 2860 genes, 0.1% | 0.030636 |
| 107 | threonine biosynthetic process | 2 out of 501 genes, 0.4% | 2 out of 2860 genes, 0.1% | 0.030636 |
| 108 | response to herbicide | 2 out of 501 genes, 0.4% | 2 out of 2860 genes, 0.1% | 0.030636 |
| 109 | anthocyanin-containing compound biosynthetic process | 2 out of 501 genes, 0.4% | 2 out of 2860 genes, 0.1% | 0.030636 |
| 110 | glycoside metabolic process | 2 out of 501 genes, 0.4% | 2 out of 2860 genes, 0.1% | 0.030636 |
| 111 | glycoside catabolic process | 2 out of 501 genes, 0.4% | 2 out of 2860 genes, 0.1% | 0.030636 |
| 112 | glycolipid catabolic process | 2 out of 501 genes, 0.4% | 2 out of 2860 genes, 0.1% | 0.030636 |
| 113 | sphingolipid catabolic process | 2 out of 501 genes, 0.4% | 2 out of 2860 genes, 0.1% | 0.030636 |
| 114 | membrane lipid catabolic process | 2 out of 501 genes, 0.4% | 2 out of 2860 genes, 0.1% | 0.030636 |
| 115 | glycosylceramide catabolic process | 2 out of 501 genes, 0.4% | 2 out of 2860 genes, 0.1% | 0.030636 |
| 116 | glycosphingolipid catabolic process | 2 out of 501 genes, 0.4% | 2 out of 2860 genes, 0.1% | 0.030636 |
| 117 | glyoxylate metabolic process | 2 out of 501 genes, 0.4% | 2 out of 2860 genes, 0.1% | 0.030636 |
| 118 | ceramide catabolic process | 2 out of 501 genes, 0.4% | 2 out of 2860 genes, 0.1% | 0.030636 |
| 119 | immune response | 11 out of 501 genes, 2.2% | 35 out of 2860 genes, 1.2% | 0.031571 |
| 120 | innate immune response | 11 out of 501 genes, 2.2% | 35 out of 2860 genes, 1.2% | 0.031571 |
| 121 | response to salt stress | 23 out of 501 genes, 4.6% | 90 out of 2860 genes, 3.1% | 0.033094 |
| 122 | hydrogen transport | 10 out of 501 genes, 2.0% | 31 out of 2860 genes, 1.1% | 0.033241 |
| 123 | proton transport | 10 out of 501 genes, 2.0% | 31 out of 2860 genes, 1.1% | 0.033241 |
| 124 | branched-chain amino acid metabolic process | 6 out of 501 genes, 1.2% | 15 out of 2860 genes, 0.5% | 0.033905 |
| 125 | glutamine family amino acid biosynthetic process | 6 out of 501 genes, 1.2% | 15 out of 2860 genes, 0.5% | 0.033905 |
| 126 | pentose-phosphate shunt | 9 out of 501 genes, 1.8% | 27 out of 2860 genes, 0.9% | 0.034627 |
| 127 | NADPH regeneration | 9 out of 501 genes, 1.8% | 27 out of 2860 genes, 0.9% | 0.034627 |
| 128 | cellular modified amino acid metabolic process | 7 out of 501 genes, 1.4% | 19 out of 2860 genes, 0.7% | 0.03547 |
| 129 | cation homeostasis | 7 out of 501 genes, 1.4% | 19 out of 2860 genes, 0.7% | 0.03547 |
| 130 | response to toxic substance | 4 out of 501 genes, 0.8% | 8 out of 2860 genes, 0.3% | 0.036061 |
| 131 | photosynthetic electron transport in photosystem I | 4 out of 501 genes, 0.8% | 8 out of 2860 genes, 0.3% | 0.036061 |
| 132 | salicylic acid mediated signaling pathway | 4 out of 501 genes, 0.8% | 8 out of 2860 genes, 0.3% | 0.036061 |
| 133 | response to chitin | 4 out of 501 genes, 0.8% | 8 out of 2860 genes, 0.3% | 0.036061 |
| 134 | photosystem II assembly | 4 out of 501 genes, 0.8% | 8 out of 2860 genes, 0.3% | 0.036061 |
| 135 | response to organic nitrogen | 4 out of 501 genes, 0.8% | 8 out of 2860 genes, 0.3% | 0.036061 |
| 136 | carbon fixation | 4 out of 501 genes, 0.8% | 8 out of 2860 genes, 0.3% | 0.036061 |
| 137 | regulation of protein dephosphorylation | 4 out of 501 genes, 0.8% | 8 out of 2860 genes, 0.3% | 0.036061 |
| 138 | cellular response to salicylic acid stimulus | 4 out of 501 genes, 0.8% | 8 out of 2860 genes, 0.3% | 0.036061 |
| 139 | divalent inorganic cation homeostasis | 4 out of 501 genes, 0.8% | 8 out of 2860 genes, 0.3% | 0.036061 |
| 140 | organic acid biosynthetic process | 37 out of 501 genes, 7.4% | 160 out of 2860 genes, 5.6% | 0.038105 |
| 141 | carboxylic acid biosynthetic process | 37 out of 501 genes, 7.4% | 160 out of 2860 genes, 5.6% | 0.038105 |
| 142 | multi-organism process | 36 out of 501 genes, 7.2% | 155 out of 2860 genes, 5.4% | 0.038134 |
| 143 | aminoglycan metabolic process | 3 out of 501 genes, 0.6% | 5 out of 2860 genes, 0.2% | 0.040477 |
| 144 | amino sugar metabolic process | 3 out of 501 genes, 0.6% | 5 out of 2860 genes, 0.2% | 0.040477 |
| 145 | DNA replication initiation | 3 out of 501 genes, 0.6% | 5 out of 2860 genes, 0.2% | 0.040477 |
| 146 | arginine biosynthetic process | 3 out of 501 genes, 0.6% | 5 out of 2860 genes, 0.2% | 0.040477 |
| 147 | superoxide metabolic process | 3 out of 501 genes, 0.6% | 5 out of 2860 genes, 0.2% | 0.040477 |
| 148 | flavonoid biosynthetic process | 3 out of 501 genes, 0.6% | 5 out of 2860 genes, 0.2% | 0.040477 |
| 149 | defense response, incompatible interaction | 10 out of 501 genes, 2.0% | 32 out of 2860 genes, 1.1% | 0.041055 |
| 150 | pyridine nucleotide metabolic process | 10 out of 501 genes, 2.0% | 32 out of 2860 genes, 1.1% | 0.041055 |
| 151 | nicotinamide nucleotide metabolic process | 10 out of 501 genes, 2.0% | 32 out of 2860 genes, 1.1% | 0.041055 |
| 152 | purine nucleoside biosynthetic process | 14 out of 501 genes, 2.8% | 50 out of 2860 genes, 1.7% | 0.043226 |
| 153 | purine ribonucleoside biosynthetic process | 14 out of 501 genes, 2.8% | 50 out of 2860 genes, 1.7% | 0.043226 |
| 154 | carbohydrate derivative metabolic process | 51 out of 501 genes, 10.2% | 233 out of 2860 genes, 8.1% | 0.043421 |
| 155 | arginine metabolic process | 5 out of 501 genes, 1.0% | 12 out of 2860 genes, 0.4% | 0.043743 |
| 156 | one-carbon metabolic process | 5 out of 501 genes, 1.0% | 12 out of 2860 genes, 0.4% | 0.043743 |
| 157 | nucleobase-containing small molecule metabolic process | 47 out of 501 genes, 9.4% | 213 out of 2860 genes, 7.4% | 0.045344 |
| 158 | systemic acquired resistance | 8 out of 501 genes, 1.6% | 24 out of 2860 genes, 0.8% | 0.045371 |
| 159 | ion homeostasis | 8 out of 501 genes, 1.6% | 24 out of 2860 genes, 0.8% | 0.045371 |
| 160 | cofactor biosynthetic process | 15 out of 501 genes, 3.0% | 55 out of 2860 genes, 1.9% | 0.046117 |
| 161 | immune system process | 11 out of 501 genes, 2.2% | 37 out of 2860 genes, 1.3% | 0.046533 |
| 162 | oxidoreduction coenzyme metabolic process | 11 out of 501 genes, 2.2% | 37 out of 2860 genes, 1.3% | 0.046533 |
| 163 | serine family amino acid metabolic process | 11 out of 501 genes, 2.2% | 37 out of 2860 genes, 1.3% | 0.046533 |
| 164 | heterocycle biosynthetic process | 40 out of 501 genes, 8.0% | 178 out of 2860 genes, 6.2% | 0.048193 |
| 165 | cellular biosynthetic process | 135 out of 501 genes, 26.9% | 685 out of 2860 genes, 24.0% | 0.048347 |
|  |  | **S2-VS-S1_ Cellular Component** |  |  |
|  | **Gene Ontology term** | **Cluster frequency** | **Protein frequency of use** | **P-value** |
| 1 | chloroplast | 214 out of 713 genes, 30.0% | 529 out of 2292 genes, 23.1% | 1.21E-07 |
| 2 | thylakoid | 67 out of 713 genes, 9.4% | 128 out of 2292 genes, 5.6% | 2.20E-07 |
| 3 | plastid | 237 out of 713 genes, 33.2% | 612 out of 2292 genes, 26.7% | 1.65E-06 |
| 4 | plastid stroma | 73 out of 713 genes, 10.2% | 149 out of 2292 genes, 6.5% | 1.82E-06 |
| 5 | chloroplast stroma | 71 out of 713 genes, 10.0% | 146 out of 2292 genes, 6.4% | 3.62E-06 |
| 6 | photosynthetic membrane | 48 out of 713 genes, 6.7% | 90 out of 2292 genes, 3.9% | 6.51E-06 |
| 7 | thylakoid part | 50 out of 713 genes, 7.0% | 95 out of 2292 genes, 4.1% | 6.85E-06 |
| 8 | intracellular part | 600 out of 713 genes, 84.2% | 1804 out of 2292 genes, 78.7% | 8.41E-06 |
| 9 | chloroplast part | 111 out of 713 genes, 15.6% | 258 out of 2292 genes, 11.3% | 1.22E-05 |
| 10 | cytoplasm | 539 out of 713 genes, 75.6% | 1596 out of 2292 genes, 69.6% | 1.53E-05 |
| 11 | plastid part | 112 out of 713 genes, 15.7% | 262 out of 2292 genes, 11.4% | 1.59E-05 |
| 12 | photosystem | 15 out of 713 genes, 2.1% | 20 out of 2292 genes, 0.9% | 6.39E-05 |
| 13 | extracellular region | 69 out of 713 genes, 9.7% | 155 out of 2292 genes, 6.8% | 0.000191 |
| 14 | thylakoid membrane | 40 out of 713 genes, 5.6% | 80 out of 2292 genes, 3.5% | 0.000257 |
| 15 | organelle part | 222 out of 713 genes, 31.1% | 605 out of 2292 genes, 26.4% | 0.000362 |
| 16 | intracellular organelle part | 222 out of 713 genes, 31.1% | 605 out of 2292 genes, 26.4% | 0.000362 |
| 17 | cytoplasmic part | 458 out of 713 genes, 64.2% | 1358 out of 2292 genes, 59.2% | 0.000617 |
| 18 | photosystem II | 10 out of 713 genes, 1.4% | 13 out of 2292 genes, 0.6% | 0.00087 |
| 19 | plastoglobule | 11 out of 713 genes, 1.5% | 15 out of 2292 genes, 0.7% | 0.000913 |
| 20 | chloroplast thylakoid membrane | 36 out of 713 genes, 5.0% | 74 out of 2292 genes, 3.2% | 0.001008 |
| 21 | plastid thylakoid membrane | 36 out of 713 genes, 5.0% | 74 out of 2292 genes, 3.2% | 0.001008 |
| 22 | organelle subcompartment | 40 out of 713 genes, 5.6% | 85 out of 2292 genes, 3.7% | 0.001228 |
| 23 | membrane-bounded organelle | 466 out of 713 genes, 65.4% | 1393 out of 2292 genes, 60.8% | 0.001423 |
| 24 | intracellular membrane-bounded organelle | 466 out of 713 genes, 65.4% | 1393 out of 2292 genes, 60.8% | 0.001423 |
| 25 | apoplast | 39 out of 713 genes, 5.5% | 83 out of 2292 genes, 3.6% | 0.001462 |
| 26 | proteasome complex | 18 out of 713 genes, 2.5% | 31 out of 2292 genes, 1.4% | 0.001598 |
| 27 | chloroplast thylakoid | 39 out of 713 genes, 5.5% | 84 out of 2292 genes, 3.7% | 0.001933 |
| 28 | plastid thylakoid | 39 out of 713 genes, 5.5% | 84 out of 2292 genes, 3.7% | 0.001933 |
| 29 | macromolecular complex | 173 out of 713 genes, 24.3% | 473 out of 2292 genes, 20.6% | 0.002541 |
| 30 | intracellular | 608 out of 713 genes, 85.3% | 1877 out of 2292 genes, 81.9% | 0.002548 |
| 31 | nucleolus | 45 out of 713 genes, 6.3% | 106 out of 2292 genes, 4.6% | 0.007638 |
| 32 | vacuole | 68 out of 713 genes, 9.5% | 171 out of 2292 genes, 7.5% | 0.007812 |
| 33 | organelle | 492 out of 713 genes, 69.0% | 1499 out of 2292 genes, 65.4% | 0.008224 |
| 34 | intracellular organelle | 492 out of 713 genes, 69.0% | 1499 out of 2292 genes, 65.4% | 0.008224 |
| 35 | nucleosome | 12 out of 713 genes, 1.7% | 21 out of 2292 genes, 0.9% | 0.01159 |
| 36 | protein-DNA complex | 12 out of 713 genes, 1.7% | 21 out of 2292 genes, 0.9% | 0.01159 |
| 37 | cell wall | 66 out of 713 genes, 9.3% | 168 out of 2292 genes, 7.3% | 0.011962 |
| 38 | external encapsulating structure | 66 out of 713 genes, 9.3% | 168 out of 2292 genes, 7.3% | 0.011962 |
| 39 | photosystem I | 6 out of 713 genes, 0.8% | 8 out of 2292 genes, 0.3% | 0.013542 |
| 40 | organelle lumen | 51 out of 713 genes, 7.2% | 130 out of 2292 genes, 5.7% | 0.026435 |
| 41 | intracellular organelle lumen | 51 out of 713 genes, 7.2% | 130 out of 2292 genes, 5.7% | 0.026435 |
| 42 | large ribosomal subunit | 16 out of 713 genes, 2.2% | 33 out of 2292 genes, 1.4% | 0.026561 |
| 43 | thylakoid lumen | 10 out of 713 genes, 1.4% | 18 out of 2292 genes, 0.8% | 0.026572 |
| 44 | membrane-enclosed lumen | 52 out of 713 genes, 7.3% | 133 out of 2292 genes, 5.8% | 0.026894 |
| 45 | cell periphery | 184 out of 713 genes, 25.8% | 532 out of 2292 genes, 23.2% | 0.02773 |
| 46 | photosystem I reaction center | 3 out of 713 genes, 0.4% | 3 out of 2292 genes, 0.1% | 0.030017 |
| 47 | light-harvesting complex | 3 out of 713 genes, 0.4% | 3 out of 2292 genes, 0.1% | 0.030017 |
| 48 | proteasome core complex | 9 out of 713 genes, 1.3% | 16 out of 2292 genes, 0.7% | 0.031946 |
| 49 | Golgi-associated vesicle | 5 out of 713 genes, 0.7% | 7 out of 2292 genes, 0.3% | 0.033471 |
| 50 | oxygen evolving complex | 4 out of 713 genes, 0.6% | 5 out of 2292 genes, 0.2% | 0.035012 |
| 51 | nuclear part | 53 out of 713 genes, 7.4% | 138 out of 2292 genes, 6.0% | 0.036353 |
| 52 | cytosolic large ribosomal subunit | 14 out of 713 genes, 2.0% | 29 out of 2292 genes, 1.3% | 0.038565 |
| 53 | chromatin | 12 out of 713 genes, 1.7% | 24 out of 2292 genes, 1.0% | 0.040385 |
| 54 | chromosomal part | 12 out of 713 genes, 1.7% | 24 out of 2292 genes, 1.0% | 0.040385 |
| 55 | nuclear lumen | 47 out of 713 genes, 6.6% | 122 out of 2292 genes, 5.3% | 0.044642 |
|  |  | **S2-VS-S1_ Molecular Function** |  |  |
|  | **Gene Ontology term** | **Cluster frequency** | **Protein frequency of use** | **P-value** |
| 1 | hydrolase activity, acting on glycosyl bonds | 70 out of 1035 genes, 6.8% | 159 out of 3345 genes, 4.8% | 0.000253 |
| 2 | magnesium ion binding | 27 out of 1035 genes, 2.6% | 53 out of 3345 genes, 1.6% | 0.001733 |
| 3 | transaminase activity | 16 out of 1035 genes, 1.5% | 27 out of 3345 genes, 0.8% | 0.002081 |
| 4 | transferase activity, transferring nitrogenous groups | 16 out of 1035 genes, 1.5% | 27 out of 3345 genes, 0.8% | 0.002081 |
| 5 | lyase activity | 54 out of 1035 genes, 5.2% | 125 out of 3345 genes, 3.7% | 0.002155 |
| 6 | antioxidant activity | 29 out of 1035 genes, 2.8% | 59 out of 3345 genes, 1.8% | 0.002407 |
| 7 | tetrapyrrole binding | 30 out of 1035 genes, 2.9% | 62 out of 3345 genes, 1.9% | 0.002771 |
| 8 | carbon-oxygen lyase activity | 22 out of 1035 genes, 2.1% | 43 out of 3345 genes, 1.3% | 0.004251 |
| 9 | aldehyde-lyase activity | 6 out of 1035 genes, 0.6% | 7 out of 3345 genes, 0.2% | 0.004475 |
| 10 | hydrolase activity, hydrolyzing O-glycosyl compounds | 52 out of 1035 genes, 5.0% | 125 out of 3345 genes, 3.7% | 0.006602 |
| 11 | disulfide oxidoreductase activity | 16 out of 1035 genes, 1.5% | 30 out of 3345 genes, 0.9% | 0.00856 |
| 12 | oxidoreductase activity, acting on a sulfur group of donors | 20 out of 1035 genes, 1.9% | 40 out of 3345 genes, 1.2% | 0.008731 |
| 13 | carboxylic acid binding | 10 out of 1035 genes, 1.0% | 16 out of 3345 genes, 0.5% | 0.008914 |
| 14 | ribokinase activity | 4 out of 1035 genes, 0.4% | 4 out of 3345 genes, 0.1% | 0.009129 |
| 15 | ribosome binding | 4 out of 1035 genes, 0.4% | 4 out of 3345 genes, 0.1% | 0.009129 |
| 16 | cofactor binding | 78 out of 1035 genes, 7.5% | 202 out of 3345 genes, 6.0% | 0.010146 |
| 17 | serine-type carboxypeptidase activity | 9 out of 1035 genes, 0.9% | 14 out of 3345 genes, 0.4% | 0.010198 |
| 18 | heme binding | 27 out of 1035 genes, 2.6% | 59 out of 3345 genes, 1.8% | 0.011184 |
| 19 | peroxidase activity | 22 out of 1035 genes, 2.1% | 46 out of 3345 genes, 1.4% | 0.011601 |
| 20 | oxidoreductase activity, acting on peroxide as acceptor | 22 out of 1035 genes, 2.1% | 46 out of 3345 genes, 1.4% | 0.011601 |
| 21 | protein disulfide oxidoreductase activity | 14 out of 1035 genes, 1.4% | 26 out of 3345 genes, 0.8% | 0.012318 |
| 22 | transferase activity, transferring sulfur-containing groups | 6 out of 1035 genes, 0.6% | 8 out of 3345 genes, 0.2% | 0.013206 |
| 23 | carboxypeptidase activity | 11 out of 1035 genes, 1.1% | 19 out of 3345 genes, 0.6% | 0.013267 |
| 24 | peptidase activity | 61 out of 1035 genes, 5.9% | 156 out of 3345 genes, 4.7% | 0.016277 |
| 25 | threonine-type endopeptidase activity | 9 out of 1035 genes, 0.9% | 15 out of 3345 genes, 0.4% | 0.018582 |
| 26 | threonine-type peptidase activity | 9 out of 1035 genes, 0.9% | 15 out of 3345 genes, 0.4% | 0.018582 |
| 27 | 2-alkenal reductase [NAD(P)] activity | 10 out of 1035 genes, 1.0% | 18 out of 3345 genes, 0.5% | 0.02574 |
| 28 | endopeptidase activity | 29 out of 1035 genes, 2.8% | 68 out of 3345 genes, 2.0% | 0.026194 |
| 29 | peptidase activity, acting on L-amino acid peptides | 50 out of 1035 genes, 4.8% | 128 out of 3345 genes, 3.8% | 0.028528 |
| 30 | 3-hydroxyacyl-CoA dehydrogenase activity | 3 out of 1035 genes, 0.3% | 3 out of 3345 genes, 0.1% | 0.029564 |
| 31 | dihydrolipoyl dehydrogenase activity | 3 out of 1035 genes, 0.3% | 3 out of 3345 genes, 0.1% | 0.029564 |
| 32 | methionine adenosyltransferase activity | 3 out of 1035 genes, 0.3% | 3 out of 3345 genes, 0.1% | 0.029564 |
| 33 | ribose-5-phosphate isomerase activity | 3 out of 1035 genes, 0.3% | 3 out of 3345 genes, 0.1% | 0.029564 |
| 34 | asparagine-tRNA ligase activity | 3 out of 1035 genes, 0.3% | 3 out of 3345 genes, 0.1% | 0.029564 |
| 35 | hexosaminidase activity | 3 out of 1035 genes, 0.3% | 3 out of 3345 genes, 0.1% | 0.029564 |
| 36 | chlorophyll binding | 3 out of 1035 genes, 0.3% | 3 out of 3345 genes, 0.1% | 0.029564 |
| 37 | L-ascorbic acid binding | 3 out of 1035 genes, 0.3% | 3 out of 3345 genes, 0.1% | 0.029564 |
| 38 | monosaccharide binding | 3 out of 1035 genes, 0.3% | 3 out of 3345 genes, 0.1% | 0.029564 |
| 39 | serine-type exopeptidase activity | 9 out of 1035 genes, 0.9% | 16 out of 3345 genes, 0.5% | 0.031012 |
| 40 | copper ion binding | 25 out of 1035 genes, 2.4% | 58 out of 3345 genes, 1.7% | 0.032654 |
| 41 | sulfurtransferase activity | 5 out of 1035 genes, 0.5% | 7 out of 3345 genes, 0.2% | 0.03277 |
| 42 | carbon-oxygen lyase activity, acting on phosphates | 5 out of 1035 genes, 0.5% | 7 out of 3345 genes, 0.2% | 0.03277 |
| 43 | carbohydrate phosphatase activity | 5 out of 1035 genes, 0.5% | 7 out of 3345 genes, 0.2% | 0.03277 |
| 44 | sugar-phosphatase activity | 5 out of 1035 genes, 0.5% | 7 out of 3345 genes, 0.2% | 0.03277 |
| 45 | fructose-bisphosphate aldolase activity | 4 out of 1035 genes, 0.4% | 5 out of 3345 genes, 0.1% | 0.034378 |
| 46 | amino acid kinase activity | 4 out of 1035 genes, 0.4% | 5 out of 3345 genes, 0.1% | 0.034378 |
| 47 | fructose 1,6-bisphosphate 1-phosphatase activity | 4 out of 1035 genes, 0.4% | 5 out of 3345 genes, 0.1% | 0.034378 |
| 48 | ribonucleoprotein complex binding | 4 out of 1035 genes, 0.4% | 5 out of 3345 genes, 0.1% | 0.034378 |
| 49 | pyridoxal phosphate binding | 24 out of 1035 genes, 2.3% | 56 out of 3345 genes, 1.7% | 0.038531 |
| 50 | cation binding | 202 out of 1035 genes, 19.5% | 593 out of 3345 genes, 17.7% | 0.039494 |
| 51 | oxidoreductase activity, acting on the aldehyde or oxo group of donors, NAD or NADP as acceptor | 15 out of 1035 genes, 1.4% | 32 out of 3345 genes, 1.0% | 0.04183 |
| 52 | acid-thiol ligase activity | 7 out of 1035 genes, 0.7% | 12 out of 3345 genes, 0.4% | 0.045096 |
|  |  | **S2-VS-S1_ Biological Process** |  |  |
|  | **Gene Ontology term** | **Cluster frequency** | **Protein frequency of use** | **P-value** |
| 1 | photosynthesis | 41 out of 908 genes, 4.5% | 69 out of 2860 genes, 2.4% | 1.51E-06 |
| 2 | single-organism metabolic process | 367 out of 908 genes, 40.4% | 981 out of 2860 genes, 34.3% | 1.82E-06 |
| 3 | cellular amino acid metabolic process | 99 out of 908 genes, 10.9% | 213 out of 2860 genes, 7.4% | 2.13E-06 |
| 4 | alpha-amino acid metabolic process | 67 out of 908 genes, 7.4% | 132 out of 2860 genes, 4.6% | 2.62E-06 |
| 5 | photosynthesis, light reaction | 28 out of 908 genes, 3.1% | 43 out of 2860 genes, 1.5% | 6.09E-06 |
| 6 | small molecule metabolic process | 230 out of 908 genes, 25.3% | 583 out of 2860 genes, 20.4% | 6.16E-06 |
| 7 | organic acid metabolic process | 139 out of 908 genes, 15.3% | 330 out of 2860 genes, 11.5% | 1.59E-05 |
| 8 | oxoacid metabolic process | 138 out of 908 genes, 15.2% | 329 out of 2860 genes, 11.5% | 2.22E-05 |
| 9 | carboxylic acid metabolic process | 137 out of 908 genes, 15.1% | 327 out of 2860 genes, 11.4% | 2.57E-05 |
| 10 | glutamine family amino acid metabolic process | 20 out of 908 genes, 2.2% | 30 out of 2860 genes, 1.0% | 8.32E-05 |
| 11 | response to stress | 159 out of 908 genes, 17.5% | 399 out of 2860 genes, 14.0% | 0.000139 |
| 12 | cellular modified amino acid metabolic process | 14 out of 908 genes, 1.5% | 19 out of 2860 genes, 0.7% | 0.000204 |
| 13 | generation of precursor metabolites and energy | 68 out of 908 genes, 7.5% | 153 out of 2860 genes, 5.3% | 0.000483 |
| 14 | defense response to bacterium | 30 out of 908 genes, 3.3% | 56 out of 2860 genes, 2.0% | 0.000516 |
| 15 | dicarboxylic acid metabolic process | 24 out of 908 genes, 2.6% | 42 out of 2860 genes, 1.5% | 0.000541 |
| 16 | oxidation-reduction process | 174 out of 908 genes, 19.2% | 453 out of 2860 genes, 15.8% | 0.000625 |
| 17 | cellular amino acid biosynthetic process | 52 out of 908 genes, 5.7% | 112 out of 2860 genes, 3.9% | 0.000651 |
| 18 | homeostatic process | 35 out of 908 genes, 3.9% | 69 out of 2860 genes, 2.4% | 0.000702 |
| 19 | cellular homeostasis | 30 out of 908 genes, 3.3% | 58 out of 2860 genes, 2.0% | 0.001113 |
| 20 | response to bacterium | 34 out of 908 genes, 3.7% | 68 out of 2860 genes, 2.4% | 0.00116 |
| 21 | organonitrogen compound metabolic process | 177 out of 908 genes, 19.5% | 470 out of 2860 genes, 16.4% | 0.00171 |
| 22 | metabolic process | 738 out of 908 genes, 81.3% | 2229 out of 2860 genes, 77.9% | 0.001767 |
| 23 | carbohydrate metabolic process | 130 out of 908 genes, 14.3% | 335 out of 2860 genes, 11.7% | 0.002159 |
| 24 | sulfur amino acid metabolic process | 20 out of 908 genes, 2.2% | 36 out of 2860 genes, 1.3% | 0.002496 |
| 25 | photosynthesis, light harvesting | 11 out of 908 genes, 1.2% | 16 out of 2860 genes, 0.6% | 0.002549 |
| 26 | arginine metabolic process | 9 out of 908 genes, 1.0% | 12 out of 2860 genes, 0.4% | 0.002589 |
| 27 | photosynthetic electron transport chain | 10 out of 908 genes, 1.1% | 14 out of 2860 genes, 0.5% | 0.00263 |
| 28 | electron transport chain | 22 out of 908 genes, 2.4% | 41 out of 2860 genes, 1.4% | 0.002781 |
| 29 | single-organism biosynthetic process | 90 out of 908 genes, 9.9% | 223 out of 2860 genes, 7.8% | 0.002912 |
| 30 | response to other organism | 56 out of 908 genes, 6.2% | 129 out of 2860 genes, 4.5% | 0.002921 |
| 31 | organic acid catabolic process | 26 out of 908 genes, 2.9% | 51 out of 2860 genes, 1.8% | 0.003073 |
| 32 | small molecule catabolic process | 26 out of 908 genes, 2.9% | 51 out of 2860 genes, 1.8% | 0.003073 |
| 33 | single-organism catabolic process | 26 out of 908 genes, 2.9% | 51 out of 2860 genes, 1.8% | 0.003073 |
| 34 | carboxylic acid catabolic process | 26 out of 908 genes, 2.9% | 51 out of 2860 genes, 1.8% | 0.003073 |
| 35 | jasmonic acid biosynthetic process | 5 out of 908 genes, 0.6% | 5 out of 2860 genes, 0.2% | 0.003201 |
| 36 | S-adenosylmethionine metabolic process | 5 out of 908 genes, 0.6% | 5 out of 2860 genes, 0.2% | 0.003201 |
| 37 | macromolecular complex subunit organization | 35 out of 908 genes, 3.9% | 74 out of 2860 genes, 2.6% | 0.003341 |
| 38 | cellular amino acid catabolic process | 18 out of 908 genes, 2.0% | 32 out of 2860 genes, 1.1% | 0.003402 |
| 39 | macromolecular complex assembly | 33 out of 908 genes, 3.6% | 69 out of 2860 genes, 2.4% | 0.003473 |
| 40 | defense response | 46 out of 908 genes, 5.1% | 103 out of 2860 genes, 3.6% | 0.003497 |
| 41 | alpha-amino acid biosynthetic process | 40 out of 908 genes, 4.4% | 88 out of 2860 genes, 3.1% | 0.004317 |
| 42 | response to biotic stimulus | 56 out of 908 genes, 6.2% | 131 out of 2860 genes, 4.6% | 0.004391 |
| 43 | cellular component biogenesis | 53 out of 908 genes, 5.8% | 123 out of 2860 genes, 4.3% | 0.004532 |
| 44 | response to oxygen-containing compound | 58 out of 908 genes, 6.4% | 137 out of 2860 genes, 4.8% | 0.004874 |
| 45 | response to oxidative stress | 40 out of 908 genes, 4.4% | 89 out of 2860 genes, 3.1% | 0.005501 |
| 46 | glycerol ether metabolic process | 10 out of 908 genes, 1.1% | 15 out of 2860 genes, 0.5% | 0.005659 |
| 47 | glutamine family amino acid biosynthetic process | 10 out of 908 genes, 1.1% | 15 out of 2860 genes, 0.5% | 0.005659 |
| 48 | ether metabolic process | 10 out of 908 genes, 1.1% | 15 out of 2860 genes, 0.5% | 0.005659 |
| 49 | cell redox homeostasis | 20 out of 908 genes, 2.2% | 38 out of 2860 genes, 1.3% | 0.005734 |
| 50 | small molecule biosynthetic process | 81 out of 908 genes, 8.9% | 202 out of 2860 genes, 7.1% | 0.005739 |
| 51 | organic acid biosynthetic process | 66 out of 908 genes, 7.3% | 160 out of 2860 genes, 5.6% | 0.005764 |
| 52 | carboxylic acid biosynthetic process | 66 out of 908 genes, 7.3% | 160 out of 2860 genes, 5.6% | 0.005764 |
| 53 | alpha-amino acid catabolic process | 17 out of 908 genes, 1.9% | 31 out of 2860 genes, 1.1% | 0.006228 |
| 54 | glutamate metabolic process | 8 out of 908 genes, 0.9% | 11 out of 2860 genes, 0.4% | 0.006245 |
| 55 | cellular component assembly | 39 out of 908 genes, 4.3% | 87 out of 2860 genes, 3.0% | 0.00642 |
| 56 | coenzyme metabolic process | 34 out of 908 genes, 3.7% | 74 out of 2860 genes, 2.6% | 0.006715 |
| 57 | cellular macromolecular complex assembly | 30 out of 908 genes, 3.3% | 64 out of 2860 genes, 2.2% | 0.007501 |
| 58 | phenylpropanoid metabolic process | 18 out of 908 genes, 2.0% | 34 out of 2860 genes, 1.2% | 0.007986 |
| 59 | oxidoreduction coenzyme metabolic process | 19 out of 908 genes, 2.1% | 37 out of 2860 genes, 1.3% | 0.009853 |
| 60 | serine family amino acid metabolic process | 19 out of 908 genes, 2.1% | 37 out of 2860 genes, 1.3% | 0.009853 |
| 61 | D-ribose metabolic process | 4 out of 908 genes, 0.4% | 4 out of 2860 genes, 0.1% | 0.010114 |
| 62 | plant-type ovary development | 4 out of 908 genes, 0.4% | 4 out of 2860 genes, 0.1% | 0.010114 |
| 63 | ovule development | 4 out of 908 genes, 0.4% | 4 out of 2860 genes, 0.1% | 0.010114 |
| 64 | multi-organism process | 63 out of 908 genes, 6.9% | 155 out of 2860 genes, 5.4% | 0.010132 |
| 65 | response to temperature stimulus | 44 out of 908 genes, 4.8% | 103 out of 2860 genes, 3.6% | 0.011155 |
| 66 | response to salt stress | 39 out of 908 genes, 4.3% | 90 out of 2860 genes, 3.1% | 0.01254 |
| 67 | NADP metabolic process | 15 out of 908 genes, 1.7% | 28 out of 2860 genes, 1.0% | 0.013175 |
| 68 | disaccharide metabolic process | 12 out of 908 genes, 1.3% | 21 out of 2860 genes, 0.7% | 0.013862 |
| 69 | glutamine metabolic process | 5 out of 908 genes, 0.6% | 6 out of 2860 genes, 0.2% | 0.014145 |
| 70 | jasmonic acid metabolic process | 5 out of 908 genes, 0.6% | 6 out of 2860 genes, 0.2% | 0.014145 |
| 71 | sulfur compound metabolic process | 29 out of 908 genes, 3.2% | 64 out of 2860 genes, 2.2% | 0.014838 |
| 72 | response to osmotic stress | 42 out of 908 genes, 4.6% | 99 out of 2860 genes, 3.5% | 0.014869 |
| 73 | photosystem II assembly | 6 out of 908 genes, 0.7% | 8 out of 2860 genes, 0.3% | 0.015113 |
| 74 | carbon fixation | 6 out of 908 genes, 0.7% | 8 out of 2860 genes, 0.3% | 0.015113 |
| 75 | dicarboxylic acid biosynthetic process | 6 out of 908 genes, 0.7% | 8 out of 2860 genes, 0.3% | 0.015113 |
| 76 | cation homeostasis | 11 out of 908 genes, 1.2% | 19 out of 2860 genes, 0.7% | 0.016224 |
| 77 | response to metal ion | 58 out of 908 genes, 6.4% | 144 out of 2860 genes, 5.0% | 0.016446 |
| 78 | secondary metabolic process | 25 out of 908 genes, 2.8% | 54 out of 2860 genes, 1.9% | 0.016883 |
| 79 | oligosaccharide metabolic process | 13 out of 908 genes, 1.4% | 24 out of 2860 genes, 0.8% | 0.018448 |
| 80 | ion homeostasis | 13 out of 908 genes, 1.4% | 24 out of 2860 genes, 0.8% | 0.018448 |
| 81 | response to stimulus | 254 out of 908 genes, 28.0% | 727 out of 2860 genes, 25.4% | 0.018546 |
| 82 | pentose metabolic process | 10 out of 908 genes, 1.1% | 17 out of 2860 genes, 0.6% | 0.018949 |
| 83 | chemical homeostasis | 15 out of 908 genes, 1.7% | 29 out of 2860 genes, 1.0% | 0.019452 |
| 84 | single-organism carbohydrate metabolic process | 80 out of 908 genes, 8.8% | 208 out of 2860 genes, 7.3% | 0.019733 |
| 85 | response to cadmium ion | 54 out of 908 genes, 5.9% | 134 out of 2860 genes, 4.7% | 0.020013 |
| 86 | response to inorganic substance | 75 out of 908 genes, 8.3% | 194 out of 2860 genes, 6.8% | 0.020753 |
| 87 | nucleosome assembly | 12 out of 908 genes, 1.3% | 22 out of 2860 genes, 0.8% | 0.021854 |
| 88 | phenylpropanoid biosynthetic process | 12 out of 908 genes, 1.3% | 22 out of 2860 genes, 0.8% | 0.021854 |
| 89 | nucleosome organization | 12 out of 908 genes, 1.3% | 22 out of 2860 genes, 0.8% | 0.021854 |
| 90 | protein-DNA complex assembly | 12 out of 908 genes, 1.3% | 22 out of 2860 genes, 0.8% | 0.021854 |
| 91 | protein-DNA complex subunit organization | 12 out of 908 genes, 1.3% | 22 out of 2860 genes, 0.8% | 0.021854 |
| 92 | pentose-phosphate shunt | 14 out of 908 genes, 1.5% | 27 out of 2860 genes, 0.9% | 0.023118 |
| 93 | NADPH regeneration | 14 out of 908 genes, 1.5% | 27 out of 2860 genes, 0.9% | 0.023118 |
| 94 | pyridine nucleotide metabolic process | 16 out of 908 genes, 1.8% | 32 out of 2860 genes, 1.1% | 0.023343 |
| 95 | nicotinamide nucleotide metabolic process | 16 out of 908 genes, 1.8% | 32 out of 2860 genes, 1.1% | 0.023343 |
| 96 | methionine metabolic process | 8 out of 908 genes, 0.9% | 13 out of 2860 genes, 0.5% | 0.025558 |
| 97 | response to hexose stimulus | 8 out of 908 genes, 0.9% | 13 out of 2860 genes, 0.5% | 0.025558 |
| 98 | response to monosaccharide stimulus | 8 out of 908 genes, 0.9% | 13 out of 2860 genes, 0.5% | 0.025558 |
| 99 | cellular ketone metabolic process | 8 out of 908 genes, 0.9% | 13 out of 2860 genes, 0.5% | 0.025558 |
| 100 | organic substance metabolic process | 530 out of 908 genes, 58.4% | 1593 out of 2860 genes, 55.7% | 0.027279 |
| 101 | sulfur amino acid biosynthetic process | 15 out of 908 genes, 1.7% | 30 out of 2860 genes, 1.0% | 0.02776 |
| 102 | cellular modified amino acid biosynthetic process | 7 out of 908 genes, 0.8% | 11 out of 2860 genes, 0.4% | 0.029364 |
| 103 | sulfur compound biosynthetic process | 23 out of 908 genes, 2.5% | 51 out of 2860 genes, 1.8% | 0.030162 |
| 104 | organic substance biosynthetic process | 248 out of 908 genes, 27.3% | 716 out of 2860 genes, 25.0% | 0.031099 |
| 105 | succinate metabolic process | 3 out of 908 genes, 0.3% | 3 out of 2860 genes, 0.1% | 0.031929 |
| 106 | asparaginyl-tRNA aminoacylation | 3 out of 908 genes, 0.3% | 3 out of 2860 genes, 0.1% | 0.031929 |
| 107 | S-adenosylmethionine biosynthetic process | 3 out of 908 genes, 0.3% | 3 out of 2860 genes, 0.1% | 0.031929 |
| 108 | pentose-phosphate shunt, non-oxidative branch | 3 out of 908 genes, 0.3% | 3 out of 2860 genes, 0.1% | 0.031929 |
| 109 | photosynthetic electron transport in photosystem II | 3 out of 908 genes, 0.3% | 3 out of 2860 genes, 0.1% | 0.031929 |
| 110 | regulation of protein catabolic process | 3 out of 908 genes, 0.3% | 3 out of 2860 genes, 0.1% | 0.031929 |
| 111 | cuticle development | 3 out of 908 genes, 0.3% | 3 out of 2860 genes, 0.1% | 0.031929 |
| 112 | anthocyanin-containing compound metabolic process | 3 out of 908 genes, 0.3% | 3 out of 2860 genes, 0.1% | 0.031929 |
| 113 | monosaccharide metabolic process | 49 out of 908 genes, 5.4% | 123 out of 2860 genes, 4.3% | 0.032315 |
| 114 | cysteine metabolic process | 12 out of 908 genes, 1.3% | 23 out of 2860 genes, 0.8% | 0.032791 |
| 115 | biosynthetic process | 267 out of 908 genes, 29.4% | 776 out of 2860 genes, 27.1% | 0.034878 |
| 116 | rRNA processing | 9 out of 908 genes, 1.0% | 16 out of 2860 genes, 0.6% | 0.036488 |
| 117 | rRNA metabolic process | 9 out of 908 genes, 1.0% | 16 out of 2860 genes, 0.6% | 0.036488 |
| 118 | glycine catabolic process | 5 out of 908 genes, 0.6% | 7 out of 2860 genes, 0.2% | 0.036589 |
| 119 | serine family amino acid catabolic process | 5 out of 908 genes, 0.6% | 7 out of 2860 genes, 0.2% | 0.036589 |
| 120 | response to glucose stimulus | 5 out of 908 genes, 0.6% | 7 out of 2860 genes, 0.2% | 0.036589 |
| 121 | systemic acquired resistance, salicylic acid mediated signaling pathway | 5 out of 908 genes, 0.6% | 7 out of 2860 genes, 0.2% | 0.036589 |
| 122 | hyperosmotic salinity response | 5 out of 908 genes, 0.6% | 7 out of 2860 genes, 0.2% | 0.036589 |
| 123 | pyridine-containing compound metabolic process | 17 out of 908 genes, 1.9% | 36 out of 2860 genes, 1.3% | 0.036711 |
| 124 | response to cold | 26 out of 908 genes, 2.9% | 60 out of 2860 genes, 2.1% | 0.037631 |
| 125 | organonitrogen compound biosynthetic process | 86 out of 908 genes, 9.5% | 231 out of 2860 genes, 8.1% | 0.037695 |
| 126 | DNA replication initiation | 4 out of 908 genes, 0.4% | 5 out of 2860 genes, 0.2% | 0.037764 |
| 127 | arginine biosynthetic process | 4 out of 908 genes, 0.4% | 5 out of 2860 genes, 0.2% | 0.037764 |
| 128 | secondary metabolite biosynthetic process | 16 out of 908 genes, 1.8% | 34 out of 2860 genes, 1.2% | 0.043554 |
| 129 | purine nucleoside biosynthetic process | 22 out of 908 genes, 2.4% | 50 out of 2860 genes, 1.7% | 0.044883 |
| 130 | purine ribonucleoside biosynthetic process | 22 out of 908 genes, 2.4% | 50 out of 2860 genes, 1.7% | 0.044883 |
| 131 | serine family amino acid biosynthetic process | 14 out of 908 genes, 1.5% | 29 out of 2860 genes, 1.0% | 0.045762 |
| 132 | DNA conformation change | 14 out of 908 genes, 1.5% | 29 out of 2860 genes, 1.0% | 0.045762 |
| 133 | water transport | 10 out of 908 genes, 1.1% | 19 out of 2860 genes, 0.7% | 0.046857 |
| 134 | response to wounding | 10 out of 908 genes, 1.1% | 19 out of 2860 genes, 0.7% | 0.046857 |
| 135 | fluid transport | 10 out of 908 genes, 1.1% | 19 out of 2860 genes, 0.7% | 0.046857 |
| 136 | systemic acquired resistance | 12 out of 908 genes, 1.3% | 24 out of 2860 genes, 0.8% | 0.047133 |
| 137 | chromatin assembly | 12 out of 908 genes, 1.3% | 24 out of 2860 genes, 0.8% | 0.047133 |
|  |  | **S3-VS-S2_ Cellular Component** |  |  |
|  | **Gene Ontology term** | **Cluster frequency** | **Protein frequency of use** | **P-value** |
| 1 | nucleosome | 14 out of 405 genes, 3.5% | 21 out of 2292 genes, 0.9% | 8.23E-07 |
| 2 | protein-DNA complex | 14 out of 405 genes, 3.5% | 21 out of 2292 genes, 0.9% | 8.23E-07 |
| 3 | chromatin | 15 out of 405 genes, 3.7% | 24 out of 2292 genes, 1.0% | 1.12E-06 |
| 4 | chromosomal part | 15 out of 405 genes, 3.7% | 24 out of 2292 genes, 1.0% | 1.12E-06 |
| 5 | intracellular part | 349 out of 405 genes, 86.2% | 1804 out of 2292 genes, 78.7% | 1.83E-05 |
| 6 | cytoplasm | 315 out of 405 genes, 77.8% | 1596 out of 2292 genes, 69.6% | 3.85E-05 |
| 7 | chromosome | 16 out of 405 genes, 4.0% | 33 out of 2292 genes, 1.4% | 4.21E-05 |
| 8 | vacuole | 50 out of 405 genes, 12.3% | 171 out of 2292 genes, 7.5% | 7.33E-05 |
| 9 | macromolecular complex | 109 out of 405 genes, 26.9% | 473 out of 2292 genes, 20.6% | 0.000491 |
| 10 | membrane coat | 10 out of 405 genes, 2.5% | 20 out of 2292 genes, 0.9% | 0.000912 |
| 11 | coated membrane | 10 out of 405 genes, 2.5% | 20 out of 2292 genes, 0.9% | 0.000912 |
| 12 | ribosomal subunit | 21 out of 405 genes, 5.2% | 63 out of 2292 genes, 2.7% | 0.001739 |
| 13 | large ribosomal subunit | 13 out of 405 genes, 3.2% | 33 out of 2292 genes, 1.4% | 0.002527 |
| 14 | clathrin coat | 5 out of 405 genes, 1.2% | 7 out of 2292 genes, 0.3% | 0.002589 |
| 15 | cytoplasmic part | 265 out of 405 genes, 65.4% | 1358 out of 2292 genes, 59.2% | 0.002959 |
| 16 | intracellular | 351 out of 405 genes, 86.7% | 1877 out of 2292 genes, 81.9% | 0.002996 |
| 17 | non-membrane-bounded organelle | 69 out of 405 genes, 17.0% | 293 out of 2292 genes, 12.8% | 0.003798 |
| 18 | intracellular non-membrane-bounded organelle | 69 out of 405 genes, 17.0% | 293 out of 2292 genes, 12.8% | 0.003798 |
| 19 | mitochondrial part | 22 out of 405 genes, 5.4% | 72 out of 2292 genes, 3.1% | 0.004675 |
| 20 | organelle membrane | 42 out of 405 genes, 10.4% | 164 out of 2292 genes, 7.2% | 0.005202 |
| 21 | mitochondrial proton-transporting ATP synthase complex, coupling factor F(o) | 3 out of 405 genes, 0.7% | 3 out of 2292 genes, 0.1% | 0.005484 |
| 22 | proton-transporting ATP synthase complex, coupling factor F(o) | 3 out of 405 genes, 0.7% | 3 out of 2292 genes, 0.1% | 0.005484 |
| 23 | anchored to membrane | 5 out of 405 genes, 1.2% | 8 out of 2292 genes, 0.3% | 0.005906 |
| 24 | cytosol | 64 out of 405 genes, 15.8% | 274 out of 2292 genes, 12.0% | 0.006557 |
| 25 | mitochondrial membrane | 19 out of 405 genes, 4.7% | 61 out of 2292 genes, 2.7% | 0.006656 |
| 26 | cytosolic large ribosomal subunit | 11 out of 405 genes, 2.7% | 29 out of 2292 genes, 1.3% | 0.007519 |
| 27 | mitochondrial envelope | 20 out of 405 genes, 4.9% | 66 out of 2292 genes, 2.9% | 0.007615 |
| 28 | organelle part | 127 out of 405 genes, 31.4% | 605 out of 2292 genes, 26.4% | 0.008049 |
| 29 | intracellular organelle part | 127 out of 405 genes, 31.4% | 605 out of 2292 genes, 26.4% | 0.008049 |
| 30 | ribonucleoprotein complex | 45 out of 405 genes, 11.1% | 183 out of 2292 genes, 8.0% | 0.008655 |
| 31 | AP-type membrane coat adaptor complex | 4 out of 405 genes, 1.0% | 6 out of 2292 genes, 0.3% | 0.01069 |
| 32 | clathrin adaptor complex | 4 out of 405 genes, 1.0% | 6 out of 2292 genes, 0.3% | 0.01069 |
| 33 | Golgi-associated vesicle membrane | 4 out of 405 genes, 1.0% | 6 out of 2292 genes, 0.3% | 0.01069 |
| 34 | proton-transporting two-sector ATPase complex, proton-transporting domain | 4 out of 405 genes, 1.0% | 6 out of 2292 genes, 0.3% | 0.01069 |
| 35 | mitochondrial inner membrane | 16 out of 405 genes, 4.0% | 51 out of 2292 genes, 2.2% | 0.011491 |
| 36 | proteasome complex | 11 out of 405 genes, 2.7% | 31 out of 2292 genes, 1.4% | 0.013152 |
| 37 | ribosome | 38 out of 405 genes, 9.4% | 155 out of 2292 genes, 6.8% | 0.016209 |
| 38 | mitochondrial outer membrane translocase complex | 3 out of 405 genes, 0.7% | 4 out of 2292 genes, 0.2% | 0.019045 |
| 39 | Golgi-associated vesicle | 4 out of 405 genes, 1.0% | 7 out of 2292 genes, 0.3% | 0.021492 |
| 40 | organelle inner membrane | 16 out of 405 genes, 4.0% | 55 out of 2292 genes, 2.4% | 0.024024 |
| 41 | nucleus | 82 out of 405 genes, 20.2% | 384 out of 2292 genes, 16.8% | 0.024262 |
| 42 | cytosolic ribosome | 22 out of 405 genes, 5.4% | 83 out of 2292 genes, 3.6% | 0.026655 |
| 43 | apoplast | 22 out of 405 genes, 5.4% | 83 out of 2292 genes, 3.6% | 0.026655 |
| 44 | vacuolar membrane | 16 out of 405 genes, 4.0% | 56 out of 2292 genes, 2.4% | 0.028356 |
| 45 | vacuolar part | 16 out of 405 genes, 4.0% | 56 out of 2292 genes, 2.4% | 0.028356 |
| 46 | cell | 376 out of 405 genes, 92.8% | 2068 out of 2292 genes, 90.2% | 0.028401 |
| 47 | cell part | 376 out of 405 genes, 92.8% | 2068 out of 2292 genes, 90.2% | 0.028401 |
| 48 | coated pit | 2 out of 405 genes, 0.5% | 2 out of 2292 genes, 0.1% | 0.03116 |
| 49 | clathrin coat of coated pit | 2 out of 405 genes, 0.5% | 2 out of 2292 genes, 0.1% | 0.03116 |
| 50 | membrane-bounded organelle | 263 out of 405 genes, 64.9% | 1393 out of 2292 genes, 60.8% | 0.032787 |
| 51 | intracellular membrane-bounded organelle | 263 out of 405 genes, 64.9% | 1393 out of 2292 genes, 60.8% | 0.032787 |
| 52 | proteasome core complex, alpha-subunit complex | 4 out of 405 genes, 1.0% | 8 out of 2292 genes, 0.3% | 0.037078 |
| 53 | COPI vesicle coat | 3 out of 405 genes, 0.7% | 5 out of 2292 genes, 0.2% | 0.041403 |
| 54 | COPI-coated vesicle | 3 out of 405 genes, 0.7% | 5 out of 2292 genes, 0.2% | 0.041403 |
| 55 | COPI-coated vesicle membrane | 3 out of 405 genes, 0.7% | 5 out of 2292 genes, 0.2% | 0.041403 |
| 56 | organelle | 280 out of 405 genes, 69.1% | 1499 out of 2292 genes, 65.4% | 0.045389 |
| 57 | intracellular organelle | 280 out of 405 genes, 69.1% | 1499 out of 2292 genes, 65.4% | 0.045389 |
| 58 | proteasome core complex | 6 out of 405 genes, 1.5% | 16 out of 2292 genes, 0.7% | 0.048121 |
|  |  | **S3-VS-S2_ Molecular Function** |  |  |
|  | **Gene Ontology term** | **Cluster frequency** | **Protein frequency of use** | **P-value** |
| 1 | oxidoreductase activity, acting on a sulfur group of donors | 17 out of 560 genes, 3.0% | 40 out of 3345 genes, 1.2% | 9.99E-05 |
| 2 | disulfide oxidoreductase activity | 14 out of 560 genes, 2.5% | 30 out of 3345 genes, 0.9% | 0.000123 |
| 3 | protein disulfide oxidoreductase activity | 12 out of 560 genes, 2.1% | 26 out of 3345 genes, 0.8% | 0.000429 |
| 4 | superoxide dismutase activity | 4 out of 560 genes, 0.7% | 4 out of 3345 genes, 0.1% | 0.000779 |
| 5 | oxidoreductase activity, acting on superoxide radicals as acceptor | 4 out of 560 genes, 0.7% | 4 out of 3345 genes, 0.1% | 0.000779 |
| 6 | antioxidant activity | 20 out of 560 genes, 3.6% | 59 out of 3345 genes, 1.8% | 0.000924 |
| 7 | nucleobase-containing compound kinase activity | 6 out of 560 genes, 1.1% | 9 out of 3345 genes, 0.3% | 0.001143 |
| 8 | phosphotransferase activity, phosphate group as acceptor | 5 out of 560 genes, 0.9% | 7 out of 3345 genes, 0.2% | 0.002021 |
| 9 | electron carrier activity | 20 out of 560 genes, 3.6% | 63 out of 3345 genes, 1.9% | 0.002298 |
| 10 | ammonia-lyase activity | 6 out of 560 genes, 1.1% | 10 out of 3345 genes, 0.3% | 0.002456 |
| 11 | phenylalanine ammonia-lyase activity | 5 out of 560 genes, 0.9% | 8 out of 3345 genes, 0.2% | 0.004649 |
| 12 | 3-hydroxyacyl-CoA dehydrogenase activity | 3 out of 560 genes, 0.5% | 3 out of 3345 genes, 0.1% | 0.004671 |
| 13 | hydrolase activity, acting on glycosyl bonds | 39 out of 560 genes, 7.0% | 159 out of 3345 genes, 4.8% | 0.006496 |
| 14 | structural constituent of ribosome | 30 out of 560 genes, 5.4% | 118 out of 3345 genes, 3.5% | 0.009573 |
| 15 | carbon-nitrogen lyase activity | 7 out of 560 genes, 1.2% | 16 out of 3345 genes, 0.5% | 0.010143 |
| 16 | carboxylic acid binding | 7 out of 560 genes, 1.2% | 16 out of 3345 genes, 0.5% | 0.010143 |
| 17 | tetrapyrrole binding | 18 out of 560 genes, 3.2% | 62 out of 3345 genes, 1.9% | 0.010493 |
| 18 | copper ion binding | 17 out of 560 genes, 3.0% | 58 out of 3345 genes, 1.7% | 0.011506 |
| 19 | structural molecule activity | 36 out of 560 genes, 6.4% | 150 out of 3345 genes, 4.5% | 0.012477 |
| 20 | amino acid binding | 6 out of 560 genes, 1.1% | 13 out of 3345 genes, 0.4% | 0.012773 |
| 21 | ligase activity | 27 out of 560 genes, 4.8% | 106 out of 3345 genes, 3.2% | 0.013263 |
| 22 | heme binding | 17 out of 560 genes, 3.0% | 59 out of 3345 genes, 1.8% | 0.013731 |
| 23 | lipid binding | 7 out of 560 genes, 1.2% | 17 out of 3345 genes, 0.5% | 0.014806 |
| 24 | nucleoside diphosphate kinase activity | 3 out of 560 genes, 0.5% | 4 out of 3345 genes, 0.1% | 0.016349 |
| 25 | ribosome binding | 3 out of 560 genes, 0.5% | 4 out of 3345 genes, 0.1% | 0.016349 |
| 26 | protein heterodimerization activity | 3 out of 560 genes, 0.5% | 4 out of 3345 genes, 0.1% | 0.016349 |
| 27 | translation elongation factor activity | 7 out of 560 genes, 1.2% | 18 out of 3345 genes, 0.5% | 0.020815 |
| 28 | threonine-type endopeptidase activity | 6 out of 560 genes, 1.1% | 15 out of 3345 genes, 0.4% | 0.027656 |
| 29 | threonine-type peptidase activity | 6 out of 560 genes, 1.1% | 15 out of 3345 genes, 0.4% | 0.027656 |
| 30 | ACP phosphopantetheine attachment site binding involved in fatty acid biosynthetic process | 2 out of 560 genes, 0.4% | 2 out of 3345 genes, 0.1% | 0.027986 |
| 31 | 3'-5'-exoribonuclease activity | 2 out of 560 genes, 0.4% | 2 out of 3345 genes, 0.1% | 0.027986 |
| 32 | biotin carboxylase activity | 2 out of 560 genes, 0.4% | 2 out of 3345 genes, 0.1% | 0.027986 |
| 33 | catalase activity | 2 out of 560 genes, 0.4% | 2 out of 3345 genes, 0.1% | 0.027986 |
| 34 | isocitrate dehydrogenase (NAD+) activity | 2 out of 560 genes, 0.4% | 2 out of 3345 genes, 0.1% | 0.027986 |
| 35 | poly(A)-specific ribonuclease activity | 2 out of 560 genes, 0.4% | 2 out of 3345 genes, 0.1% | 0.027986 |
| 36 | phosphoglycerate dehydrogenase activity | 2 out of 560 genes, 0.4% | 2 out of 3345 genes, 0.1% | 0.027986 |
| 37 | glutamine-tRNA ligase activity | 2 out of 560 genes, 0.4% | 2 out of 3345 genes, 0.1% | 0.027986 |
| 38 | cysteine-type endopeptidase inhibitor activity | 2 out of 560 genes, 0.4% | 2 out of 3345 genes, 0.1% | 0.027986 |
| 39 | ACP phosphopantetheine attachment site binding | 2 out of 560 genes, 0.4% | 2 out of 3345 genes, 0.1% | 0.027986 |
| 40 | prosthetic group binding | 2 out of 560 genes, 0.4% | 2 out of 3345 genes, 0.1% | 0.027986 |
| 41 | ATPase regulator activity | 2 out of 560 genes, 0.4% | 2 out of 3345 genes, 0.1% | 0.027986 |
| 42 | carboxypeptidase activity | 7 out of 560 genes, 1.2% | 19 out of 3345 genes, 0.6% | 0.028331 |
| 43 | ribonuclease activity | 4 out of 560 genes, 0.7% | 8 out of 3345 genes, 0.2% | 0.030953 |
| 44 | peroxidase activity | 13 out of 560 genes, 2.3% | 46 out of 3345 genes, 1.4% | 0.034118 |
| 45 | oxidoreductase activity, acting on peroxide as acceptor | 13 out of 560 genes, 2.3% | 46 out of 3345 genes, 1.4% | 0.034118 |
| 46 | ribonucleoprotein complex binding | 3 out of 560 genes, 0.5% | 5 out of 3345 genes, 0.1% | 0.035812 |
| 47 | galactosidase activity | 5 out of 560 genes, 0.9% | 12 out of 3345 genes, 0.4% | 0.036713 |
| 48 | protein binding | 41 out of 560 genes, 7.3% | 189 out of 3345 genes, 5.7% | 0.040924 |
| 49 | oxidoreductase activity, acting on the CH-OH group of donors, NAD or NADP as acceptor | 23 out of 560 genes, 4.1% | 96 out of 3345 genes, 2.9% | 0.041693 |
| 50 | ligase activity, forming carbon-nitrogen bonds | 11 out of 560 genes, 2.0% | 39 out of 3345 genes, 1.2% | 0.049868 |
|  |  | **S3-VS-S2_ Biological Process** |  |  |
|  | **Gene Ontology term** | **Cluster frequency** | **Protein frequency of use** | **P-value** |
| 1 | nucleosome assembly | 16 out of 490 genes, 3.3% | 22 out of 2860 genes, 0.8% | 1.20E-08 |
| 2 | nucleosome organization | 16 out of 490 genes, 3.3% | 22 out of 2860 genes, 0.8% | 1.20E-08 |
| 3 | protein-DNA complex assembly | 16 out of 490 genes, 3.3% | 22 out of 2860 genes, 0.8% | 1.20E-08 |
| 4 | protein-DNA complex subunit organization | 16 out of 490 genes, 3.3% | 22 out of 2860 genes, 0.8% | 1.20E-08 |
| 5 | chromatin assembly or disassembly | 18 out of 490 genes, 3.7% | 29 out of 2860 genes, 1.0% | 6.56E-08 |
| 6 | chromatin assembly | 16 out of 490 genes, 3.3% | 24 out of 2860 genes, 0.8% | 8.44E-08 |
| 7 | DNA packaging | 16 out of 490 genes, 3.3% | 25 out of 2860 genes, 0.9% | 1.98E-07 |
| 8 | DNA conformation change | 16 out of 490 genes, 3.3% | 29 out of 2860 genes, 1.0% | 3.33E-06 |
| 9 | chromatin organization | 22 out of 490 genes, 4.5% | 51 out of 2860 genes, 1.8% | 1.01E-05 |
| 10 | small molecule metabolic process | 135 out of 490 genes, 27.6% | 583 out of 2860 genes, 20.4% | 1.68E-05 |
| 11 | cellular component assembly | 31 out of 490 genes, 6.3% | 87 out of 2860 genes, 3.0% | 1.92E-05 |
| 12 | macromolecular complex assembly | 26 out of 490 genes, 5.3% | 69 out of 2860 genes, 2.4% | 3.00E-05 |
| 13 | cellular modified amino acid metabolic process | 11 out of 490 genes, 2.2% | 19 out of 2860 genes, 0.7% | 6.77E-05 |
| 14 | organic acid metabolic process | 82 out of 490 genes, 16.7% | 330 out of 2860 genes, 11.5% | 9.73E-05 |
| 15 | macromolecular complex subunit organization | 26 out of 490 genes, 5.3% | 74 out of 2860 genes, 2.6% | 0.00012 |
| 16 | carboxylic acid metabolic process | 81 out of 490 genes, 16.5% | 327 out of 2860 genes, 11.4% | 0.000122 |
| 17 | chromosome organization | 22 out of 490 genes, 4.5% | 59 out of 2860 genes, 2.1% | 0.000148 |
| 18 | oxoacid metabolic process | 81 out of 490 genes, 16.5% | 329 out of 2860 genes, 11.5% | 0.000152 |
| 19 | cellular macromolecular complex assembly | 23 out of 490 genes, 4.7% | 64 out of 2860 genes, 2.2% | 0.000201 |
| 20 | single-organism metabolic process | 200 out of 490 genes, 40.8% | 981 out of 2860 genes, 34.3% | 0.000567 |
| 21 | response to inorganic substance | 51 out of 490 genes, 10.4% | 194 out of 2860 genes, 6.8% | 0.000582 |
| 22 | homeostatic process | 23 out of 490 genes, 4.7% | 69 out of 2860 genes, 2.4% | 0.000702 |
| 23 | nucleoside triphosphate biosynthetic process | 15 out of 490 genes, 3.1% | 38 out of 2860 genes, 1.3% | 0.00085 |
| 24 | purine nucleoside triphosphate biosynthetic process | 15 out of 490 genes, 3.1% | 38 out of 2860 genes, 1.3% | 0.00085 |
| 25 | ribonucleoside triphosphate biosynthetic process | 15 out of 490 genes, 3.1% | 38 out of 2860 genes, 1.3% | 0.00085 |
| 26 | purine ribonucleoside triphosphate biosynthetic process | 15 out of 490 genes, 3.1% | 38 out of 2860 genes, 1.3% | 0.00085 |
| 27 | cell redox homeostasis | 15 out of 490 genes, 3.1% | 38 out of 2860 genes, 1.3% | 0.00085 |
| 28 | positive regulation of cellular component organization | 4 out of 490 genes, 0.8% | 4 out of 2860 genes, 0.1% | 0.000853 |
| 29 | cellular homeostasis | 20 out of 490 genes, 4.1% | 58 out of 2860 genes, 2.0% | 0.00096 |
| 30 | purine nucleoside biosynthetic process | 18 out of 490 genes, 3.7% | 50 out of 2860 genes, 1.7% | 0.000965 |
| 31 | purine ribonucleoside biosynthetic process | 18 out of 490 genes, 3.7% | 50 out of 2860 genes, 1.7% | 0.000965 |
| 32 | cellular component biogenesis | 35 out of 490 genes, 7.1% | 123 out of 2860 genes, 4.3% | 0.000968 |
| 33 | cellular amino acid metabolic process | 54 out of 490 genes, 11.0% | 213 out of 2860 genes, 7.4% | 0.001034 |
| 34 | translation | 51 out of 490 genes, 10.4% | 200 out of 2860 genes, 7.0% | 0.001244 |
| 35 | response to metal ion | 39 out of 490 genes, 8.0% | 144 out of 2860 genes, 5.0% | 0.001431 |
| 36 | glycerol ether metabolic process | 8 out of 490 genes, 1.6% | 15 out of 2860 genes, 0.5% | 0.001468 |
| 37 | ether metabolic process | 8 out of 490 genes, 1.6% | 15 out of 2860 genes, 0.5% | 0.001468 |
| 38 | aromatic amino acid family catabolic process | 7 out of 490 genes, 1.4% | 12 out of 2860 genes, 0.4% | 0.001492 |
| 39 | reactive oxygen species metabolic process | 7 out of 490 genes, 1.4% | 12 out of 2860 genes, 0.4% | 0.001492 |
| 40 | nucleoside biosynthetic process | 18 out of 490 genes, 3.7% | 53 out of 2860 genes, 1.9% | 0.002082 |
| 41 | ribonucleoside biosynthetic process | 18 out of 490 genes, 3.7% | 53 out of 2860 genes, 1.9% | 0.002082 |
| 42 | nucleotide phosphorylation | 5 out of 490 genes, 1.0% | 7 out of 2860 genes, 0.2% | 0.002248 |
| 43 | nucleotide biosynthetic process | 24 out of 490 genes, 4.9% | 80 out of 2860 genes, 2.8% | 0.002813 |
| 44 | nucleoside phosphate biosynthetic process | 24 out of 490 genes, 4.9% | 80 out of 2860 genes, 2.8% | 0.002813 |
| 45 | purine ribonucleotide biosynthetic process | 17 out of 490 genes, 3.5% | 51 out of 2860 genes, 1.8% | 0.003443 |
| 46 | organic acid catabolic process | 17 out of 490 genes, 3.5% | 51 out of 2860 genes, 1.8% | 0.003443 |
| 47 | small molecule catabolic process | 17 out of 490 genes, 3.5% | 51 out of 2860 genes, 1.8% | 0.003443 |
| 48 | single-organism catabolic process | 17 out of 490 genes, 3.5% | 51 out of 2860 genes, 1.8% | 0.003443 |
| 49 | carboxylic acid catabolic process | 17 out of 490 genes, 3.5% | 51 out of 2860 genes, 1.8% | 0.003443 |
| 50 | alpha-amino acid metabolic process | 35 out of 490 genes, 7.1% | 132 out of 2860 genes, 4.6% | 0.003665 |
| 51 | superoxide metabolic process | 4 out of 490 genes, 0.8% | 5 out of 2860 genes, 0.2% | 0.003684 |
| 52 | glycosyl compound biosynthetic process | 20 out of 490 genes, 4.1% | 65 out of 2860 genes, 2.3% | 0.004502 |
| 53 | biosynthetic process | 157 out of 490 genes, 32.0% | 776 out of 2860 genes, 27.1% | 0.004681 |
| 54 | response to cadmium ion | 35 out of 490 genes, 7.1% | 134 out of 2860 genes, 4.7% | 0.004778 |
| 55 | cellular response to nitrogen starvation | 3 out of 490 genes, 0.6% | 3 out of 2860 genes, 0.1% | 0.005004 |
| 56 | regulation of protein catabolic process | 3 out of 490 genes, 0.6% | 3 out of 2860 genes, 0.1% | 0.005004 |
| 57 | organic acid biosynthetic process | 40 out of 490 genes, 8.2% | 160 out of 2860 genes, 5.6% | 0.006041 |
| 58 | carboxylic acid biosynthetic process | 40 out of 490 genes, 8.2% | 160 out of 2860 genes, 5.6% | 0.006041 |
| 59 | ribonucleotide biosynthetic process | 18 out of 490 genes, 3.7% | 58 out of 2860 genes, 2.0% | 0.006249 |
| 60 | ribose phosphate biosynthetic process | 18 out of 490 genes, 3.7% | 58 out of 2860 genes, 2.0% | 0.006249 |
| 61 | ATP biosynthetic process | 12 out of 490 genes, 2.4% | 34 out of 2860 genes, 1.2% | 0.008046 |
| 62 | primary metabolic process | 282 out of 490 genes, 57.6% | 1502 out of 2860 genes, 52.5% | 0.008077 |
| 63 | purine nucleotide biosynthetic process | 17 out of 490 genes, 3.5% | 55 out of 2860 genes, 1.9% | 0.008136 |
| 64 | regulation of biological quality | 30 out of 490 genes, 6.1% | 115 out of 2860 genes, 4.0% | 0.00889 |
| 65 | purine-containing compound biosynthetic process | 18 out of 490 genes, 3.7% | 60 out of 2860 genes, 2.1% | 0.009183 |
| 66 | glutamine metabolic process | 4 out of 490 genes, 0.8% | 6 out of 2860 genes, 0.2% | 0.009557 |
| 67 | pyrimidine nucleoside triphosphate metabolic process | 4 out of 490 genes, 0.8% | 6 out of 2860 genes, 0.2% | 0.009557 |
| 68 | L-phenylalanine catabolic process | 5 out of 490 genes, 1.0% | 9 out of 2860 genes, 0.3% | 0.009975 |
| 69 | cellular biosynthetic process | 138 out of 490 genes, 28.2% | 685 out of 2860 genes, 24.0% | 0.010311 |
| 70 | alpha-amino acid catabolic process | 11 out of 490 genes, 2.2% | 31 out of 2860 genes, 1.1% | 0.010542 |
| 71 | purine nucleoside metabolic process | 34 out of 490 genes, 6.9% | 136 out of 2860 genes, 4.8% | 0.011011 |
| 72 | purine ribonucleoside metabolic process | 34 out of 490 genes, 6.9% | 136 out of 2860 genes, 4.8% | 0.011011 |
| 73 | hemicellulose metabolic process | 7 out of 490 genes, 1.4% | 16 out of 2860 genes, 0.6% | 0.01149 |
| 74 | cell wall macromolecule metabolic process | 8 out of 490 genes, 1.6% | 20 out of 2860 genes, 0.7% | 0.012961 |
| 75 | cellular amino acid catabolic process | 11 out of 490 genes, 2.2% | 32 out of 2860 genes, 1.1% | 0.013654 |
| 76 | monocarboxylic acid biosynthetic process | 15 out of 490 genes, 3.1% | 49 out of 2860 genes, 1.7% | 0.013818 |
| 77 | organic substance metabolic process | 295 out of 490 genes, 60.2% | 1593 out of 2860 genes, 55.7% | 0.015355 |
| 78 | organic substance biosynthetic process | 142 out of 490 genes, 29.0% | 716 out of 2860 genes, 25.0% | 0.016341 |
| 79 | cell wall polysaccharide metabolic process | 7 out of 490 genes, 1.4% | 17 out of 2860 genes, 0.6% | 0.016712 |
| 80 | DNA metabolic process | 19 out of 490 genes, 3.9% | 68 out of 2860 genes, 2.4% | 0.016714 |
| 81 | ribonucleoside metabolic process | 34 out of 490 genes, 6.9% | 140 out of 2860 genes, 4.9% | 0.017146 |
| 82 | cell cycle phase | 5 out of 490 genes, 1.0% | 10 out of 2860 genes, 0.3% | 0.017179 |
| 83 | nucleoside diphosphate phosphorylation | 3 out of 490 genes, 0.6% | 4 out of 2860 genes, 0.1% | 0.017456 |
| 84 | GTP biosynthetic process | 3 out of 490 genes, 0.6% | 4 out of 2860 genes, 0.1% | 0.017456 |
| 85 | UTP biosynthetic process | 3 out of 490 genes, 0.6% | 4 out of 2860 genes, 0.1% | 0.017456 |
| 86 | CTP biosynthetic process | 3 out of 490 genes, 0.6% | 4 out of 2860 genes, 0.1% | 0.017456 |
| 87 | mitochondrial membrane organization | 3 out of 490 genes, 0.6% | 4 out of 2860 genes, 0.1% | 0.017456 |
| 88 | nucleoside diphosphate metabolic process | 3 out of 490 genes, 0.6% | 4 out of 2860 genes, 0.1% | 0.017456 |
| 89 | pyrimidine nucleoside triphosphate biosynthetic process | 3 out of 490 genes, 0.6% | 4 out of 2860 genes, 0.1% | 0.017456 |
| 90 | pyrimidine ribonucleoside triphosphate metabolic process | 3 out of 490 genes, 0.6% | 4 out of 2860 genes, 0.1% | 0.017456 |
| 91 | pyrimidine ribonucleoside triphosphate biosynthetic process | 3 out of 490 genes, 0.6% | 4 out of 2860 genes, 0.1% | 0.017456 |
| 92 | CTP metabolic process | 3 out of 490 genes, 0.6% | 4 out of 2860 genes, 0.1% | 0.017456 |
| 93 | UTP metabolic process | 3 out of 490 genes, 0.6% | 4 out of 2860 genes, 0.1% | 0.017456 |
| 94 | organonitrogen compound metabolic process | 97 out of 490 genes, 19.8% | 470 out of 2860 genes, 16.4% | 0.017534 |
| 95 | M phase | 4 out of 490 genes, 0.8% | 7 out of 2860 genes, 0.2% | 0.019302 |
| 96 | histone methylation | 4 out of 490 genes, 0.8% | 7 out of 2860 genes, 0.2% | 0.019302 |
| 97 | small molecule biosynthetic process | 46 out of 490 genes, 9.4% | 202 out of 2860 genes, 7.1% | 0.019909 |
| 98 | organelle organization | 40 out of 490 genes, 8.2% | 172 out of 2860 genes, 6.0% | 0.020872 |
| 99 | response to oxygen-containing compound | 33 out of 490 genes, 6.7% | 137 out of 2860 genes, 4.8% | 0.021047 |
| 100 | nucleobase-containing small molecule metabolic process | 48 out of 490 genes, 9.8% | 213 out of 2860 genes, 7.4% | 0.021185 |
| 101 | response to chemical stimulus | 70 out of 490 genes, 14.3% | 329 out of 2860 genes, 11.5% | 0.022448 |
| 102 | response to stress | 83 out of 490 genes, 16.9% | 399 out of 2860 genes, 14.0% | 0.023092 |
| 103 | carbohydrate derivative biosynthetic process | 25 out of 490 genes, 5.1% | 99 out of 2860 genes, 3.5% | 0.024144 |
| 104 | nitrogen compound metabolic process | 124 out of 490 genes, 25.3% | 625 out of 2860 genes, 21.9% | 0.025459 |
| 105 | nucleoside triphosphate metabolic process | 30 out of 490 genes, 6.1% | 124 out of 2860 genes, 4.3% | 0.025594 |
| 106 | purine-containing compound metabolic process | 35 out of 490 genes, 7.1% | 149 out of 2860 genes, 5.2% | 0.025717 |
| 107 | nucleoside phosphate metabolic process | 45 out of 490 genes, 9.2% | 200 out of 2860 genes, 7.0% | 0.025916 |
| 108 | nucleotide metabolic process | 45 out of 490 genes, 9.2% | 200 out of 2860 genes, 7.0% | 0.025916 |
| 109 | L-phenylalanine metabolic process | 5 out of 490 genes, 1.0% | 11 out of 2860 genes, 0.4% | 0.027142 |
| 110 | cellular modified amino acid biosynthetic process | 5 out of 490 genes, 1.0% | 11 out of 2860 genes, 0.4% | 0.027142 |
| 111 | regulation of translational termination | 2 out of 490 genes, 0.4% | 2 out of 2860 genes, 0.1% | 0.029304 |
| 112 | translational frameshifting | 2 out of 490 genes, 0.4% | 2 out of 2860 genes, 0.1% | 0.029304 |
| 113 | outer mitochondrial membrane organization | 2 out of 490 genes, 0.4% | 2 out of 2860 genes, 0.1% | 0.029304 |
| 114 | lipopolysaccharide metabolic process | 2 out of 490 genes, 0.4% | 2 out of 2860 genes, 0.1% | 0.029304 |
| 115 | aspartate family amino acid catabolic process | 2 out of 490 genes, 0.4% | 2 out of 2860 genes, 0.1% | 0.029304 |
| 116 | lipopolysaccharide biosynthetic process | 2 out of 490 genes, 0.4% | 2 out of 2860 genes, 0.1% | 0.029304 |
| 117 | response to herbicide | 2 out of 490 genes, 0.4% | 2 out of 2860 genes, 0.1% | 0.029304 |
| 118 | L-methionine biosynthetic process from S-adenosylmethionine | 2 out of 490 genes, 0.4% | 2 out of 2860 genes, 0.1% | 0.029304 |
| 119 | keto-3-deoxy-D-manno-octulosonic acid biosynthetic process | 2 out of 490 genes, 0.4% | 2 out of 2860 genes, 0.1% | 0.029304 |
| 120 | L-methionine salvage from methylthioadenosine | 2 out of 490 genes, 0.4% | 2 out of 2860 genes, 0.1% | 0.029304 |
| 121 | S-adenosylmethionine cycle | 2 out of 490 genes, 0.4% | 2 out of 2860 genes, 0.1% | 0.029304 |
| 122 | amino acid salvage | 2 out of 490 genes, 0.4% | 2 out of 2860 genes, 0.1% | 0.029304 |
| 123 | positive regulation of protein complex disassembly | 2 out of 490 genes, 0.4% | 2 out of 2860 genes, 0.1% | 0.029304 |
| 124 | protein import into mitochondrial outer membrane | 2 out of 490 genes, 0.4% | 2 out of 2860 genes, 0.1% | 0.029304 |
| 125 | positive regulation of translation | 2 out of 490 genes, 0.4% | 2 out of 2860 genes, 0.1% | 0.029304 |
| 126 | positive regulation of translational elongation | 2 out of 490 genes, 0.4% | 2 out of 2860 genes, 0.1% | 0.029304 |
| 127 | positive regulation of translational termination | 2 out of 490 genes, 0.4% | 2 out of 2860 genes, 0.1% | 0.029304 |
| 128 | keto-3-deoxy-D-manno-octulosonic acid metabolic process | 2 out of 490 genes, 0.4% | 2 out of 2860 genes, 0.1% | 0.029304 |
| 129 | anaphase | 2 out of 490 genes, 0.4% | 2 out of 2860 genes, 0.1% | 0.029304 |
| 130 | L-methionine salvage | 2 out of 490 genes, 0.4% | 2 out of 2860 genes, 0.1% | 0.029304 |
| 131 | cellular catabolic process | 51 out of 490 genes, 10.4% | 233 out of 2860 genes, 8.1% | 0.03003 |
| 132 | glycosyl compound metabolic process | 37 out of 490 genes, 7.6% | 161 out of 2860 genes, 5.6% | 0.030617 |
| 133 | single-organism biosynthetic process | 49 out of 490 genes, 10.0% | 223 out of 2860 genes, 7.8% | 0.031027 |
| 134 | pollination | 8 out of 490 genes, 1.6% | 23 out of 2860 genes, 0.8% | 0.031458 |
| 135 | multi-multicellular organism process | 8 out of 490 genes, 1.6% | 23 out of 2860 genes, 0.8% | 0.031458 |
| 136 | macromolecule methylation | 7 out of 490 genes, 1.4% | 19 out of 2860 genes, 0.7% | 0.031755 |
| 137 | nucleoside metabolic process | 34 out of 490 genes, 6.9% | 147 out of 2860 genes, 5.1% | 0.034227 |
| 138 | purine nucleoside triphosphate metabolic process | 29 out of 490 genes, 5.9% | 122 out of 2860 genes, 4.3% | 0.034775 |
| 139 | ribonucleoside triphosphate metabolic process | 29 out of 490 genes, 5.9% | 122 out of 2860 genes, 4.3% | 0.034775 |
| 140 | purine ribonucleoside triphosphate metabolic process | 29 out of 490 genes, 5.9% | 122 out of 2860 genes, 4.3% | 0.034775 |
| 141 | jasmonic acid biosynthetic process | 3 out of 490 genes, 0.6% | 5 out of 2860 genes, 0.2% | 0.038113 |
| 142 | cellular response to nitrogen levels | 3 out of 490 genes, 0.6% | 5 out of 2860 genes, 0.2% | 0.038113 |
| 143 | S-adenosylmethionine metabolic process | 3 out of 490 genes, 0.6% | 5 out of 2860 genes, 0.2% | 0.038113 |
| 144 | organelle assembly | 3 out of 490 genes, 0.6% | 5 out of 2860 genes, 0.2% | 0.038113 |
| 145 | cell proliferation | 5 out of 490 genes, 1.0% | 12 out of 2860 genes, 0.4% | 0.040132 |
| 146 | xylan metabolic process | 5 out of 490 genes, 1.0% | 12 out of 2860 genes, 0.4% | 0.040132 |
| 147 | multi-organism reproductive process | 8 out of 490 genes, 1.6% | 24 out of 2860 genes, 0.8% | 0.040355 |
| 148 | response to oxidative stress | 22 out of 490 genes, 4.5% | 89 out of 2860 genes, 3.1% | 0.04143 |
| 149 | nucleobase-containing compound metabolic process | 79 out of 490 genes, 16.1% | 388 out of 2860 genes, 13.6% | 0.042601 |
| 150 | monocarboxylic acid metabolic process | 25 out of 490 genes, 5.1% | 105 out of 2860 genes, 3.7% | 0.046997 |
| 151 | purine nucleotide metabolic process | 32 out of 490 genes, 6.5% | 141 out of 2860 genes, 4.9% | 0.049695 |
