# Supplementary material for: An Integrated Transcriptome and Proteome Analysis Reveals Putative Regulators of Adventitious Root Formation in Taxodium ‘Zhongshanshan’
Source: Int J Mol Sci. 2019 Mar 11;20(5):1225. doi: 10.3390/ijms20051225 (PMC6429173; doi:10.3390/ijms20051225)
Supplement: Supplementary file 1 [file ijms-20-01225-s001.zip › Supplementary material20190227/Table S8.docx]

**Table S8** The result of KEGG pathway classification and functional enrichment of DEPs

|  |  | S1-VS-S0 |  |  |  |
| --- | --- | --- | --- | --- | --- |
|  | **Pathway** | **Diff Proteins with pathway annotation (917)** | **All Proteins with pathway annotation (6017)** | **Pvalue** | **Pathway ID** |
| 1 | Metabolic pathways | 376 (41%) | 1780 (29.58%) | 6.40E-16 | ko01100 |
| 2 | Biosynthesis of secondary metabolites | 245 (26.72%) | 1131 (18.8%) | 8.42E-11 | ko01110 |
| 3 | Photosynthesis | 19 (2.07%) | 32 (0.53%) | 1.21E-08 | ko00195 |
| 4 | Photosynthesis - antenna proteins | 9 (0.98%) | 13 (0.22%) | 1.71E-05 | ko00196 |
| 5 | Carbon metabolism | 60 (6.54%) | 237 (3.94%) | 2.64E-05 | ko01200 |
| 6 | Carbon fixation in photosynthetic organisms | 25 (2.73%) | 72 (1.2%) | 3.12E-05 | ko00710 |
| 7 | Glyoxylate and dicarboxylate metabolism | 22 (2.4%) | 63 (1.05%) | 8.39E-05 | ko00630 |
| 8 | Phenylpropanoid biosynthesis | 52 (5.67%) | 211 (3.51%) | 0.00019016 | ko00940 |
| 9 | Glycolysis / Gluconeogenesis | 33 (3.6%) | 120 (1.99%) | 0.00035449 | ko00010 |
| 10 | Biosynthesis of amino acids | 50 (5.45%) | 206 (3.42%) | 0.0003715 | ko01230 |
| 11 | Amino sugar and nucleotide sugar metabolism | 39 (4.25%) | 151 (2.51%) | 0.00043913 | ko00520 |
| 12 | Glycine, serine and threonine metabolism | 26 (2.84%) | 90 (1.5%) | 0.00065092 | ko00260 |
| 13 | Ascorbate and aldarate metabolism | 20 (2.18%) | 67 (1.11%) | 0.0017152 | ko00053 |
| 14 | Ribosome | 51 (5.56%) | 227 (3.77%) | 0.00210531 | ko03010 |
| 15 | Flavonoid biosynthesis | 22 (2.4%) | 82 (1.36%) | 0.00454193 | ko00941 |
| 16 | alpha-Linolenic acid metabolism | 16 (1.74%) | 55 (0.91%) | 0.00634159 | ko00592 |
| 17 | Peroxisome | 27 (2.94%) | 110 (1.83%) | 0.00673831 | ko04146 |
| 18 | Phagosome | 16 (1.74%) | 58 (0.96%) | 0.01093536 | ko04145 |
| 19 | Alanine, aspartate and glutamate metabolism | 12 (1.31%) | 43 (0.71%) | 0.02355391 | ko00250 |
| 20 | Tyrosine metabolism | 15 (1.64%) | 58 (0.96%) | 0.02421332 | ko00350 |
| 21 | Citrate cycle (TCA cycle) | 14 (1.53%) | 54 (0.9%) | 0.02830621 | ko00020 |
| 22 | Glutathione metabolism | 17 (1.85%) | 70 (1.16%) | 0.03086953 | ko00480 |
| 23 | Fatty acid degradation | 12 (1.31%) | 45 (0.75%) | 0.03312927 | ko00071 |
| 24 | Cysteine and methionine metabolism | 17 (1.85%) | 71 (1.18%) | 0.03504337 | ko00270 |
|  |  | **S2-VS-S1** |  |  |  |
|  | **Pathway** | **Diff Proteins with pathway annotation (1615)** | **All Proteins with pathway annotation (6017)** | **Pvalue** | **Pathway ID** |
| 1 | Metabolic pathways | 641 (39.69%) | 1780 (29.58%) | 9.20E-25 | ko01100 |
| 2 | Biosynthesis of secondary metabolites | 405 (25.08%) | 1131 (18.8%) | 9.79E-14 | ko01110 |
| 3 | Phenylpropanoid biosynthesis | 96 (5.94%) | 211 (3.51%) | 2.45E-09 | ko00940 |
| 4 | Biosynthesis of amino acids | 92 (5.7%) | 206 (3.42%) | 1.66E-08 | ko01230 |
| 5 | Photosynthesis | 24 (1.49%) | 32 (0.53%) | 1.73E-08 | ko00195 |
| 6 | Carbon metabolism | 100 (6.19%) | 237 (3.94%) | 1.33E-07 | ko01200 |
| 7 | Amino sugar and nucleotide sugar metabolism | 66 (4.09%) | 151 (2.51%) | 4.52E-06 | ko00520 |
| 8 | Cysteine and methionine metabolism | 36 (2.23%) | 71 (1.18%) | 1.44E-05 | ko00270 |
| 9 | Carbon fixation in photosynthetic organisms | 36 (2.23%) | 72 (1.2%) | 2.14E-05 | ko00710 |
| 10 | Photosynthesis - antenna proteins | 11 (0.68%) | 13 (0.22%) | 2.26E-05 | ko00196 |
| 11 | Proteasome | 26 (1.61%) | 48 (0.8%) | 5.22E-05 | ko03050 |
| 12 | Nitrogen metabolism | 17 (1.05%) | 27 (0.45%) | 8.65E-05 | ko00910 |
| 13 | Fructose and mannose metabolism | 34 (2.11%) | 71 (1.18%) | 0.00011003 | ko00051 |
| 14 | Starch and sucrose metabolism | 73 (4.52%) | 189 (3.14%) | 0.00022087 | ko00500 |
| 15 | Ribosome | 82 (5.08%) | 227 (3.77%) | 0.00110494 | ko03010 |
| 16 | Pentose phosphate pathway | 22 (1.36%) | 45 (0.75%) | 0.00124935 | ko00030 |
| 17 | Glycosphingolipid biosynthesis - globo series | 12 (0.74%) | 20 (0.33%) | 0.00180973 | ko00603 |
| 18 | Glyoxylate and dicarboxylate metabolism | 28 (1.73%) | 63 (1.05%) | 0.00187058 | ko00630 |
| 19 | Glycolysis / Gluconeogenesis | 47 (2.91%) | 120 (1.99%) | 0.00199974 | ko00010 |
| 20 | Pyruvate metabolism | 38 (2.35%) | 95 (1.58%) | 0.00338663 | ko00620 |
| 21 | Other glycan degradation | 24 (1.49%) | 54 (0.9%) | 0.00385629 | ko00511 |
| 22 | Citrate cycle (TCA cycle) | 24 (1.49%) | 54 (0.9%) | 0.00385629 | ko00020 |
| 23 | 2-Oxocarboxylic acid metabolism | 25 (1.55%) | 57 (0.95%) | 0.00398989 | ko01210 |
| 24 | Arginine biosynthesis | 16 (0.99%) | 32 (0.53%) | 0.00427372 | ko00220 |
| 25 | Alanine, aspartate and glutamate metabolism | 20 (1.24%) | 43 (0.71%) | 0.00429522 | ko00250 |
| 26 | Ascorbate and aldarate metabolism | 28 (1.73%) | 67 (1.11%) | 0.0054775 | ko00053 |
| 27 | Glycine, serine and threonine metabolism | 35 (2.17%) | 90 (1.5%) | 0.00806978 | ko00260 |
| 28 | Oxidative phosphorylation | 31 (1.92%) | 78 (1.3%) | 0.0085757 | ko00190 |
| 29 | Cyanoamino acid metabolism | 28 (1.73%) | 69 (1.15%) | 0.00880046 | ko00460 |
| 30 | Glycerolipid metabolism | 39 (2.41%) | 103 (1.71%) | 0.00894504 | ko00561 |
| 31 | Flavonoid biosynthesis | 32 (1.98%) | 82 (1.36%) | 0.01039514 | ko00941 |
| 32 | Propanoate metabolism | 17 (1.05%) | 39 (0.65%) | 0.01741832 | ko00640 |
| 33 | Galactose metabolism | 28 (1.73%) | 73 (1.21%) | 0.02035646 | ko00052 |
| 34 | Tyrosine metabolism | 23 (1.42%) | 58 (0.96%) | 0.02238774 | ko00350 |
| 35 | Arginine and proline metabolism | 18 (1.11%) | 43 (0.71%) | 0.02308035 | ko00330 |
| 36 | Peroxisome | 39 (2.41%) | 110 (1.83%) | 0.02799376 | ko04146 |
| 37 | Pentose and glucuronate interconversions | 32 (1.98%) | 88 (1.46%) | 0.03073862 | ko00040 |
| 38 | Phenylalanine metabolism | 20 (1.24%) | 51 (0.85%) | 0.03601935 | ko00360 |
| 39 | Glycosaminoglycan degradation | 9 (0.56%) | 19 (0.32%) | 0.04404451 | ko00531 |
| 40 | Valine, leucine and isoleucine degradation | 18 (1.11%) | 46 (0.76%) | 0.04625563 | ko00280 |
|  |  | **S3-VS-S2** |  |  |  |
|  | **Pathway** | **Diff Proteins with pathway annotation (889)** | **All Proteins with pathway annotation (6017)** | **Pvalue** | **Pathway ID** |
| 1 | Phenylpropanoid biosynthesis | 65 (7.31%) | 211 (3.51%) | 1.29E-09 | ko00940 |
| 2 | Metabolic pathways | 317 (35.66%) | 1780 (29.58%) | 1.33E-05 | ko01100 |
| 3 | Ribosome | 54 (6.07%) | 227 (3.77%) | 0.00016796 | ko03010 |
| 4 | Oxidative phosphorylation | 24 (2.7%) | 78 (1.3%) | 0.00023398 | ko00190 |
| 5 | Biosynthesis of secondary metabolites | 203 (22.83%) | 1131 (18.8%) | 0.00061647 | ko01110 |
| 6 | Fatty acid metabolism | 19 (2.14%) | 64 (1.06%) | 0.00164395 | ko01212 |
| 7 | Proteasome | 15 (1.69%) | 48 (0.8%) | 0.00285409 | ko03050 |
| 8 | Other glycan degradation | 15 (1.69%) | 54 (0.9%) | 0.00958837 | ko00511 |
| 9 | Fatty acid degradation | 13 (1.46%) | 45 (0.75%) | 0.01094523 | ko00071 |
| 10 | Amino sugar and nucleotide sugar metabolism | 32 (3.6%) | 151 (2.51%) | 0.01978589 | ko00520 |
| 11 | Fatty acid elongation | 6 (0.67%) | 16 (0.27%) | 0.02180755 | ko00062 |
| 12 | alpha-Linolenic acid metabolism | 14 (1.57%) | 55 (0.91%) | 0.02584456 | ko00592 |
| 13 | Phenylalanine metabolism | 13 (1.46%) | 51 (0.85%) | 0.03076873 | ko00360 |
| 14 | Degradation of aromatic compounds | 5 (0.56%) | 13 (0.22%) | 0.03204618 | ko01220 |
| 15 | Glycosaminoglycan degradation | 6 (0.67%) | 19 (0.32%) | 0.05011916 | ko00531 |
| 16 | Cysteine and methionine metabolism | 16 (1.8%) | 71 (1.18%) | 0.05158017 | ko00270 |
| 17 | Fatty acid biosynthesis | 10 (1.12%) | 40 (0.66%) | 0.061142 | ko00061 |
| 18 | Glycosphingolipid biosynthesis - globo series | 6 (0.67%) | 20 (0.33%) | 0.06297971 | ko00603 |
| 19 | Galactose metabolism | 16 (1.8%) | 73 (1.21%) | 0.06412274 | ko00052 |
| 20 | Glycerolipid metabolism | 21 (2.36%) | 103 (1.71%) | 0.07372903 | ko00561 |
| 21 | Glutathione metabolism | 15 (1.69%) | 70 (1.16%) | 0.08397333 | ko00480 |
| 22 | Glycosphingolipid biosynthesis - ganglio series | 4 (0.45%) | 13 (0.22%) | 0.1127982 | ko00604 |
| 23 | Folate biosynthesis | 4 (0.45%) | 13 (0.22%) | 0.1127982 | ko00790 |
| 24 | 2-Oxocarboxylic acid metabolism | 12 (1.35%) | 57 (0.95%) | 0.1260851 | ko01210 |
| 25 | Flavonoid biosynthesis | 16 (1.8%) | 82 (1.36%) | 0.1448992 | ko00941 |
| 26 | Biosynthesis of amino acids | 36 (4.05%) | 206 (3.42%) | 0.1557717 | ko01230 |
| 27 | Ascorbate and aldarate metabolism | 13 (1.46%) | 67 (1.11%) | 0.1815751 | ko00053 |
| 28 | Peroxisome | 20 (2.25%) | 110 (1.83%) | 0.1871811 | ko04146 |
| 29 | Linoleic acid metabolism | 7 (0.79%) | 34 (0.57%) | 0.2285102 | ko00591 |
| 30 | Porphyrin and chlorophyll metabolism | 11 (1.24%) | 58 (0.96%) | 0.2299759 | ko00860 |
| 31 | Sphingolipid metabolism | 8 (0.9%) | 40 (0.66%) | 0.2305645 | ko00600 |
| 32 | Selenocompound metabolism | 4 (0.45%) | 17 (0.28%) | 0.2354816 | ko00450 |
| 33 | Protein processing in endoplasmic reticulum | 29 (3.26%) | 172 (2.86%) | 0.2462745 | ko04141 |
| 34 | Photosynthesis - antenna proteins | 3 (0.34%) | 13 (0.22%) | 0.299421 | ko00196 |
| 35 | Nicotinate and nicotinamide metabolism | 4 (0.45%) | 19 (0.32%) | 0.3054566 | ko00760 |
| 36 | Aminoacyl-tRNA biosynthesis | 13 (1.46%) | 76 (1.26%) | 0.3284893 | ko00970 |
| 37 | Arginine biosynthesis | 6 (0.67%) | 32 (0.53%) | 0.3318749 | ko00220 |
| 38 | SNARE interactions in vesicular transport | 5 (0.56%) | 26 (0.43%) | 0.3370773 | ko04130 |
| 39 | Nitrogen metabolism | 5 (0.56%) | 27 (0.45%) | 0.3683837 | ko00910 |
| 40 | Ubiquinone and other terpenoid-quinone biosynthesis | 5 (0.56%) | 27 (0.45%) | 0.3683837 | ko00130 |
| 41 | Biosynthesis of unsaturated fatty acids | 5 (0.56%) | 28 (0.47%) | 0.399711 | ko01040 |
| 42 | Citrate cycle (TCA cycle) | 9 (1.01%) | 54 (0.9%) | 0.4037712 | ko00020 |
| 43 | Biotin metabolism | 4 (0.45%) | 22 (0.37%) | 0.4129208 | ko00780 |
| 44 | Stilbenoid, diarylheptanoid and gingerol biosynthesis | 6 (0.67%) | 35 (0.58%) | 0.4161913 | ko00945 |
| 45 | Valine, leucine and isoleucine biosynthesis | 3 (0.34%) | 16 (0.27%) | 0.4283894 | ko00290 |
| 46 | Alanine, aspartate and glutamate metabolism | 7 (0.79%) | 43 (0.71%) | 0.4547084 | ko00250 |
| 47 | Carbon metabolism | 36 (4.05%) | 237 (3.94%) | 0.4560503 | ko01200 |
| 48 | Glyoxylate and dicarboxylate metabolism | 10 (1.12%) | 63 (1.05%) | 0.4563275 | ko00630 |
| 49 | Plant-pathogen interaction | 28 (3.15%) | 186 (3.09%) | 0.4891528 | ko04626 |
| 50 | Phagosome | 9 (1.01%) | 58 (0.96%) | 0.4929198 | ko04145 |
| 51 | Tyrosine metabolism | 9 (1.01%) | 58 (0.96%) | 0.4929198 | ko00350 |
