# Supplementary material for: An Integrated Transcriptome and Proteome Analysis Reveals Putative Regulators of Adventitious Root Formation in Taxodium ‘Zhongshanshan’
Source: Int J Mol Sci. 2019 Mar 11;20(5):1225. doi: 10.3390/ijms20051225 (PMC6429173; doi:10.3390/ijms20051225)
Supplement: Supplementary file 1 [file ijms-20-01225-s001.zip › Supplementary material20190227/Table S9.docx]

**Table S9** The result of KEGG pathway classification and functional enrichment of S0-VS-S1_3 and S0-VS-S1_7

|  |  |  | S0-VS-S1_3 Pathway Enrichment |  |  |  |
| --- | --- | --- | --- | --- | --- | --- |
|  | **Pathway** | **DEGs genes with pathway annotation (146)** | **All genes with pathway annotation (20073)** | **Pvalue** | **Qvalue** | **Pathway ID** |
| 1 | Indole alkaloid biosynthesis | 7 (4.79%) | 45 (0.22%) | 0 | 0.000002 | ko00901 |
| 2 | Phenylpropanoid biosynthesis | 19 (13.01%) | 819 (4.08%) | 0.000008 | 0.000249 | ko00940 |
| 3 | Flavonoid biosynthesis | 12 (8.22%) | 352 (1.75%) | 0.000011 | 0.000249 | ko00941 |
| 4 | Cutin, suberine and wax biosynthesis | 6 (4.11%) | 118 (0.59%) | 0.000225 | 0.004003 | ko00073 |
| 5 | Glutathione metabolism | 7 (4.79%) | 271 (1.35%) | 0.003737 | 0.05306 | ko00480 |
| 6 | Ascorbate and aldarate metabolism | 6 (4.11%) | 247 (1.23%) | 0.009491 | 0.112316 | ko00053 |
| 7 | Glucosinolate biosynthesis | 2 (1.37%) | 27 (0.13%) | 0.016373 | 0.166068 | ko00966 |
| 8 | Arginine and proline metabolism | 5 (3.42%) | 232 (1.16%) | 0.027468 | 0.243774 | ko00330 |
| 9 | Histidine metabolism | 2 (1.37%) | 47 (0.23%) | 0.045902 | 0.328975 | ko00340 |
| 10 | Carotenoid biosynthesis | 4 (2.74%) | 185 (0.92%) | 0.046335 | 0.328975 | ko00906 |
|  |  |  | **S0-VS-S1_7 Pathway Enrichment** |  |  |  |
|  | **Pathway** | **DEGs genes with pathway annotation (110)** | **All genes with pathway annotation (20073)** | **Pvalue** | **Qvalue** | **Pathway ID** |
| 1 | Photosynthesis - antenna proteins | 5 (4.55%) | 33 (0.16%) | 0.000001 | 0.000071 | ko00196 |
| 2 | Photosynthesis | 5 (4.55%) | 98 (0.49%) | 0.000204 | 0.007661 | ko00195 |
| 3 | Carbon fixation in photosynthetic organisms | 7 (6.36%) | 267 (1.33%) | 0.000676 | 0.0169 | ko00710 |
| 4 | Carbon metabolism | 12 (10.91%) | 875 (4.36%) | 0.003049 | 0.057171 | ko01200 |
| 5 | Glyoxylate and dicarboxylate metabolism | 5 (4.55%) | 279 (1.39%) | 0.018797 | 0.28195 | ko00630 |
| 6 | Porphyrin and chlorophyll metabolism | 3 (2.73%) | 119 (0.59%) | 0.027725 | 0.299754 | ko00860 |
| 7 | Base excision repair | 3 (2.73%) | 126 (0.63%) | 0.032073 | 0.299754 | ko03410 |
| 8 | Cysteine and methionine metabolism | 5 (4.55%) | 325 (1.62%) | 0.033351 | 0.299754 | ko00270 |
| 9 | Phenylpropanoid biosynthesis | 9 (8.18%) | 819 (4.08%) | 0.03597 | 0.299754 | ko00940 |
| 10 | alpha-Linolenic acid metabolism | 4 (3.64%) | 237 (1.18%) | 0.041503 | 0.303178 | ko00592 |
| 11 | Flavonoid biosynthesis | 5 (4.55%) | 352 (1.75%) | 0.044466 | 0.303178 | ko00941 |
