# Supplementary material for: An Integrated Transcriptome and Proteome Analysis Reveals Putative Regulators of Adventitious Root Formation in Taxodium ‘Zhongshanshan’
Source: Int J Mol Sci. 2019 Mar 11;20(5):1225. doi: 10.3390/ijms20051225 (PMC6429173; doi:10.3390/ijms20051225)
Supplement: Supplementary file 1 [file ijms-20-01225-s001.zip › Supplementary material20190227/~WRL2415.tmp]

**Table S6** Quantification repeat analysis of proteins

| Compare_group | mean CV |
| --- | --- |
| S1 vs. S0 | 0.13 |
| S2 vs. S1 | 0.16 |
| S3 vs. S2 | 0.13 |
