# Supplementary material for: An Integrated Transcriptome and Proteome Analysis Reveals Putative Regulators of Adventitious Root Formation in Taxodium ‘Zhongshanshan’
Source: Int J Mol Sci. 2019 Mar 11;20(5):1225. doi: 10.3390/ijms20051225 (PMC6429173; doi:10.3390/ijms20051225)
Supplement: Supplementary file 1 [file ijms-20-01225-s001.zip › Supplementary material20190227/~WRL4002.tmp]

**Table S2** Quality metrics of Unigenes

| Sample | Total Number | Total Length | Mean Length | N50 | N70 | N90 | GC(%) |
| --- | --- | --- | --- | --- | --- | --- | --- |
| S0_1 | 53071 | 60084237 | 1132 | 1886 | 1202 | 451 | 40.48 |
| S0_2 | 49824 | 55932964 | 1122 | 1885 | 1196 | 443 | 40.69 |
| S0_3 | 44785 | 52755753 | 1177 | 1891 | 1243 | 495 | 40.89 |
| S1_1 | 49497 | 56896557 | 1149 | 1903 | 1223 | 458 | 40.82 |
| S1_2 | 50200 | 58482059 | 1164 | 1906 | 1239 | 476 | 40.84 |
| S1_3 | 47783 | 54282099 | 1136 | 1830 | 1189 | 470 | 41.29 |
| S2_1 | 47562 | 54495028 | 1145 | 1841 | 1213 | 478 | 41.14 |
| S2_2 | 49545 | 55326730 | 1116 | 1813 | 1177 | 456 | 41.14 |
| S2_3 | 47556 | 53273046 | 1120 | 1794 | 1186 | 466 | 41 |
| S3_1 | 45910 | 53211707 | 1159 | 1853 | 1228 | 486 | 41.07 |
| S3_2 | 45186 | 53504444 | 1184 | 1886 | 1256 | 504 | 40.93 |
| S3_3 | 47219 | 54129755 | 1146 | 1851 | 1212 | 477 | 41.19 |
| All-Unigene | 105879 | 140733007 | 1329 | 2204 | 1465 | 580 | 40.71 |
