# Supplementary figures and images for: An Integrated Transcriptome and Proteome Analysis Reveals Putative Regulators of Adventitious Root Formation in Taxodium ‘Zhongshanshan’
Source: Int J Mol Sci. 2019 Mar 11;20(5):1225. doi: 10.3390/ijms20051225 (PMC6429173; doi:10.3390/ijms20051225)

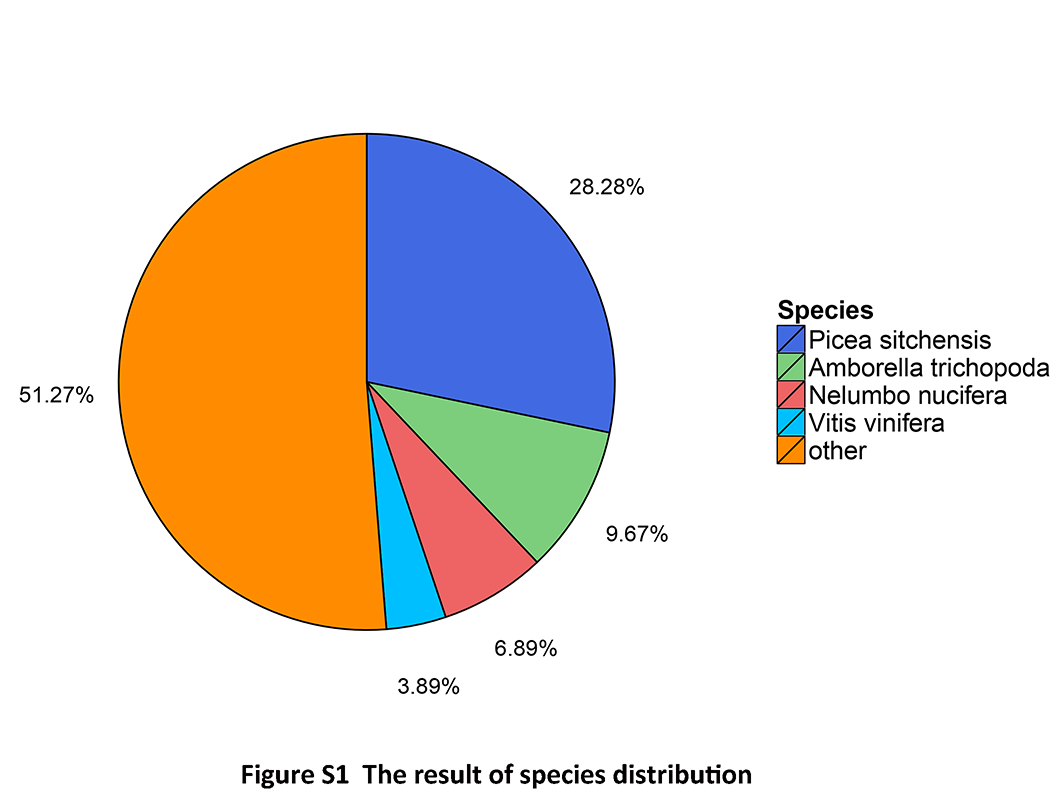

Supplement: Supplementary file 1 [file ijms-20-01225-s001.zip › Supplementary material20190227/Figure S1.tif]

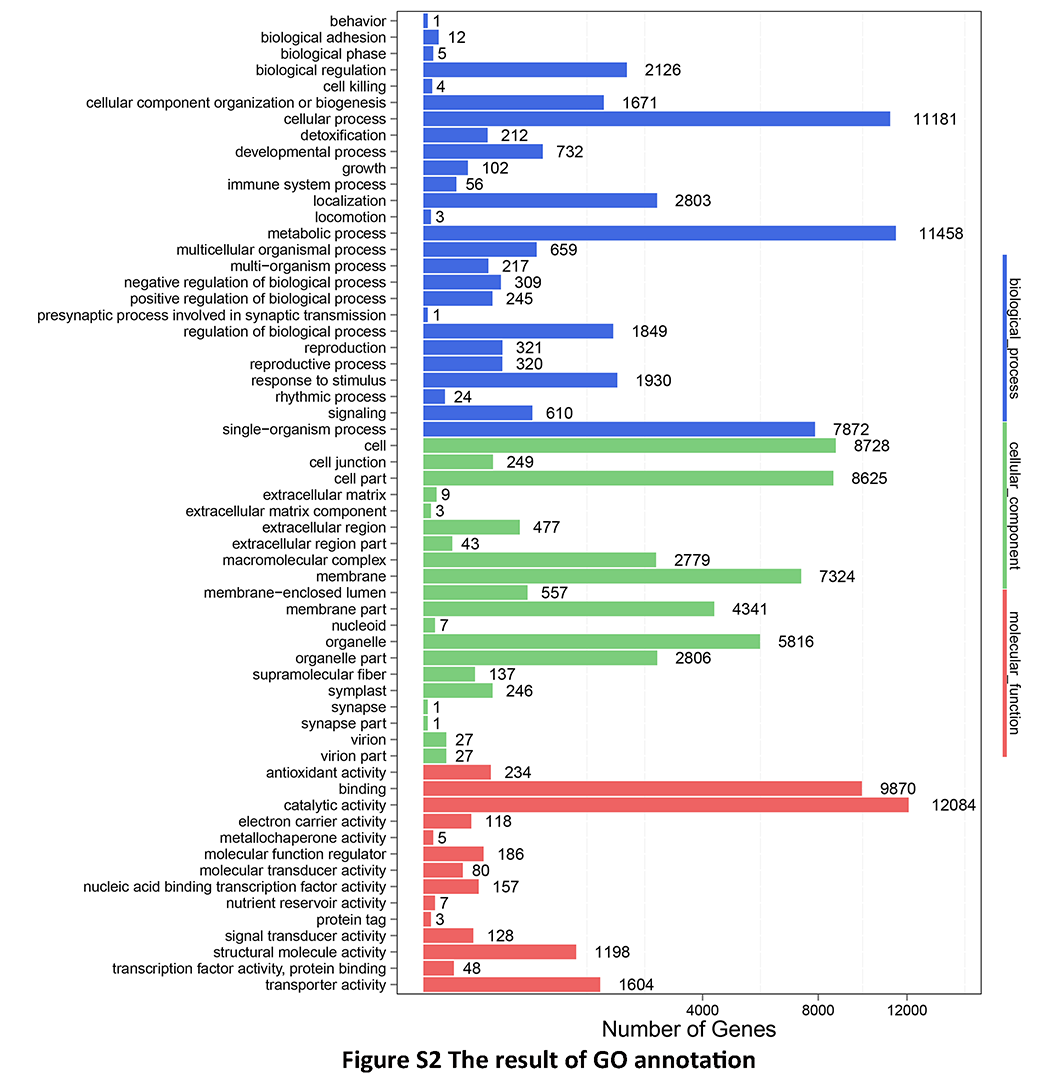

Supplement: Supplementary file 1 [file ijms-20-01225-s001.zip › Supplementary material20190227/Figure S2.tif]

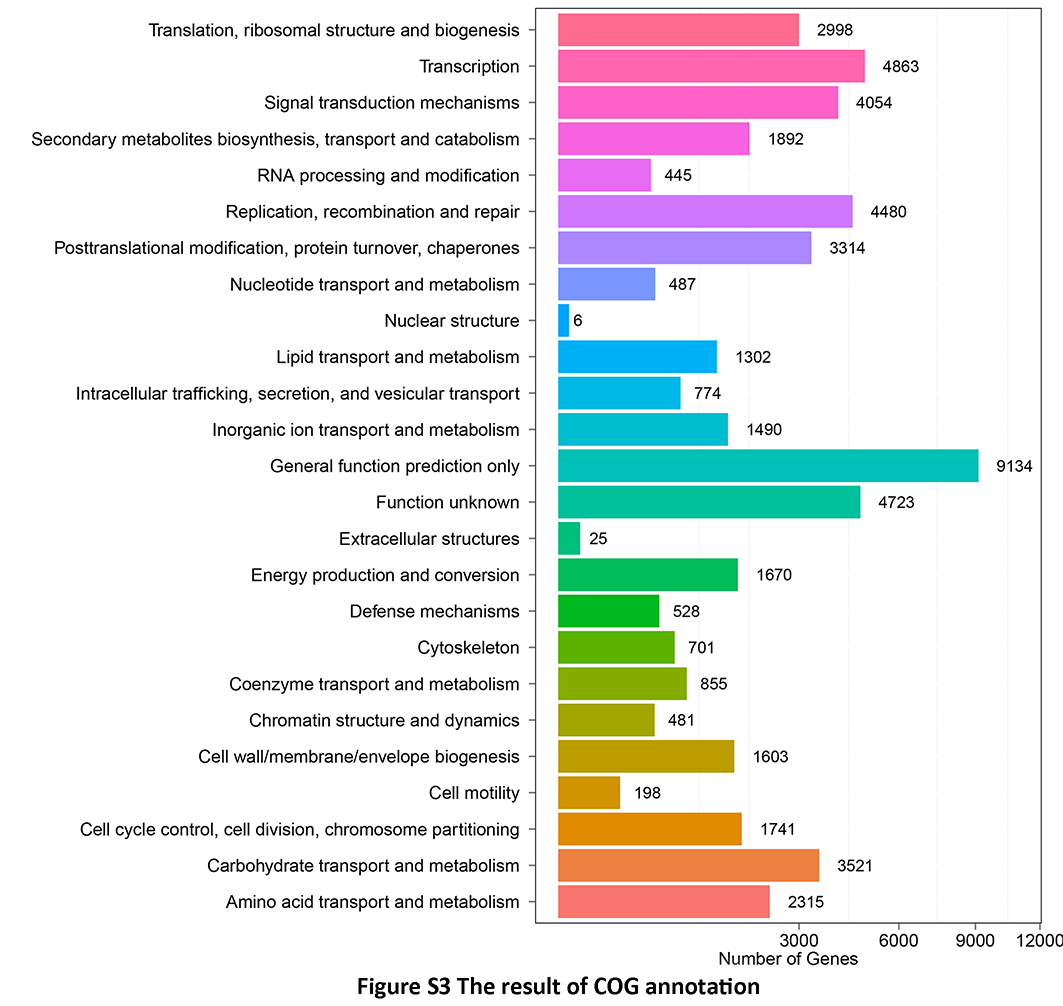

Supplement: Supplementary file 1 [file ijms-20-01225-s001.zip › Supplementary material20190227/Figure S3.tif]

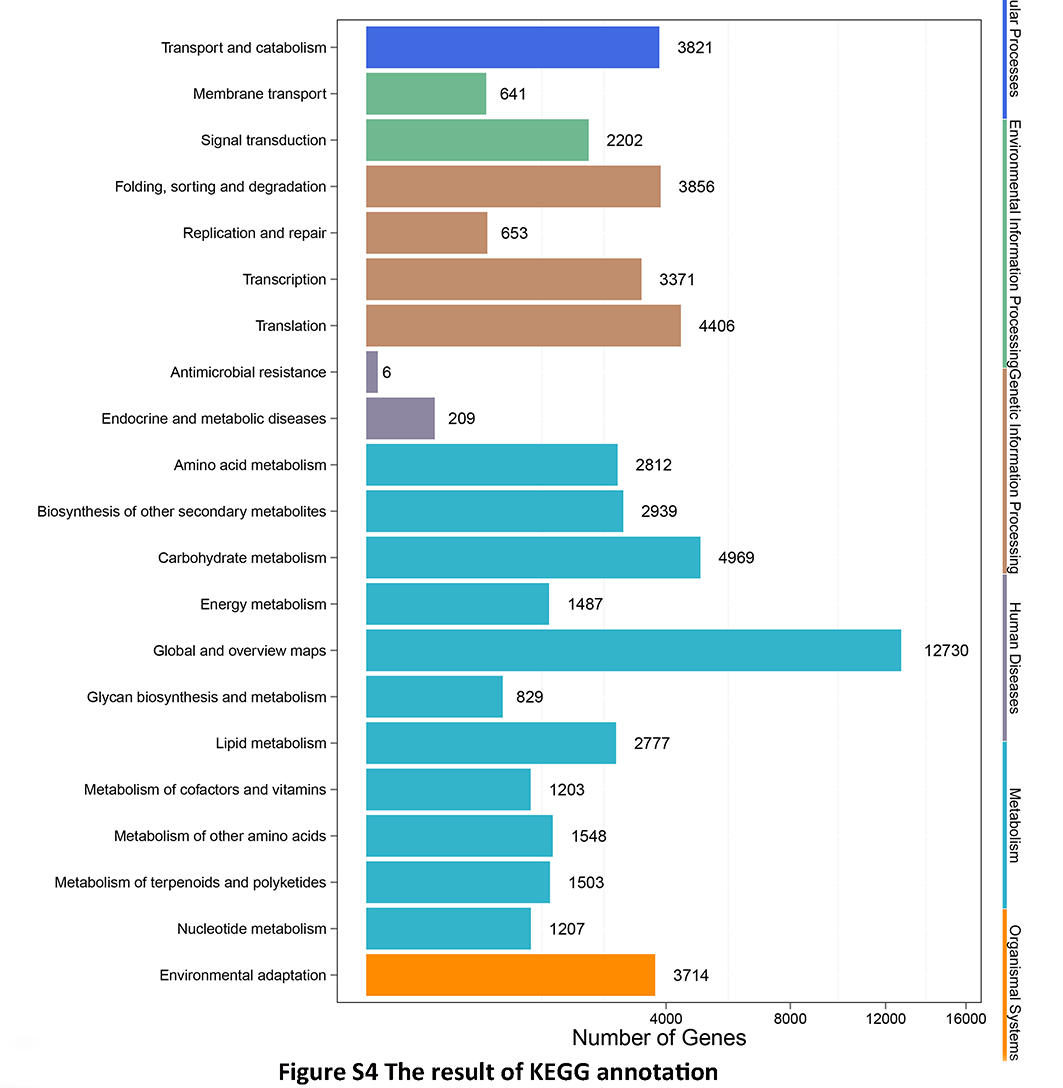

Supplement: Supplementary file 1 [file ijms-20-01225-s001.zip › Supplementary material20190227/Figure S4.tif]

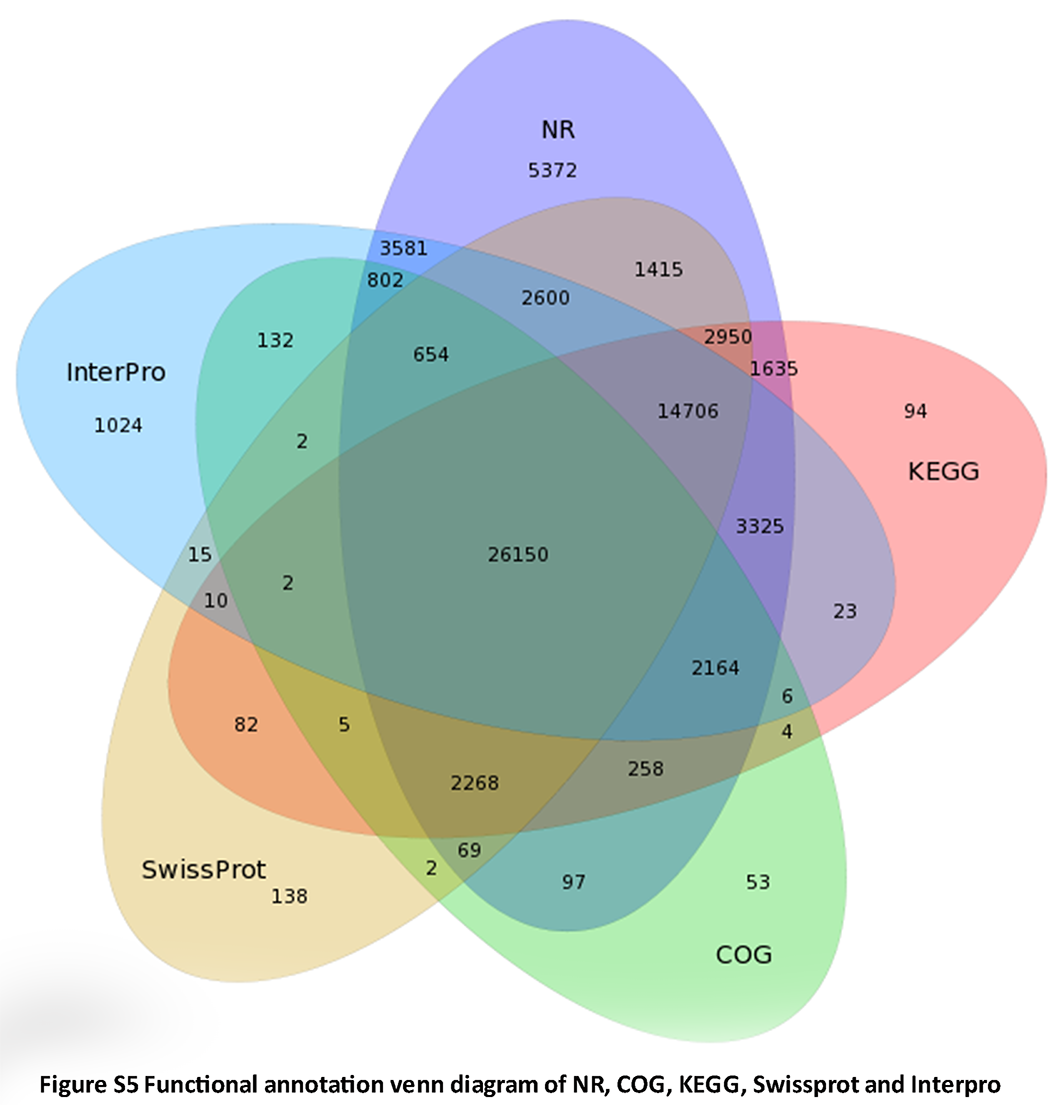

Supplement: Supplementary file 1 [file ijms-20-01225-s001.zip › Supplementary material20190227/Figure S5.tif]

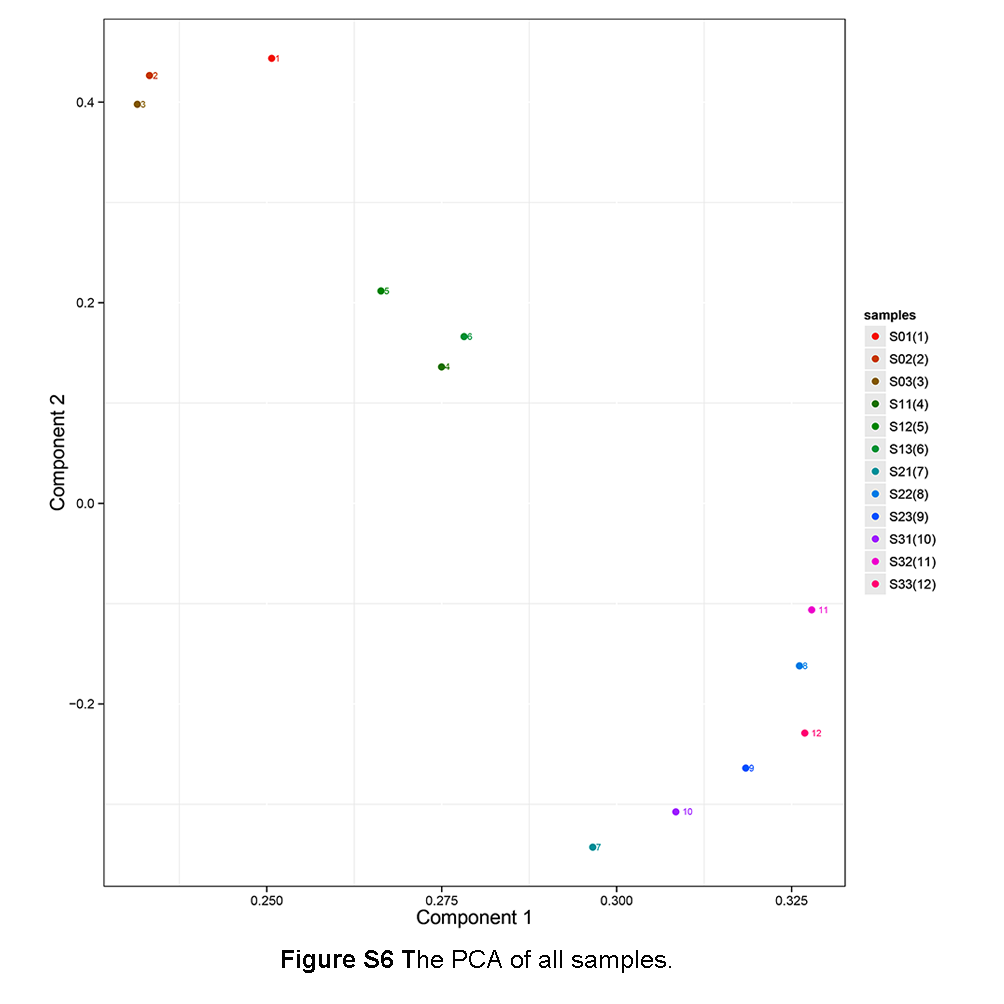

Supplement: Supplementary file 1 [file ijms-20-01225-s001.zip › Supplementary material20190227/Figure S6.tif]

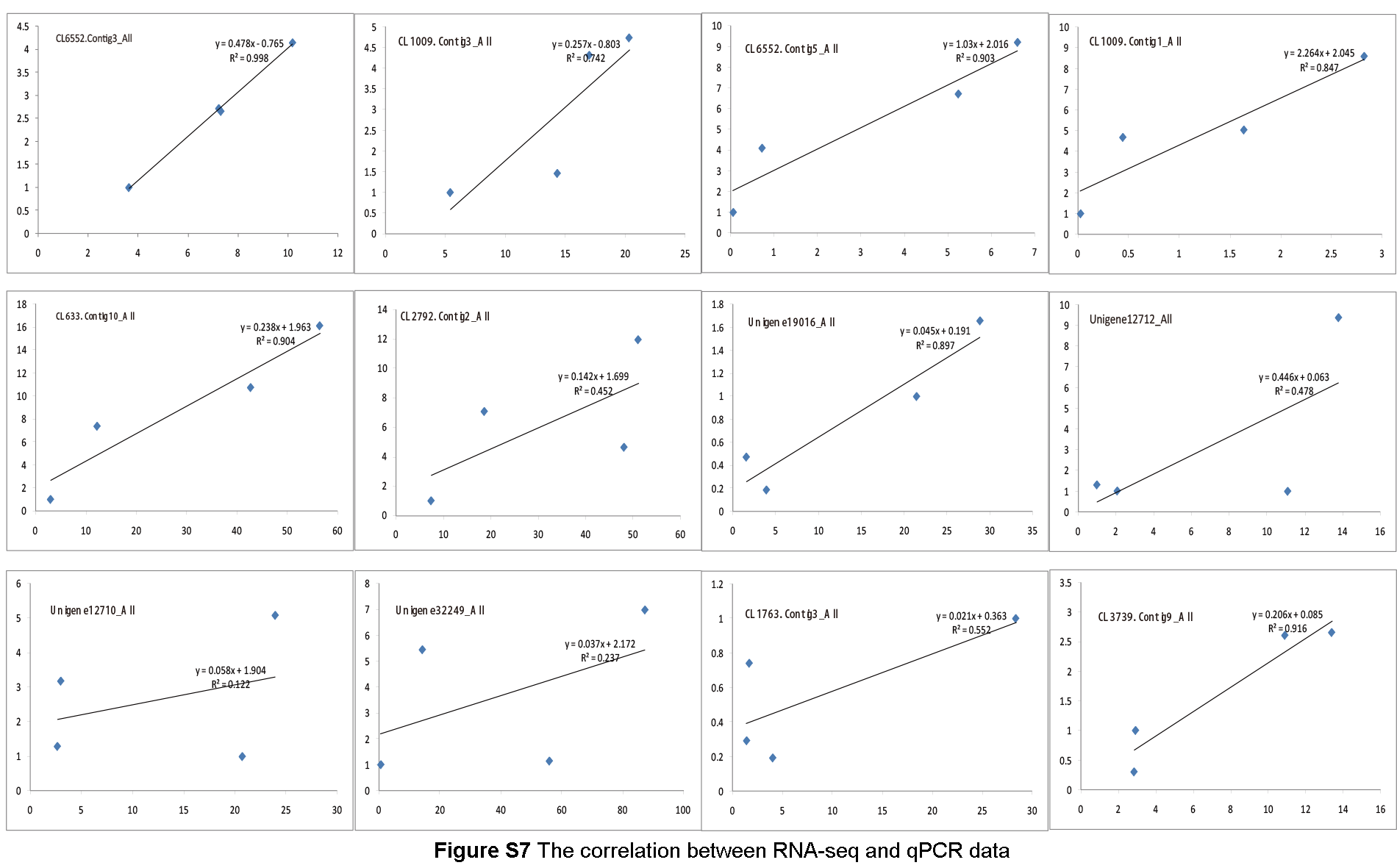

Supplement: Supplementary file 1 [file ijms-20-01225-s001.zip › Supplementary material20190227/Figure S7.tif]

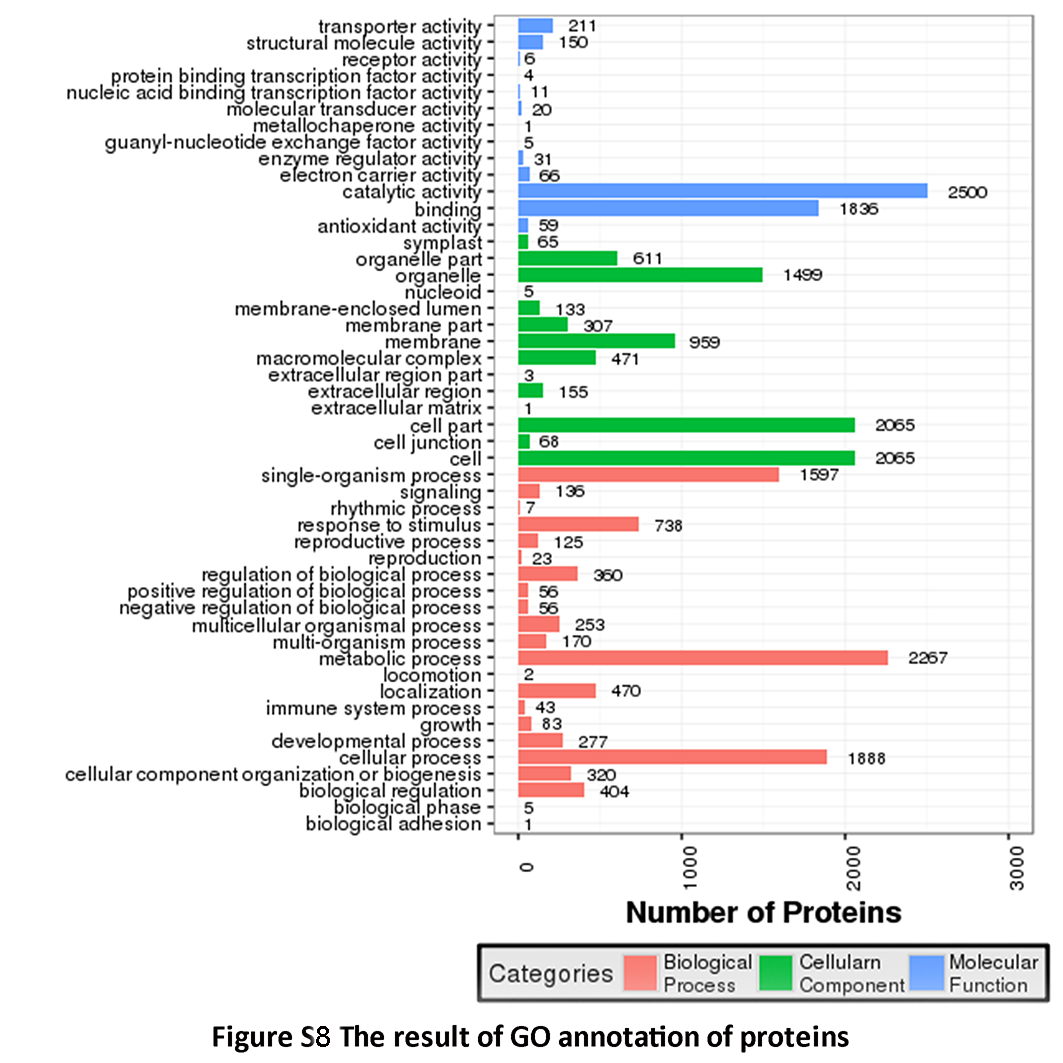

Supplement: Supplementary file 1 [file ijms-20-01225-s001.zip › Supplementary material20190227/Figure S8.tif]

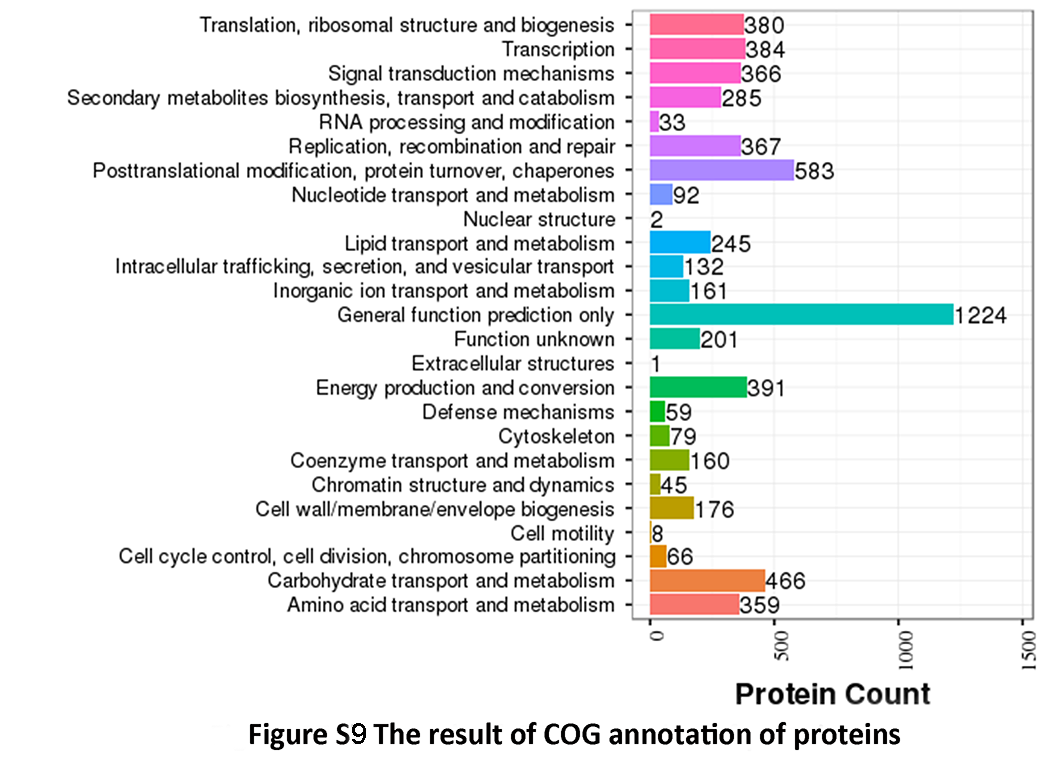

Supplement: Supplementary file 1 [file ijms-20-01225-s001.zip › Supplementary material20190227/Figure S9.tif]
